# Chiral Tertiary Amine Catalyzed Asymmetric [4 + 2] Cyclization of 3-Aroylcoumarines with 2,3-Butadienoate

**DOI:** 10.3390/molecules26020489

**Published:** 2021-01-18

**Authors:** Jun-Lin Li, Xiao-Hui Wang, Jun-Chao Sun, Yi-Yuan Peng, Cong-Bin Ji, Xing-Ping Zeng

**Affiliations:** 1 Key Laboratory of Small Functional Organic Molecule, Ministry of Education, College of Chemistry and Chemical Engineering, Jiangxi Normal University, Nanchang 330022, China; ljl18870471828@163.com (J.-L.L.); 15879027560@163.com (X.-H.W.); jason96chao@163.com (J.-C.S.); yypeng@jxnu.edu.cn (Y.-Y.P.); 2 Jiangxi Provincial Research of Targeting Pharmaceutical Engineering Technology, Shangrao Normal University, Shangrao 334001, China; jcbpxh521@163.com

**Keywords:** coumarins, dihydrocoumarin-fused dihydropyranones, 3-aroylcoumarines, benzyl 2,3-butadienoate, 6’-(4-biphenyl)-β-iso-cinchonine

## Abstract

Coumarins and 2*H*-pyran derivatives are among the most commonly found structural units in natural products. Therefore, the introduction of 2*H*-pyran moiety into the coumarin structural unit, i.e., dihydrocoumarin-fused dihydropyranones, is a potentially successful route for the identification of novel bioactive structures, and the synthesis of these structures has attracted continuing research interest. Herein, a chiral tertiary amine catalyzed [4 + 2] cyclization of 3-aroylcoumarines with benzyl 2,3-butadienoate was reported. In the presence of Kumar’s 6’-(4-biphenyl)-β-iso-cinchonine, the desired dihydrocoumarin-fused dihydropyranone products could be obtained in up to 97% yield and 90% ee values.

## 1. Introduction

Coumarin derivatives are among the most commonly found structural units in natural products, pharmaceuticals, and functional materials [1,2,3,4,5]. Therefore, numerous endeavors have been devoted to develop effective methods for the synthesis of coumarin based compounds [6,7,8,9,10,11]. On the other hand, 2*H*-pyran moieties also play a vital role in natural and unnatural bioactive compounds. Therefore, the introduction of 2*H*-pyran moiety into coumarin structural unit is a highly potential route for the identification of novel bioactive structures and the synthesis of these structures, i.e., dihydrocoumarin-fused dihydropyranones, have attracted continuing research interest. Among the developed methods, the [4 + 2] reaction of 3-aroylcoumarins are the most commonly used [12,13,14].

**Figure 1:**
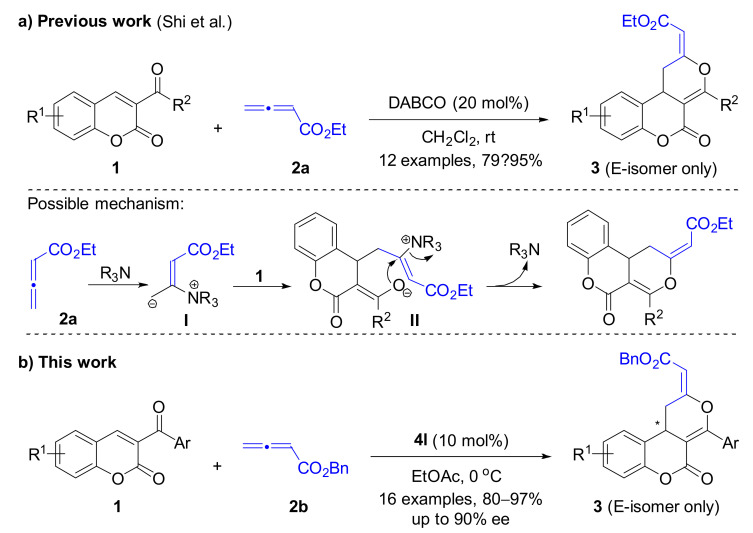
(**a**) DABCO catalyzed [4 + 2] cyclization of 3-aroylcoumarines with 2,3-butadienoate; (**b**) Chiral tertiary amine catalyzed [4 + 2] cyclization of 3-aroylcoumarines with 2,3-butadienoate

Early in 2012, Shi and co-worker described the first [4 + 2] cyclization of 3-aroylcoumarines (**1**) with ethyl 2,3-butadienoate (**2a**) to construct racemic dihydrocoumarin-fused dihydropyranones **3** in 79–95% yield using DABCO as the Lewis base catalyst (Figure 1a) [15]. This [4 + 2] process was initiated by the necleophilic attack of tertiary amine to 2,3-butadienoate to generate zwitterionic **I**. The γ-carbanion of **I** then attacks the β-carbon of enones **1** to give **II** with *Z* configuration to avoid the interaction of the ester group with the 3-position substituent. In the following, an intramolecular nucleophilic substitution of **II** could give cycloadduct **3** and regenerate the tertiary amine catalyst. Soon after that, the Ye group reported that chiral dihydrocoumarin-fused dihydropyranones could be accessed in a highly enantioselective manner via chiral NHC catalyzed [4 + 2] cycloaddition of ketenes and 3-aroylcoumarins [16]. In 2016, Lu et al. achieved a phosphine-catalyzed [4 + 2] annulation of allenones with 3-aroylcoumarins to afford chiral dihydrocoumarin-fused dihydropyrans [17]. Chen and co-workers reported the synthesis of chiral dihydrocoumarin-fused dihydropyrans through dienamine catalysis, but only two examples were explored [18]. Despite the above achievements, the identification of new protocols using easily available starting materials and chiral catalysts for the enantioselective construction of dihydrocoumarin-fused dihydropyrans are still highly desirable.

Inspired by Shi’s pioneering work and based on our interest in the synthesis of chiral coumarin derivatives, we envision that the replacement of DABCO with a suitable chiral tertiary amine catalyst to mediate the [4 + 2] cyclization of 3-aroylcoumarines with 2,3-butadienoate might offer a new method for the synthesis of chiral dihydrocoumarin-fused dihydropyrans (Figure 1b).

## 2. Results and Discussion

We started our investigations by carrying out the reaction between 3-benzoylcoumarine (**1a**) and benzyl 2,3-butadienoate (**2b**) in ClCH_2_CH_2_Cl at 25 °C (Table 1). Initially, a series of cinchona alkaloids, including quinine (**4a**), cinchonine (**4b**), and *C*_2_-symmetric (bis)cinchona alkaloid (**4c**–**f**) were screened (entries 1–6), and the corresponding chiral dihydrocoumarin-fused dihydropyran product (**3a**) was obtained in up to only 45% ee values when **4c** was used, but the yield of **3a** was pretty low even after 48 h (entry 3). To our delight, when the bifunctional β-isocupreidine (**4g**) was tested in our reaction, the reaction was greatly accelerated to complete with 8 h and delivered **3a** in almost quantitative yield with promising 52% ee value (entry 7) [19,20]. Based on this result, we turn our attention to modify β-isocupreidine, so as to improve the enantiocontrol of the reaction. According the methods reported by Kumar and co-workers, a series of 6’-aryl-β-iso-cinchonine (**4h**–**l**) were prepared and examined in the current reaction [21]. It was observed that 6’-phenyl-β-iso-cinchonine **4h** could facilitate the model reaction to give 82% yield for **3a** with improved 60% ee (entry 8). Further variation of the phenyl into more steric aryl groups turned out to be ineffective, as is demonstrated by the 29–35% ee values obtained from catalyst **4i**–**k**. A slightly improved 63% ee was achieved when β-iso-cinchonine (**4l**) bearing a longer 4-biphenyl group at the 6’ position was tried (entry 12), but no more improvement was obtained when further increase the length the substituent (entry 13).

In the following, the solvent effects were examined using **4l** as the catalyst. The reaction was found to be more effective in solvent with moderate polarity (entries 14–21) and EtOAc was found to be the best, which afforded the desired product **3a** in 87% yield and 80% ee (entry 18). In the following, we tried to lower the reaction temperature to 0 °C to improve the ee value of **3a** (entry 22). To our surprise, the reaction finished within 1 h and gave an improved 93% yield, but no improvement of the ee value was observed. The observed higher reactivity at 0 °C than at 25 °C might be attributed to the competitive nucleophilic addition of quinoline nitrogen atoms of the catalyst to 2,3-butadienoate at higher temperature, which deactivate the catalyst and retard the reaction catalytic cycle.

**Figure 2:**
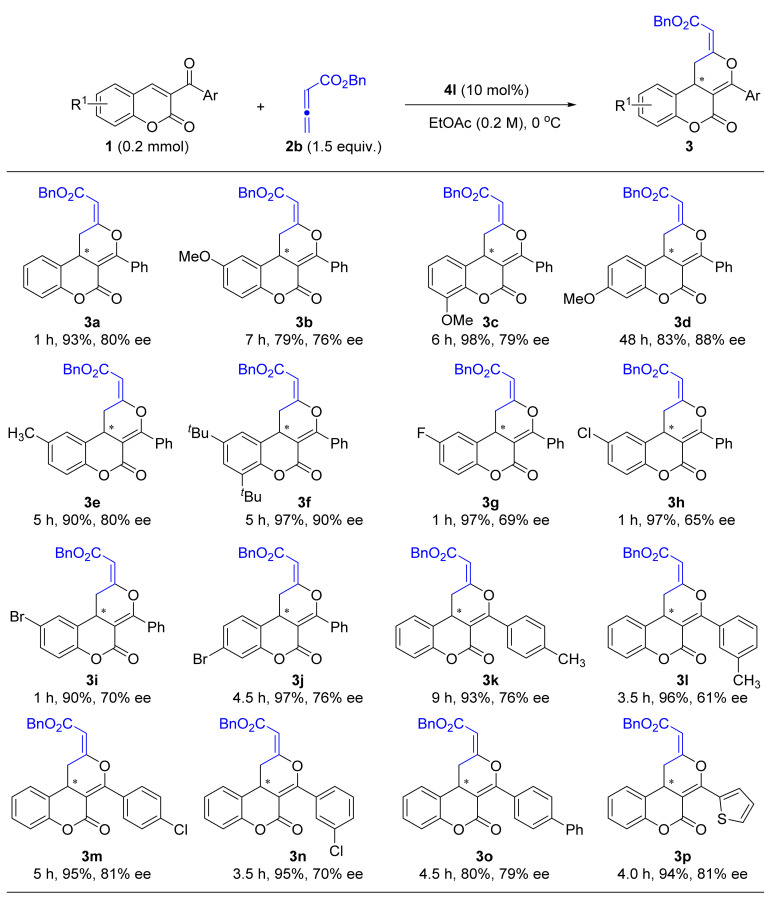
Substrate scope of tertiary amine catalyzed asymmetric [4 + 2] cyclization.

Based on the above optimization, the scope of this tertiary amine catalyzed enantioselective [4 + 2] cyclization of 3-aroylcoumarines with benzyl 2,3-butadienoate (**2b**); we then evaluated this using 10 mol% of **4l** as the catalyst in EtOAc at 0 °C (Figure 2). It was observed that the reaction outcome was significantly affected by the electronic properties of the substituents on the coumarin benzene ring. In general, substrates bearing electron-donating groups (EDG, such as Me, OMe) were relatively less reactive and afforded slightly higher ee values. As is shown by the 79–98% yields and 80–88% ee values for products **3b**–**e**. The more steric **1f** substrate could give the corresponding product **3f** in highest 90% ee under standard conditions. In contrast, the reactions of electron-withdrawing group (EWG) substituted substrates are found to be more reactive but delivered relatively lower enantioselectivities. For example, products **3g**–**i** were obtained in excellent yield but with only around 70% ee. Additionally, coumarins bearing both EDG and EWG on the benzyl ring of the aroyl group were also well tolerated under standard conditions, but the enantiomeric excess was significantly affected by the steric effect. The *para*-substituted products (**3k**,**n**) could be obtained in much higher ee values than the *meta*-substituted products (**3l**,**o**). Moreover, the current reaction is also suitable for the reaction of (1,1’-biphenyl)-4-carbonyl and thiophene-2-carbonyl substituted coumarins, which afforded the desired products **3o** and **3p** in 79% and 81% ee values, respectively.

**Figure 3:**
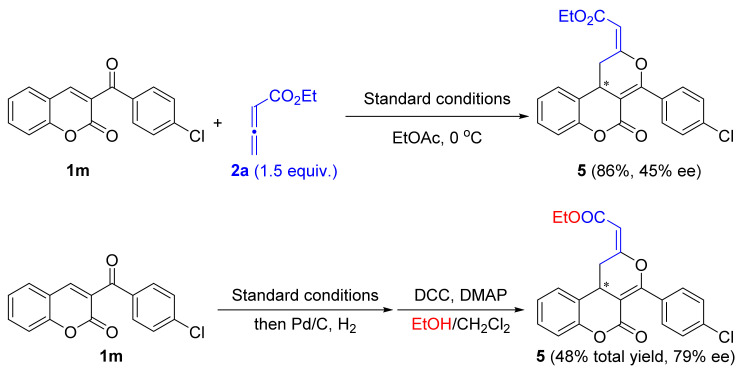
*Z*/*E* Configuration determination.

The *Z*/*E* configuration of the products was determined by the converting product **3m** into the known compound **5** and comparing their NMR spectrum (Figure 3). Under the above optimized conditions, the reaction of 3-benzoyl coumarin **1m** with ethyl 2,3-butadienoate **2a** afforded product **5** with 45% ee value. The same product **5** could also be obtained via a 3-step sequence from **3m** and **2b** in 79% ee value. The NMR spectrum of these newly synthesized products **5** were identical with the previous report by Shi and co-workers. Thus, the configuration of the products **3** were assigned to be *E*. This process also highlighted the synthetic potential of product **1** to be elaborated into other dihydrocoumarin-fused dihydropyran derivatives. We also tried to recrystallize products **3** and determined their absolute configuration by X-ray crystallography analysis, but turned out to be unsuccessful.

**Figure 4:**
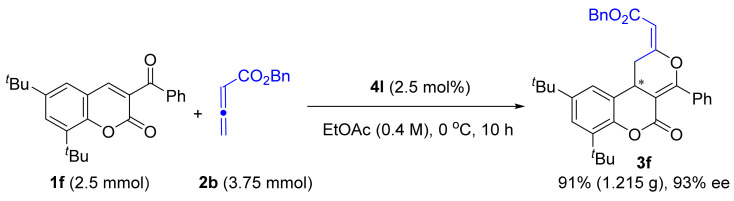
Gram-scale synthesis and product elaboration.

In order to demonstrate the practicability of the current method, we conducted a gram-scale reaction of 3-benzoyl coumarin **1f** with benzyl 2,3-butadienoate **2b** (Figure 4). In the presence of only 2.5 mol% of **4l** as the catalyst, the reaction of 2.5 mmol of **1f** with 1.5 equivalents of **2b** could give rise to the desired dihydrocoumarin-fused dihydropyran **3f** in 91% yield (1.215 g) with slightly improved 93% ee. 

In summary, a series of chiral dihydrocoumarin-fused dihydropyranones were sysnthesized via the enantioselective [4 + 2] cyclization of 3-aroylcoumarines with benzyl 2,3-butadienoate. In the presence of 10 mol% of Kumar’s 6’-(4-biphenyl)-β-iso-cinchonine as the chiral tertiary amine catalyst, the desired products could be obtained in up to 97% yield and 90% ee values under mild conditions. The current method used an easily available chiral catalyst and starting materials and could be conducted on gram-scale without loss of enantiomeric excess. The thus obtained products are potential in the construction of other dihydrocoumarin-fused dihydropyran derivatives. Considering the wide existence of coumarins and 2*H*-pyran moieties in natural products and pharmaceuticals, the thus obtained optically active dihydrocoumarin-fused dihydropyranones should be of interest to medicinal chemists.

## 3. Materials and Methods 

### 3.1. General Information

Reactions were monitored by thin layer chromatography using UV light or KMnO_4_ to visualize the course of reaction. Purification of reaction products was carried out by flash chromatography on silica gel. Chemical yields refer to pure isolated substances. The [α]_D_ was recorded using PolAAr 3005 High Accuracy Polarimeter (Optical Activity Ltd., Huntingdon, England). Infrared (IR) spectra were obtained using a Bruker tensor 27 infrared spectrometer (Bruker, Borken, Germany). ^1^H, ^13^C and ^19^F NMR spectra were obtained using Bruker DPX-400 spectrometer (Bruker UK Limited, Coventry, UK). Chiral HPLC analyses were obtained using Agilent Technologies 1260 Infinity series (Agilent Technologies, Inc., Waldbronn, Germany) and DAICEL CHIRALPAK columns (CPI Company, Tokyo, Japan). Chemical shifts were reported in ppm from tetramethylsilane with the solvent resonance as the internal standard. The following abbreviations were used to designate chemical shift multiplicities: s = singlet, d = doublet, t = triplet, q = quartet, h = heptet, m = multiplet, br = broad.

### 3.2. Tertiary Amine Catalyzed Asymmetric [4 + 2] Cyclization

General procedure: To a 4 mL vial was sequentially added 3-aroylcoumarines **1** (0.2 mmol), catalyst **4l** (0.02 mmol, 10 mol%), and EtOAc (1.0 mL); the mixture was stirred at 0 °C for 15 min before benzyl buta-2,3-dienoate **2b** (0.3 mmol, 1.5 equiv.) was charged. The reaction was monitored by TLC analysis. After completion of the reaction, the solvent was removed by rotary evaporation and the residue was directly subjected to column chromatography using PE/EtOAc (20:1–15:1) as the eluent to afford product **3**.

Benzyl (*E*)-2-(5-oxo-4-phenyl-1,10b-dihydro-2H,5H-pyrano[3,4-c]chromen-2-ylidene) acetate (**3a**).

White solid (m.p. 121.4–122.1 °C); ^1^H NMR (400 MHz, CDCl_3_) δ 7.51–7.31 (m, 12H), 7.20 (t, *J* = 7.2 Hz, 1H), 7.10 (d, *J* = 8.0 Hz, 1H), 5.90 (d, *J* = 1.6 Hz, 1H), 5.23 (s, 2H), 4.87 (dd, *J* = 14.8, 6.0 Hz, 1H), 4.01 (dd, *J* = 12.2, 5.6 Hz, 1H), 2.56–2.49 (m, 1H); ^13^C NMR (101 MHz, CDCl_3_) δ 166.6, 164.2, 162.3, 161.5, 150.8, 136.0, 133.1, 130.9, 129.2, 129.1, 128.8, 128.5, 128.3, 128.3, 125.8, 124.9, 122.7, 117.3, 102.2, 101.5, 66.4, 30.3, 26.1; [α]_D_^26.0^ = + 44.3 (c = 0.26, CHCl_3_); The enantiomeric purity of **3a** was determined by HPLC analysis (DAICEL CHIRALPAK AD–H (0.46 cmφ × 25 cm), hexane:2-propanol = 60:40, flow rate = 0.75 mL/min, retention time: 11.2 min (major) and 14.5 min (minor)); HRMS (ESI): Exact mass calcd for C_27_H_21_O_5_ [M+H]^+^: 425.1389, found: 425.1392.

Benzyl (*E*)-2-(9-methoxy-5-oxo-4-phenyl-1,10b-dihydro-2H,5H-pyrano[3,4-c]chromen-2-ylidene) acetate (**3b**).

White solid (m.p. 131.1–132.8 °C); ^1^H NMR (400 MHz, CDCl_3_) δ 7.51–7.36 (m, 10H), 7.03 (d, *J* = 8.8 Hz, 1H), 6.91 (d, *J* = 2.0 Hz, 1H), 6.85 (dd, *J* = 8.8, 2.8 Hz, 1H), 5.91 (d, *J* = 1.6 Hz, 1H), 5.23 (s, 2H), 4.80 (dd, *J* = 14.6, 5.6 Hz, 1H), 3.98 (dd, *J* = 12.0, 5.6 Hz, 1H), 3.83 (s, 3H), 2.58–2.51(m,1H); ^13^C NMR (101 MHz, CDCl_3_) δ 166.6, 164.1, 162.1, 161.9, 156.8, 144.7, 136.0, 133.0, 130.8, 129.1, 128.8, 128.5, 128.3, 128.3, 123.7, 118.0, 114.2, 111.1, 102.2, 101.4, 66.4 56.0, 30.5, 25.9; [α]_D_^26.0^ = +242.2 (c = 0.49, CHCl_3_); The enantiomeric purity of **3b** was determined by HPLC analysis (DAICEL CHIRALPAK AD-H (0.46 cmφ × 25 cm), hexane:2-propanol = 60:40, flow rate = 0.75 mL/min, retention time: 15.2 min (major) and 21.0 min (minor)); HRMS (ESI): Exact mass calcd for C_28_H_23_O_6_ [M+H]^+^: 455.1495, found: 455.1496.

Benzyl (*E*)-2-(7-methoxy-5-oxo-4-phenyl-1,10b-dihydro-2H,5H-pyrano[3,4-c]chromen-2-ylidene)acetate (**3c**).

White solid (m.p. 108.7–110.2 °C); ^1^H NMR (400 MHz, CDCl_3_) δ 7.53–7.34 (m, 10H), 7.14 (t, *J* = 8.4 Hz, 1H), 6.95 (dd, *J* = 13.0, 8.4 Hz, 2H), 5.91 (d, *J* = 1.2 Hz, 1H), 5.24 (s, 2H), 4.83 (dd, *J* = 14.2, 6.0 Hz, 1H), 4.00 (dd, *J* = 12.4, 6.0 Hz, 1H), 3.90 (s, 3H), 2.57–2.50 (m, 1H); ^13^C NMR (101 MHz, CDCl_3_) δ 166.5, 164.2, 162.1, 161.0, 147.8, 140.2, 136.0, 132.9, 130.8, 129.1, 128.7, 128.4, 128.3, 128.2, 124.7, 123.8, 117.0, 111.7, 102.1, 101.2, 66.3, 56.2, 30.5, 26.0; [α]_D_^26.0^ = +86.5 (c = 0.5, CHCl_3_); The enantiomeric purity of **3c** was determined by HPLC analysis (DAICEL CHIRALPAK AD-H (0.46 cmφ × 25 cm), hexane:2-propanol = 60:40, flow rate = 0.75 mL/min, retention time: 13.0 min (major) and 17.0 min (minor)). HRMS (ESI): Exact mass calcd for C_28_H_23_O_6_ [M+H]^+^: 455.1495, found: 455.1493.

Benzyl (*E*)-2-(8-methoxy-5-oxo-4-phenyl-1,10b-dihydro-2H,5H-pyrano[3,4-c]chromen-2-ylidene)acetate (**3d**).

White solid (m.p. 101.9–102.5 °C); ^1^H NMR (400 MHz, CDCl_3_) δ 7.50–7.34 (m, 10H), 7.27–7.24 (m, 1H), 6.74 (dd, *J* = 8.6, 2.4 Hz, 1H), 6.63 (d, *J* = 2.4 Hz, 1H), 5.87 (d, *J* = 1.6 Hz, 1H), 5.21 (s, 2H), 4.81 (dd, *J* = 14.8, 5.6 Hz, 1H), 3.92 (dd, *J* = 12.4, 5.6 Hz, 1H), 3.80 (s, 3H), 2.47–2.44 (m, 1H); ^13^C NMR (101 MHz, CDCl_3_) δ 166.6, 164.3, 162.2, 161.5, 160.3, 151.5, 136.0, 133.1, 130.8, 129.0, 128.8, 128.4, 128.3, 126.5, 114.5, 111.1, 102.5, 102.0, 101.7, 66.3, 55.7, 29.7, 26.4; [α]_D_^26.0^ = −44.3 (c = 0.26, CHCl_3_); The enantiomeric purity of **3d** was determined by HPLC analysis (DAICEL CHIRALPAK AD-H (0.46 cmφ × 25 cm), hexane:2-propanol = 60:40, flow rate = 0.75 mL/min, retention time: 12.2 min (major) and 17.7 min (minor)). HRMS (ESI): Exact mass calcd for C_28_H_23_O_6_ [M+H]^+^: 455.1495, found: 455.1498.

Benzyl (*E*)-2-(9-methyl-5-oxo-4-phenyl-1,10b-dihydro-2H,5H-pyrano[3,4-c]chromen-2-ylidene)acetate (**3e**).

White solid (m.p. 127.6–128.9 °C); ^1^H NMR (400 MHz, CDCl_3_) δ 7.51–7.35 (m, 10H), 7.18 (s, 1H), 7.12 (d, *J* = 8.4 Hz, 1H), 6.99 (d, *J* = 8.0 Hz, 1H), 5.91 (d, *J* = 1.6 Hz, 1H), 5.24 (s, 2H), 4.87 (dd, *J* = 14.8, 5.6 Hz, 1H), 3.97 (dd, *J* = 12.4, 5.6 Hz, 1H), 2.53–2.46 (m, 1H), 2.37 (s,3H); ^13^CNMR (101 MHz, CDCl_3_) δ 166.7, 164.4, 162.1, 161.7, 148.7, 136.0, 134.6, 133.1, 130.8, 129.7, 129.0, 128.8, 128.5, 128.3, 128.2, 126.2, 122.2, 117.0, 102.0, 101.6, 66.4, 30.2, 26.1, 21.0; [α]_D_^26.0^ = +155.3 (c = 0.50, CHCl_3_); The enantiomeric purity of **3e** was determined by HPLC analysis (DAICEL CHIRALPAK AD-H (0.46 cmφ × 25 cm), hexane:2-propanol = 60:40, flow rate = 0.75 mL/min, retention time: 12.7 min (major) and 18.5 min (minor)). HRMS (ESI): Exact mass calcd for C_28_H_23_O_5_ [M+H]^+^: 439.1545, found: 439.1548.

Benzyl (*E*)-2-(7,9-di-tert-butyl-5-oxo-4-phenyl-1,10b-dihydro-2H,5H-pyrano[3,4-c]chromen-2-ylidene)acetate (**3f**).

Yellow solid (m.p. 83.7–84.9 °C); ^1^H NMR (400 MHz, CDCl_3_) δ 7.54–7.37 (m, 11H), 7.28 (s, 1H), 5.95 (s, 1H), 5.28 (s, 2H), 4.76 (dd, *J* = 14.8, 6.0 Hz, 1H), 4.01 (dd, *J* = 11.6, 6.0 Hz, 1H), 2.77 (t, *J* = 13.2 Hz, 1H), 1.49 (s, 9H), 1.39 (s, 9H); ^13^C NMR (101 MHz, CDCl_3_) δ 166.6, 164.1, 162.1, 161.9, 156.8, 144.7, 136.0, 133.0, 130.9, 129.1, 128.8, 128.5, 128.3, 128.3 123.7, 118.0, 114.2, 111.1, 102.2, 101.4, 66.4, 56.0, 30.5, 25.9; [α]_D_^26.0^ = +58.6 (c = 0.52, CHCl_3_); The enantiomeric purity of **3f** was determined by HPLC analysis (DAICEL CHIRALPAK AD-H (0.46 cmφ × 25 cm), hexane:2-propanol = 60:40, flow rate = 0.75 mL/min, retention time: 6.0 min (major) and 5.1 min (minor)). HRMS (ESI): Exact mass calcd for C_35_H_37_O_5_ [M+H]^+^: 537.2641, found: 537.2643.

Benzyl (*E*)-2-(9-fluoro-5-oxo-4-phenyl-1,10b-dihydro-2H,5H-pyrano[3,4-c]chromen-2-ylidene)acetate (**3g**).

Yellow solid (m.p. 118.4–119.7 °C); ^1^H NMR (400 MHz, CDCl_3_) δ 7.53–7.39 (m, 10H), 7.11–7.00 (m, 3H), 5.93 (d, *J* = 1.6 Hz, 1H), 5.25 (s, 2H), 4.80 (dd, *J* = 14.6, 5.6 Hz, 1H), 3.97 (dd, *J* = 12.4, 5.6 Hz, 1H), 2.56–2.49 (m, 1H); ^13^C NMR (101 MHz, CDCl_3_) δ 166.3, 163.5, 162.6, 161.1, 159.4 (d, ^1^*J* = 243 Hz), 146.7, 135.9, 132.8, 130.9, 129.0, 128.7, 128.4, 128.2, 128.2, 124.3, 124.2, 118.5, 118.4, 115.8 (d, ^2^*J* = 23 Hz), 112.6 (d, ^3^*J* = 25 Hz), 102.4, 100.4, 66.4, 30.3, 25.7; [α]_D_^26.0^ = −22.8 (c = 0.47, CHCl_3_); The enantiomeric purity of **3g** was determined by HPLC analysis (DAICEL CHIRALPAK AD-H (0.46 cmφ × 25 cm), hexane:2-propanol = 60:40, flow rate = 0.75 mL/min, retention time: 11.4 min (major) and 14.1 min (minor)). HRMS (ESI): Exact mass calcd for C_27_H_20_FO_5_ [M+H]^+^: 443.1295, found: 443.1297.

Benzyl (*E*)-2-(9-chloro-5-oxo-4-phenyl-1,10b-dihydro-2H,5H-pyrano[3,4-c]chromen-2-ylidene)acetate (**3h**).

White solid (m.p. 127.4–128.9 °C); ^1^H NMR (400 MHz, CDCl_3_) δ 7.50–7.47 (m, 3H), 7.43–7.40 (m, 8H), 7.39–7.28 (m, 1H), 7.03 (d, *J* = 8.4 Hz, 1H), 5.92 (d, *J* = 2.0 Hz, 1H), 5.24 (s, 2H), 4.83 (dd, *J* = 14.6, 5.6 Hz, 1H), 3.98 (dd, *J* = 12.4, 5.6 Hz, 1H), 2.55–2.47 (m, 1H); ^13^C NMR (101 MHz, CDCl_3_) δ 166.4, 163.5, 162.9, 161.0, 149.3, 135.9, 132.8, 131.0, 130.1, 129.2, 129.0, 128.8, 128.5, 128.3, 128.3, 125.9, 124.3, 118.6, 102.6, 100.4, 66.5, 30.3, 25.8; [α]_D_^26.0^ = −56.8 (c = 0.19, CHCl_3_); The enantiomeric purity of **3h** was determined by HPLC analysis (DAICEL CHIRALPAK AD-H (0.46 cmφ × 25 cm), hexane:2-propanol = 60:40, flow rate = 0.75 mL/min, retention time: 13.7 min (major) and 16.4 min (minor)). HRMS (ESI): Exact mass calcd for C_27_H_20_ClO_5_ [M+H]^+^: 459.0999, found: 459.0997.

Benzyl (*E*)-2-(9-bromo-5-oxo-4-phenyl-1,10b-dihydro-2H,5H-pyrano[3,4-c]chromen-2-ylidene)acetate (**3i**).

White solid (m.p. 131.4–132.7 °C); ^1^H NMR (400 MHz, CDCl_3_) δ 7.51–7.35 (m, 12H), 6.98 (d, J = 8.8 Hz, 1H), 5.92 (d, J = 1.2 Hz, 1H), 5.24 (s, 2H), 4.83 (dd, J = 14.6, 6.0 Hz, 1H), 3.99 (dd, J = 12.2, 6.0 Hz, 1H), 2.55–2.48 (m, 1H); ^13^C NMR (101 MHz, CDCl_3_) δ 166.4, 163.5, 162.9, 160.9, 149.9, 135.9, 132.8, 132.2, 131.0, 129.0, 128.8, 128.8, 128.5, 128.3, 128.3, 124.8, 119.0, 117.6, 102.6, 100.4, 66.5, 30.3, 25.8; [α]_D_^26.0^ = −17.8 (c = 0.22, CHCl_3_); The enantiomeric purity of **3i** was determined by HPLC analysis (DAICEL CHIRALPAK AD-H (0.46 cmφ × 25 cm), hexane:2-propanol = 60:40, flow rate = 0.75 mL/min, retention time: 17.1 min (major) and 14.2 min (minor)). HRMS (ESI): Exact mass calcd for C_27_H_20_BrO_5_ [M+H]^+^: 503.0494, found: 503.0490.

Benzyl (*E*)-2-(8-bromo-5-oxo-4-phenyl-1,10b-dihydro-2H,5H-pyrano[3,4-c]chromen-2-ylidene)acetate (**3j**).

White solid (m.p. 119.2–120.7 °C); ^1^H NMR (400 MHz, CDCl_3_) δ 7.49–7.46 (m, 3H), 7.45–7.29 (m, 8H),7.24–7.20 (m, 2H), 5.90 (d, *J* = 2.0 Hz, 1H), 5.21 (s, 2H), 4.80 (dd, *J* = 14.8, 5.6 Hz, 1H), 3.90 (dd, *J* = 12.2, 5.6 Hz, 1H), 2.51–2.44 (m, 1H); ^13^C NMR (101 MHz, CDCl_3_) δ 166.4, 163.6, 162.8, 160.7, 151.2, 135.9, 132.7, 130.9, 129.0, 128.7, 128.4, 128.3, 127.8, 127.1, 122.0, 121.8, 120.4, 102.4, 100.5, 66.4, 30.0, 25.8; [α]_D_^26.0^ = +67.4 (c = 0.52, CHCl_3_); The enantiomeric purity of **3j** was determined by HPLC analysis (DAICEL CHIRALPAK AD-H (0.46 cmφ × 25 cm), hexane:2-propanol = 60:40, flow rate = 0.75 mL/min, retention time: 14.7 min (major) and 18.0 min (minor)). HRMS (ESI): Exact mass calcd for C_27_H_20_BrO_5_ [M+H]^+^: 503.0494, found: 503.0497.

Benzyl (*E*)-2-(5-oxo-4-(p-tolyl)-1,10b-dihydro-2H,5H-pyrano[3,4-c]chromen-2-ylidene)acetate (**3k**).

Yellow solid (m.p. 133.2–133.9 °C); ^1^H NMR (400 MHz, CDCl_3_) δ 7.44–7.32 (m, 9H), 7.24–7.19 (m, 3H), 7.11 (d, *J* = 8.0 Hz, 1H), 5.91 (d, *J* = 1.2 Hz, 1H), 5.24 (s, 2H), 4.87 (dd, *J* = 14.8, 5.6 Hz, 1H), 3.99 (dd, *J* = 12.2, 6.0 Hz, 1H), 2.55–2.48 (m, 1H), 2.41 (s, 3H); ^13^C NMR (101 MHz, CDCl_3_) δ 166.5, 164.3, 162.4, 161.7, 150.8, 141.3, 136.0, 130.0, 129.1, 129.0, 128.7, 128.4, 128.3, 125.7, 124.8, 122.8, 117.2, 102.0, 100.9, 66.3, 30.3, 26.0, 21.7; [α]_D_^26.0^ = +32.2 (c = 0.54, CHCl_3_); The enantiomeric purity of **3k** was determined by HPLC analysis (DAICEL CHIRALPAK AD-H (0.46 cmφ × 25 cm), hexane:2-propanol = 60:40, flow rate = 0.75 mL/min, retention time: 12.1 min (major) and 18.0 min (minor)). HRMS (ESI): Exact mass calcd for C_28_H_23_O_5_ [M+H]^+^: 439.1545, found: 439.1543.

Benzyl (*E*)-2-(5-oxo-4-(m-tolyl)-1,10b-dihydro-2H,5H-pyrano[3,4-c]chromen-2-ylidene)acetate (**3l**).

Yellow solid (m.p. 135.6–136.8 °C); ^1^H NMR (400 MHz, CDCl_3_) δ 7.43–7.31 (m, 9H), 7.24–7.19 (m, 3H), 7.11 (d, *J* = 8.4 Hz, 1H), 5.91 (d, *J* = 1.2 Hz, 1H), 5.24 (s, 2H), 4.87 (dd, J = 14.8, 6.0 Hz, 1H), 3.99 (dd, *J* = 12.4, 5.6 Hz, 1H). 2.55–2.48 (m, 1H), 2.41 (s, 3H); ^13^C NMR (101 MHz, CDCl_3_) δ 166.5, 164.3, 162.4, 161.7, 150.8, 141.3, 136.0, 130.0, 129.1, 129.0, 128.7, 128.4, 128.3, 125.7, 124.8, 122.8, 117.2, 102.0, 100.9, 66.3, 30.3, 26.0, 21.7; [α]_D_^26.0^ = +42.2 (c = 0.52, CHCl_3_); The enantiomeric purity of **3l** was determined by HPLC analysis (DAICEL CHIRALPAK AD-H (0.46 cmφ × 25 cm), hexane:2-propanol = 60:40, flow rate = 0.75 mL/min, retention time: 9.9 min (major) and 13.2 min (minor)). HRMS (ESI): Exact mass calcd for C_28_H_23_O_5_ [M+H]^+^: 439.1545, found: 439.1544.

Benzyl (*E*)-2-(4-(4-chlorophenyl)-5-oxo-1,10b-dihydro-2H,5H-pyrano[3,4-c]chromen-2-ylidene)acetate (**3m**).

Yellow solid (m.p. 122.5–123.7 °C); ^1^H NMR (400 MHz, CDCl_3_) δ 7.46–7.32 (m, 11H), 7.23–7.19 (m, 1H), 7.10 (d, *J* = 8.0 Hz, 1H), 5.90 (d, *J* = 1.6 Hz, 1H), 5.23 (s, 2H), 4.86 (dd, *J* = 14.8, 6.0 Hz, 1H), 4.00 (dd, *J* = 12.4, 5.6 Hz, 1H), 2.55–2.48 (m, 1H); ^13^C NMR (101 MHz, CDCl_3_) δ 166.4, 163.9, 161.4, 161.1, 150.7, 137.0, 136.0, 130.5, 129.3, 128.8, 128.6, 128.5, 128.3, 125.8, 125.0, 122.5, 117.3, 102.3, 101.9, 66.4, 30.3, 26.0; [α]_D_^26.0^ = −148.3 (c = 0.49, CHCl_3_); The enantiomeric purity of **3m** was determined by HPLC analysis (DAICEL CHIRALPAK AD-H (0.46 cmφ × 25 cm), hexane:2-propanol = 60:40, flow rate = 0.75 mL/min, retention time: 13.9 min (major) and 21.5 min (minor)). HRMS (ESI): Exact mass calcd for C_27_H_20_ClO_5_ [M+H]^+^: 459.0999, found: 459.0999.

Benzyl (*E*)-2-(4-(3-chlorophenyl)-5-oxo-1,10b-dihydro-2H,5H-pyrano[3,4-c]chromen-2-ylidene)acetate (**3n**).

Yellow solid (m.p. 124.5–125.3 °C); ^1^H NMR (400 MHz, CDCl_3_) δ 7.49–7.34 (m, 11H), 7.23–7.21 (m, 1H), 7.11–7.09 (m, 1H), 5.92 (d, *J* = 2.0 Hz, 1H), 5.24 (d, *J* = 2.0 Hz, 2H), 4.90–4.85 (m, 1H), 4.01–3.98 (m, 1H), 2.52 (t, *J* = 12.8, 1H); ^13^C NMR (101 MHz, CDCl_3_) δ 166.3, 163.8, 161.1, 160.6, 150.6, 135.9, 134.7, 134.2, 131.4, 130.8, 129.5, 129.2, 129.0, 128.7, 128.4, 128.3, 127.4, 125.8, 125.0, 122.3, 117.2, 102.4, 102.2, 66.4, 30.2, 25.9; [α]_D_^26.0^ = +100.7 (c = 0.55, CHCl_3_); The enantiomeric purity of **3n** was determined by HPLC analysis (DAICEL CHIRALPAK AD-H (0.46 cmφ × 25 cm), hexane:2-propanol = 60:40, flow rate = 0.75 mL/min, retention time: 12.4 min (major) and 13.9 min (minor)). HRMS (ESI): Exact mass calcd for C_27_H_20_ClO_5_ [M+H]^+^: 459.0999, found: 459.1003.

Benzyl (*E*)-2-(4-([1,1’-biphenyl]-4-yl)-5-oxo-1,10b-dihydro-2H,5H-pyrano[3,4-c]chromen-2-ylidene)acetate (**3o**).

White solid (m.p. 130.7–131.4 °C); ^1^H NMR (400 MHz, CDCl_3_) δ 7.66–7.60(m, 6H), 7.49–7.33 (m, 10H), 7.22 (t, *J* = 7.6 Hz, 1H), 7.13 (d, *J* = 8.0 Hz, 1H), 5.95 (s, 1H), 5.26 (s, 2H), 4.89 (dd, *J* = 14.8, 5.6 Hz, 1H), 4.03 (dd, J = 12.0, 5.6 Hz, 1H), 2.55 (t, *J* = 14.0 Hz, 1H); ^13^C NMR (101 MHz, CDCl_3_) δ 166.5, 164.2, 162.0, 161.6, 150.8, 143.7, 140.3, 136.0, 129.6, 129.1, 128.9, 128.7, 128.4, 128.3, 127.3, 126.9, 125.8, 117.2, 102.1, 101.4, 66.3, 30.3, 26.0; [α]_D_^26.0^ = +91.1 (c = 0,34, CHCl_3_); The enantiomeric purity of **3o** was determined by HPLC analysis (DAICEL CHIRALPAK AD-H (0.46 cmφ × 25 cm), hexane:2-propanol = 60:40, flow rate = 0.75 mL/min, retention time: 17.9 min (major) and 31.4 min (minor)). HRMS (ESI): Exact mass calcd for C_33_H_25_O_5_ [M+H]^+^: 501.1702, found: 501.1708.

Benzyl (*E*)-2-(5-oxo-4-(thiophen-3-yl)-1,10b-dihydro-2H,5H-pyrano[3,4-c]chromen-2-ylidene)acetate (**3p**).

White solid (m.p. 135.7–136.2 °C); ^1^H NMR (400 MHz, CDCl_3_) δ 7.83 (d, *J* = 3.6 Hz, 1H), 7.53 (d, *J* = 5.2 Hz, 1H), 7.41–7.30 (m, 7H), 7.18 (t, *J* = 7.6 Hz, 1H), 7.09 (dd, J = 8.4, 4.8 Hz, 2H), 5.92 (d, *J* = 1.2 Hz, 1H), 5.27–5.20 (m,2H), 4.70 (dd, *J* = 15.0, 6.0 Hz, 1H), 4.02 (dd, J = 12.6, 5.6 Hz, 1H), 2.69–2.62 (m,1H); ^13^C NMR (101 MHz, CDCl_3_) δ 166.5, 164.0, 163.0, 161.6, 155.2, 150.6, 136.0, 133.6, 132.8, 130.7, 129.1, 128.8, 128.5, 128.3, 127.3, 125.7, 124.9, 122.6, 117.1, 101.8, 100.3, 66.4, 30.9, 29.8, 26.4; [α]_D_^26.0^ = +123.7 (c = 0.22, CHCl_3_); The enantiomeric purity of **3p** was determined by HPLC analysis (DAICEL CHIRALPAK AD-H (0.46 cmφ × 25 cm), hexane:2-propanol = 60:40, flow rate = 0.75 mL/min, retention time: 12.4 min (major) and 14.6 min (minor)). HRMS (ESI): Exact mass calcd for C_25_H_19_O_5_S [M+H]^+^: 459.0999, found: 459.1003.

### 3.3. Synthesis of ***5*** from ***1m*** and ***2b***

To the reaction mixture obtained under standard condition using **1m** (1 mmol) and **2b** (1.25 mmol) was added Pd/C (wt. 10%), then the mixture was stirred under H_2_ atmosphere (H_2_ balloon) at room temperature for 48 h. The resulting reaction mixture was filtered through a pad of Celite and eiluted with EtOAc. The filtration was concentrated under reduced pressure and the residue was purified by silica gel column chromatography (PE:EtOAc = 5:1–2:1) to afford the free acid intermediate, which was then dissolved in CH_2_Cl_2_ (5 mL). This solution was cooled to 0 °C before DCC (2.0 equiv.), DMAP (2.0 equiv.) and EtOH (2 mL) were added. After that, the reaction mixture was moved to rt and stirred overnight. After completion of the reaction by TLC analysis, the solvent was removed by rotary evaporation and the residue was directly subjected to column chromatography using PE/EtOAc (15:1-9:1) as the eluent to afford product **5**.

Ethyl (E)-2-(4-(4-chlorophenyl)-5-oxo-1,10b-dihydro-2H,5H-pyrano[3,4-c]chromen-2-ylidene)acetate (**5**) [4].

^1^H NMR (400 MHz, CDCl_3_) δ 7.46–7.31 (m, 6H), 7.21 (t, *J* = 7.6 Hz, 1H), 7.09 (d, *J* = 8.0 Hz, 1H), 5.83 (s, 1H), 4.87 (dd, *J* = 14.8, 5.6 Hz, 1H), 4.24 (q, *J* = 7.2 Hz, 2H), 4.00 (dd, *J* = 12.0, 5.6 Hz, 1H), 2.50 (t, *J* = 14.4 Hz, 1H), 1.33 (t, *J* = 7.2 Hz, 3H); ^13^C NMR (101 MHz, CDCl_3_) δ 166.6, 163.4, 161.5, 161.1, 150.6, 136.9, 131.4, 130.5, 129.2, 128.6, 125.8, 125.0, 122.5, 117.2, 102.6, 101.7, 60.6, 30.3, 25.8, 14.4; [α]_D_^26.0^ = +78.6 (c = 0.38, CHCl_3_); The enantiomeric purity of **5** was determined by HPLC analysis (DAICEL CHIRALPAK OD-3 (4.6 mmφ × 150 mml), hexane:2-propanol = 80:20, flow rate = 0.75 mL/min, retention time: 6.1 min (major) and 7.6 min (minor)).

## Figures and Tables

**Table 1:**
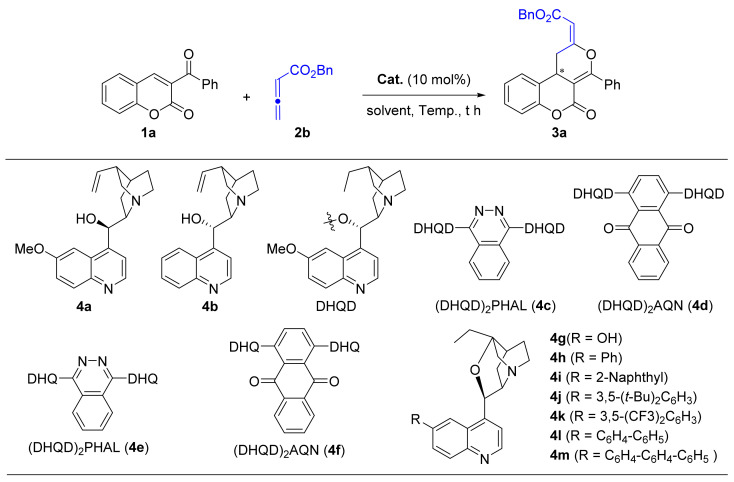
Condition optimization for the catalytic asymmetric [4 + 2] cyclization.

Entry	Cat.	Solvent	Temp. (°C)	t (h)	Yield (%)	Ee (%)
1	Quinine (**4a**)	DCE	25	48	6	39
2	Cinchonine (**4b**)	DCE	25	48	33	16
3	**4c**	DCE	25	48	8	45
4	**4d**	DCE	25	48	83	27
5	**4e**	DCE	25	48	trace	
6	**4f**	DCE	25	48	23	22
7	**4g**	DCE	25	8	96	52
8	**4h**	DCE	25	11	82	60
9	**4i**	DCE	25	23	78	35
10	**4j**	DCE	25	23	60	34
11	**4k**	DCE	25	23	67	29
12	**4l**	DCE	25	11	87	63
13	**4m**	DCE	25	16	78	47
14	**4l**	*n*-C_6_H_12_	25	48	trace	
15	**4l**	toluene	25	48	68	79
16	**4l**	THF	25	48	77	66
17	**4l**	Acetone	25	2.5	79	58
18	**4l**	EtOAc	25	7.5	87	79
19	**4l**	CH_3_CN	25	2.5	95	56
20	**4l**	DMF	25	2.5	88	53
21	**4l**	MeOH	25	48	trace	--
22	**4l**	EtOAc	0	1	93	79

## Data Availability

Samples of the compounds are not available from the authors.

## Supplementary Materials

The following are available online: ^1^H and ^13^C NMR spectra and HPLC data of compounds **3a-p** and **5**.

## Sample Availability

Samples of the compounds are not available from the authors.
