# Supplementary material for: Chiral Tertiary Amine Catalyzed Asymmetric [4 + 2] Cyclization of 3-Aroylcoumarines with 2,3-Butadienoate
Source: Molecules. 2021 Jan 18;26(2):489. doi: 10.3390/molecules26020489 (PMC7831925; doi:10.3390/molecules26020489)
Supplement: Supplementary file 1 [file molecules-26-00489-s001.pdf]

Chiral Tertiary Amine Catalyzed Asymmetric [4+2] Cyclization of 3-Aroylcoumarines with 2,3-Butadienoate

Jun-Lin Li [1], Xiao-Hui Wang [1], Jun-Chao Sun[1], Yi-Yuan Peng[1], Cong-Bin Ji[2] and Xing-Ping Zeng[1]*

[1] Key Laboratory of Small Functional Organic Molecule, College of Chemistry and Chemical Engineering, Jiangxi Normal University, Nanchang, Jiangxi 330022, China
[2] Jiangxi Provincial Research of Targeting Pharmaceutical Engineering Technology, Shangrao Normal University, Shangrao, Jiangxi, 334001, China
* Correspondence: 005173@jxnu.edu.cn;

**Contents**	Page
**1. NMR Spectrum**	S2-S35
**2. HLPC spectrum**	S36-S52

^{1}H NMR Spectrum (400 MHz, Chloroform-*d*) of **3a**

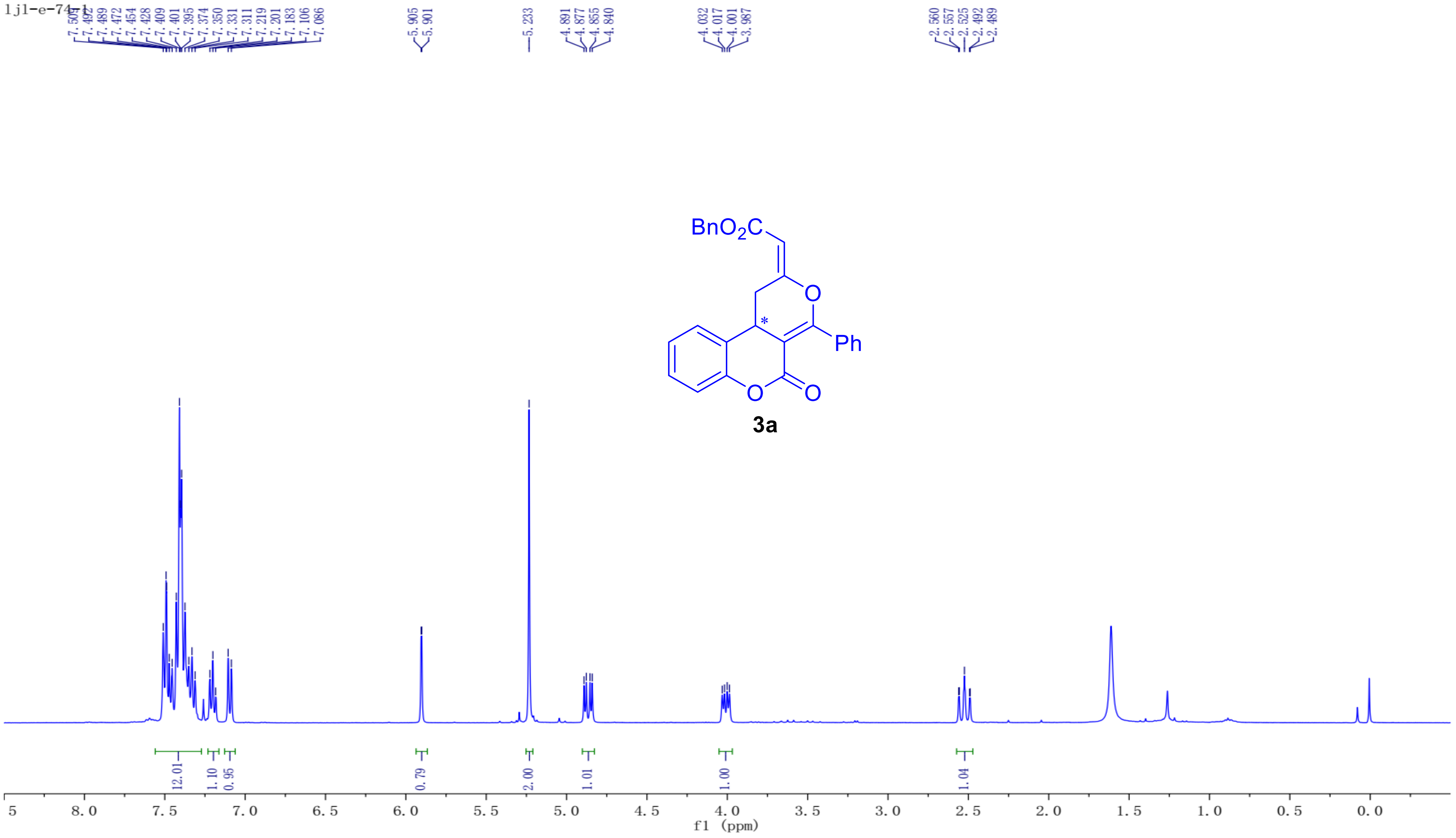

^{13}C {^{1}H} NMR Spectrum (101 MHz, Chloroform-*d*) of **3a**

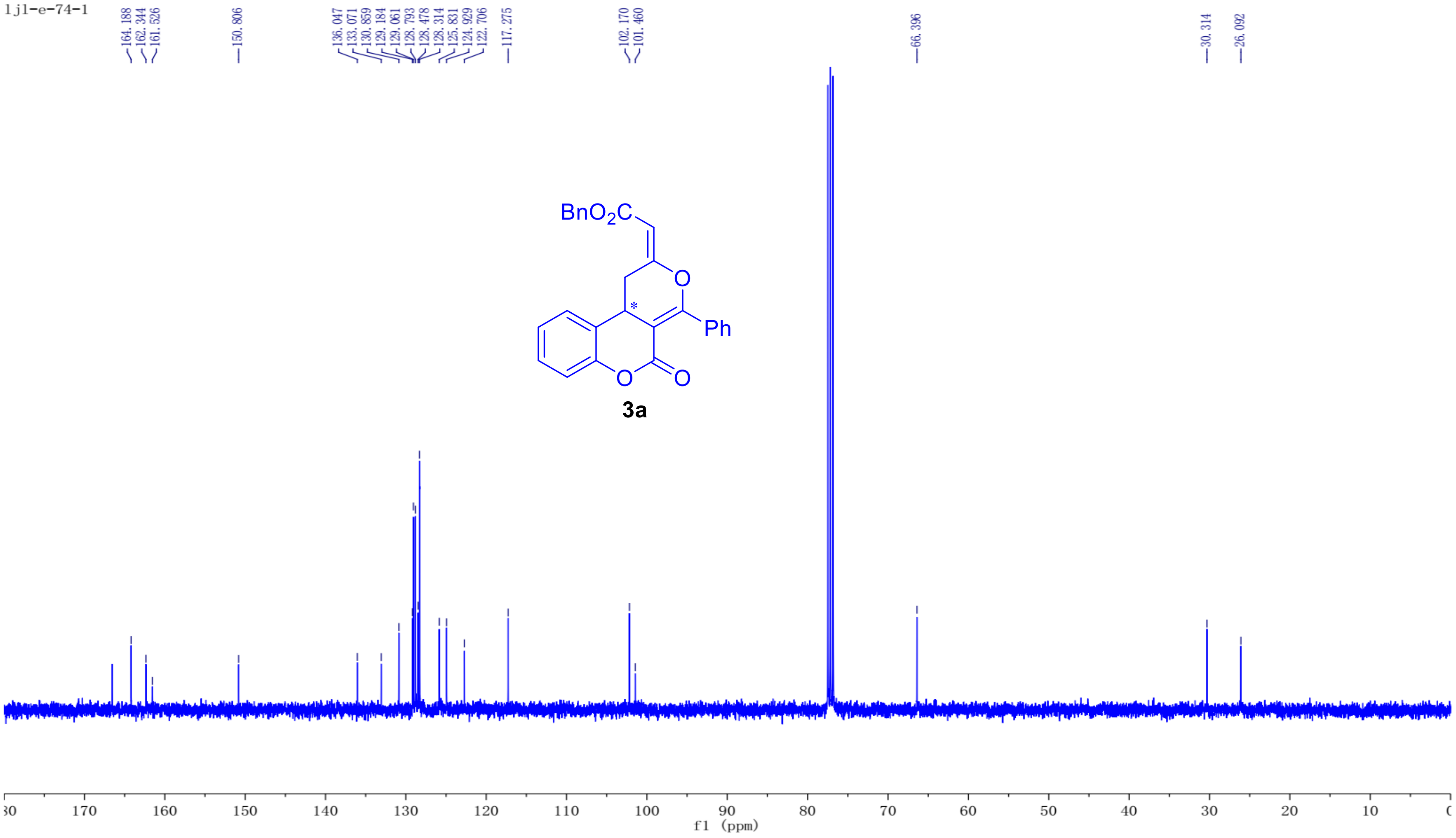

^{1}H NMR Spectrum (400 MHz, Chloroform-*d*) of **3b**

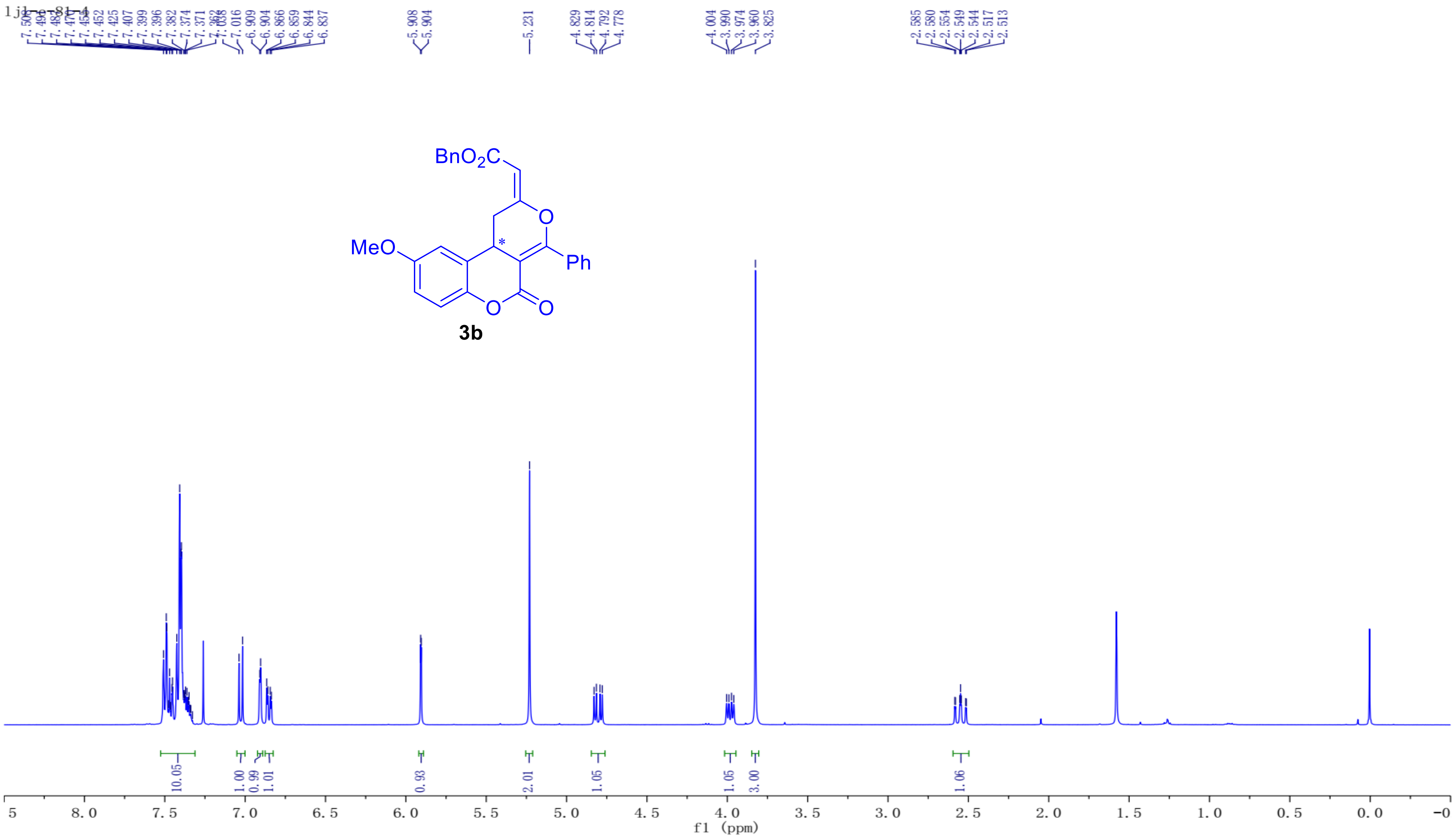

^{13}C {^{1}H} NMR Spectrum (101 MHz, Chloroform-*d*) of **3b**

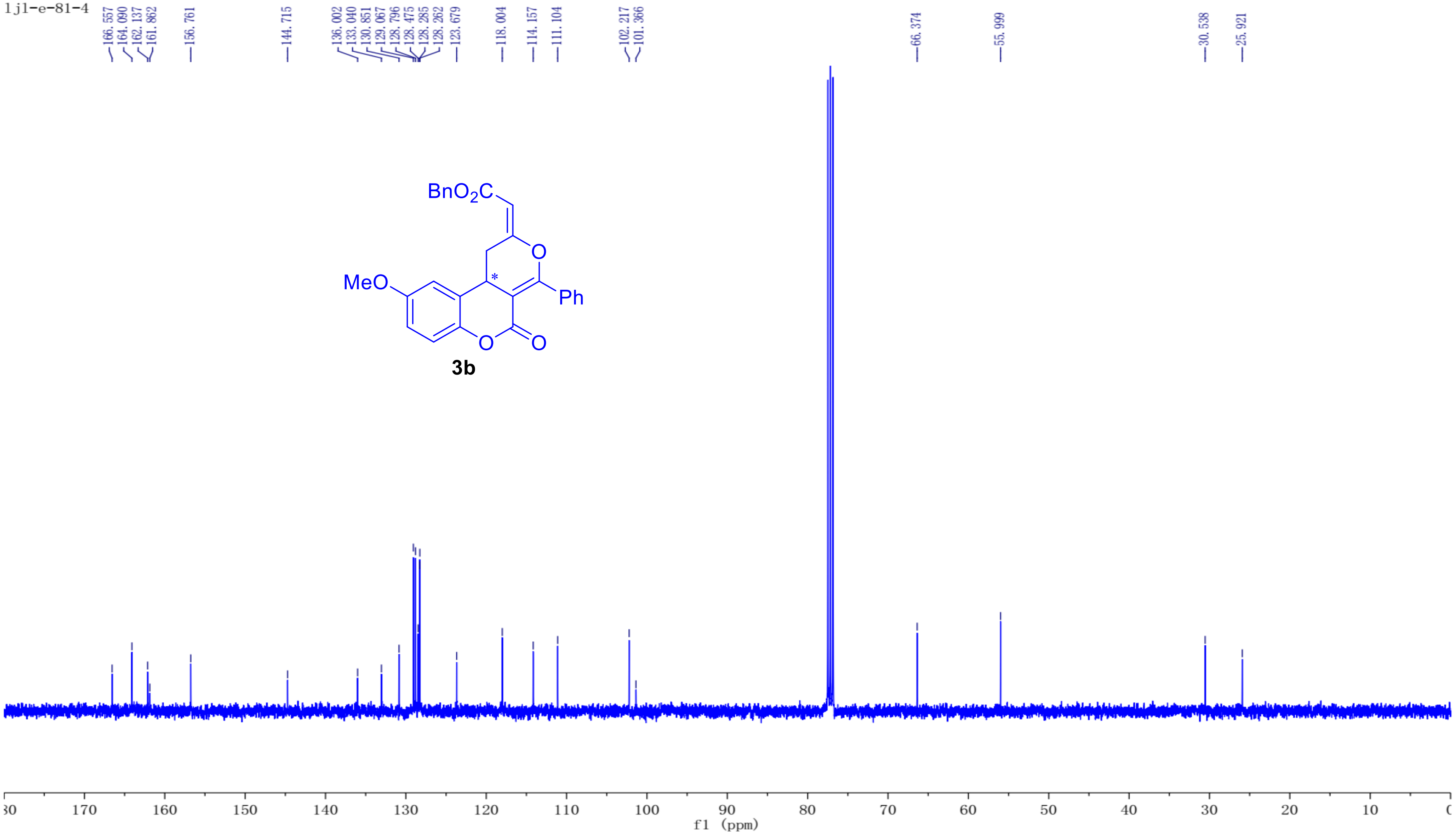

^{1}H NMR Spectrum (400 MHz, Chloroform-*d*) of **3c**

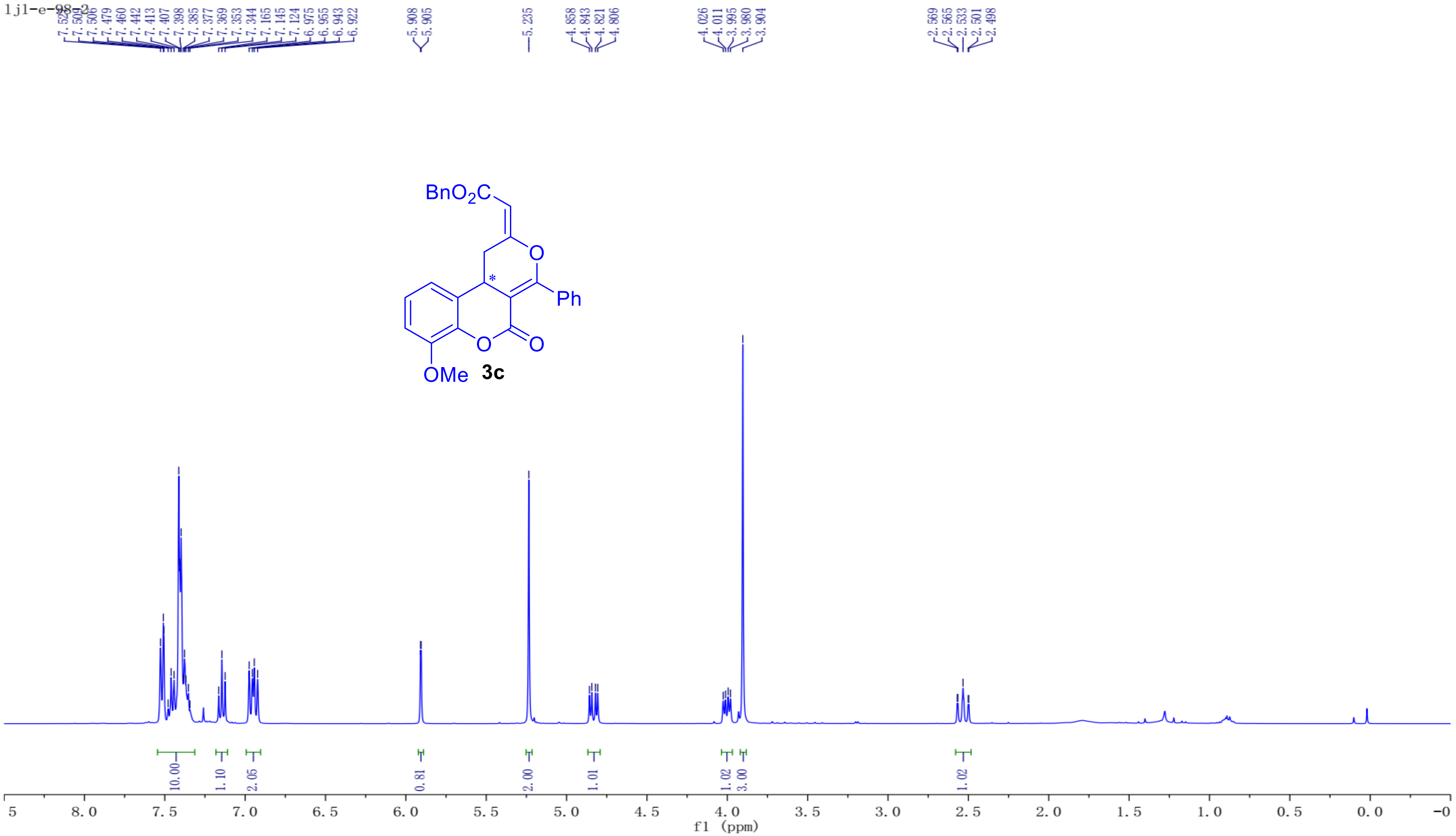

^{13}C {^{1}H} NMR Spectrum (101 MHz, Chloroform-*d*) of **3c**

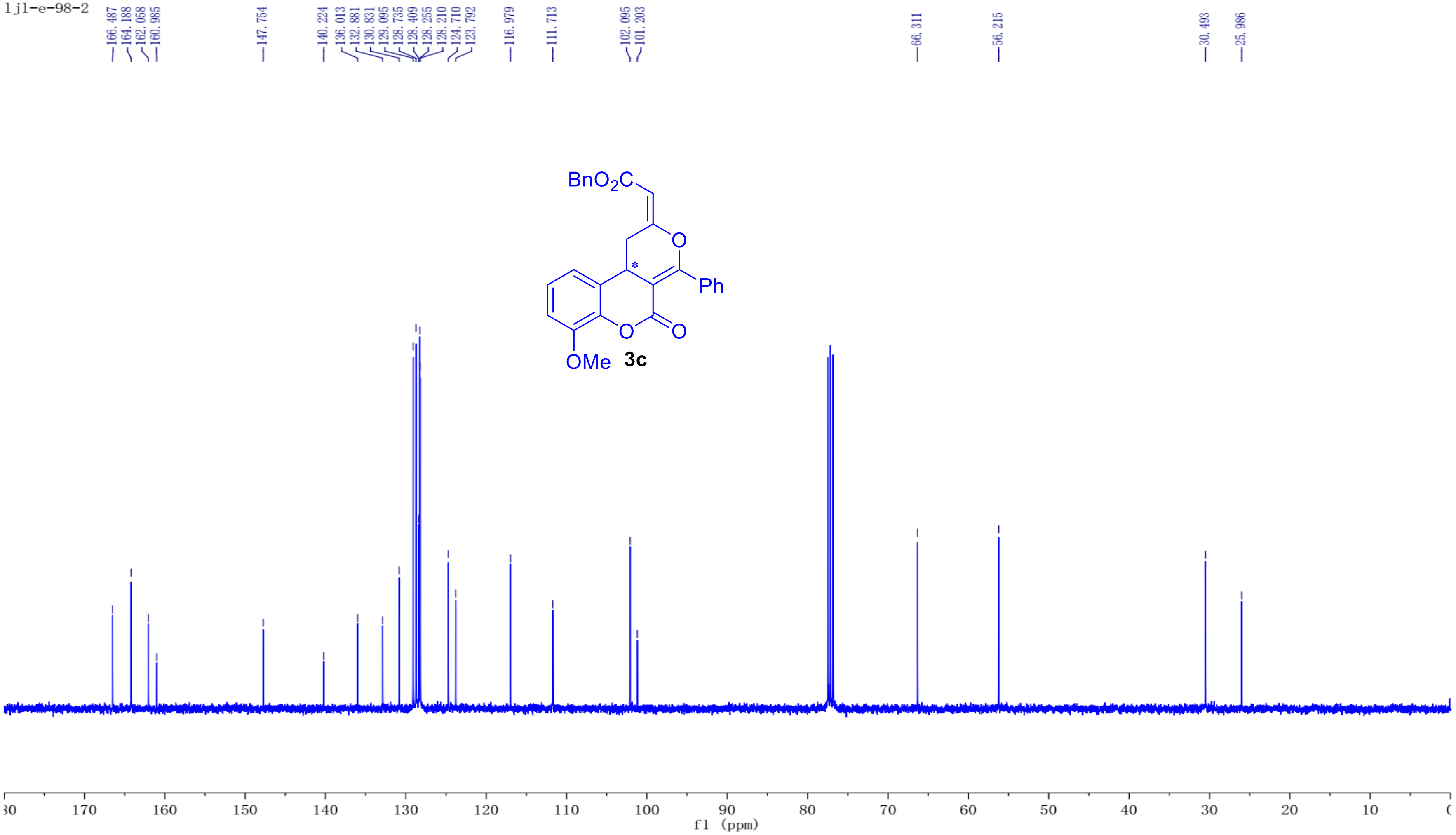

^{1}H NMR Spectrum (400 MHz, Chloroform-*d*) of **3d**

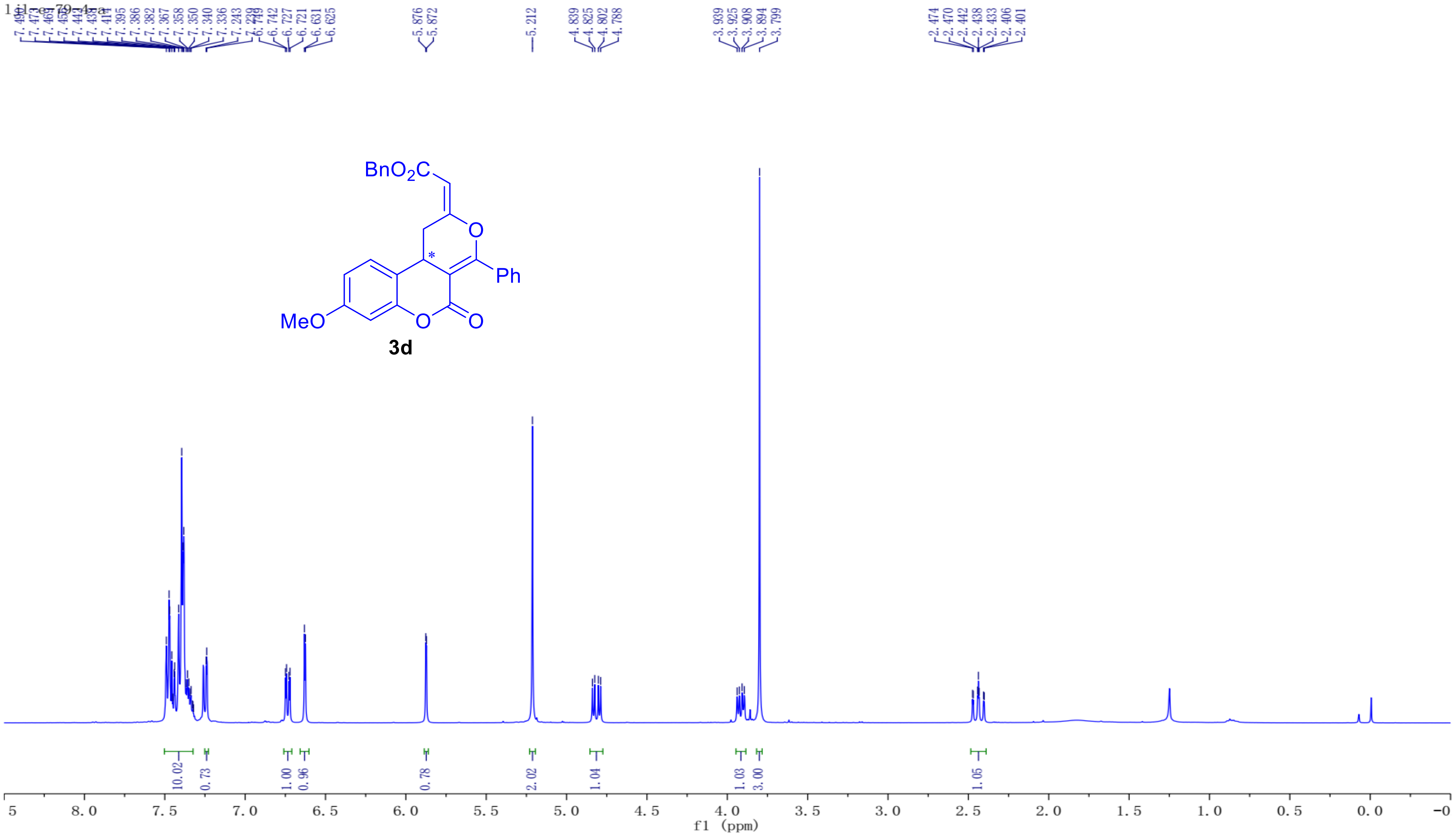

^{13}C {^{1}H} NMR Spectrum (101 MHz, Chloroform-*d*) of **3d**

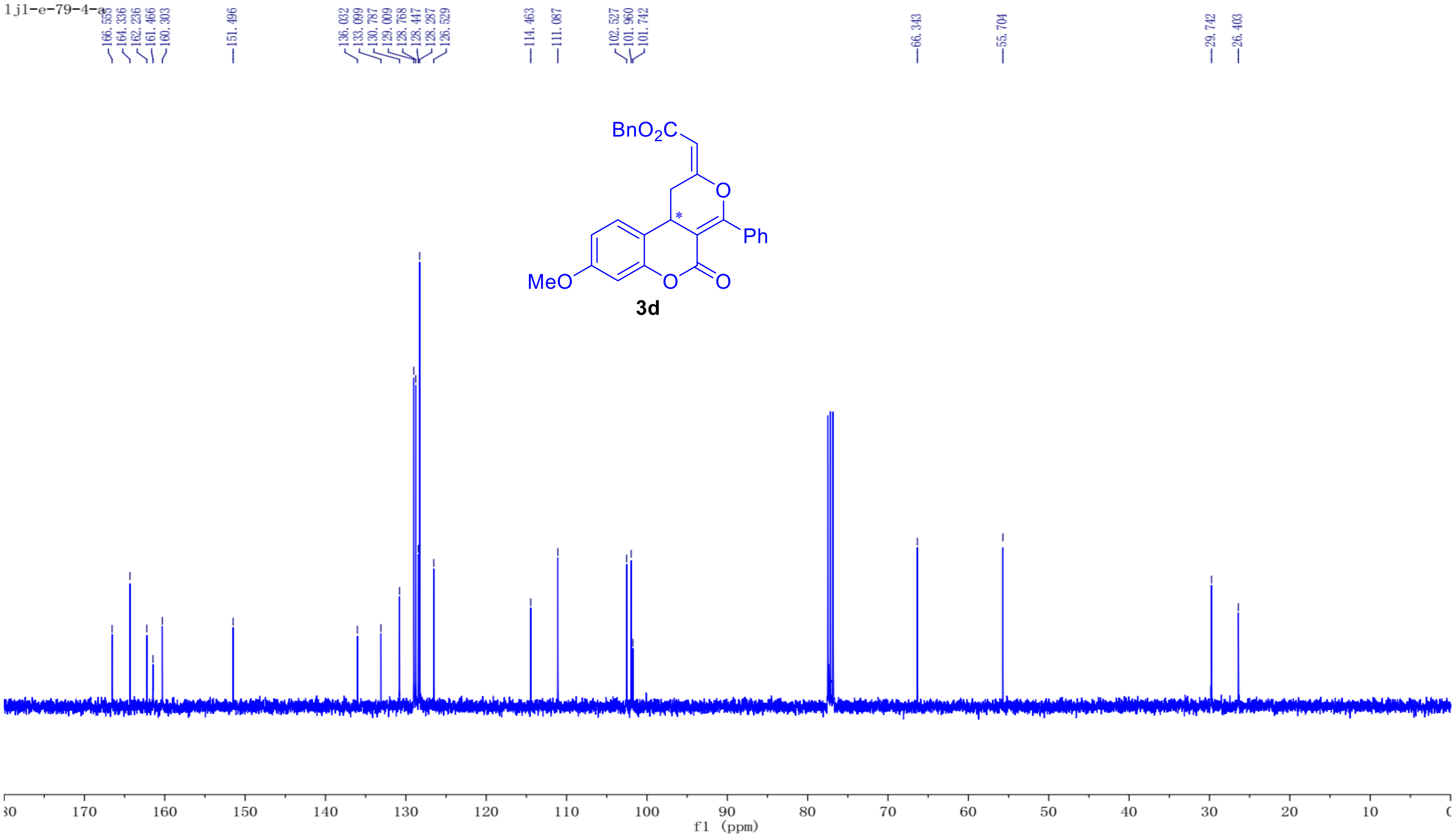

^{1}H NMR Spectrum (400 MHz, Chloroform-*d*) of **3e**

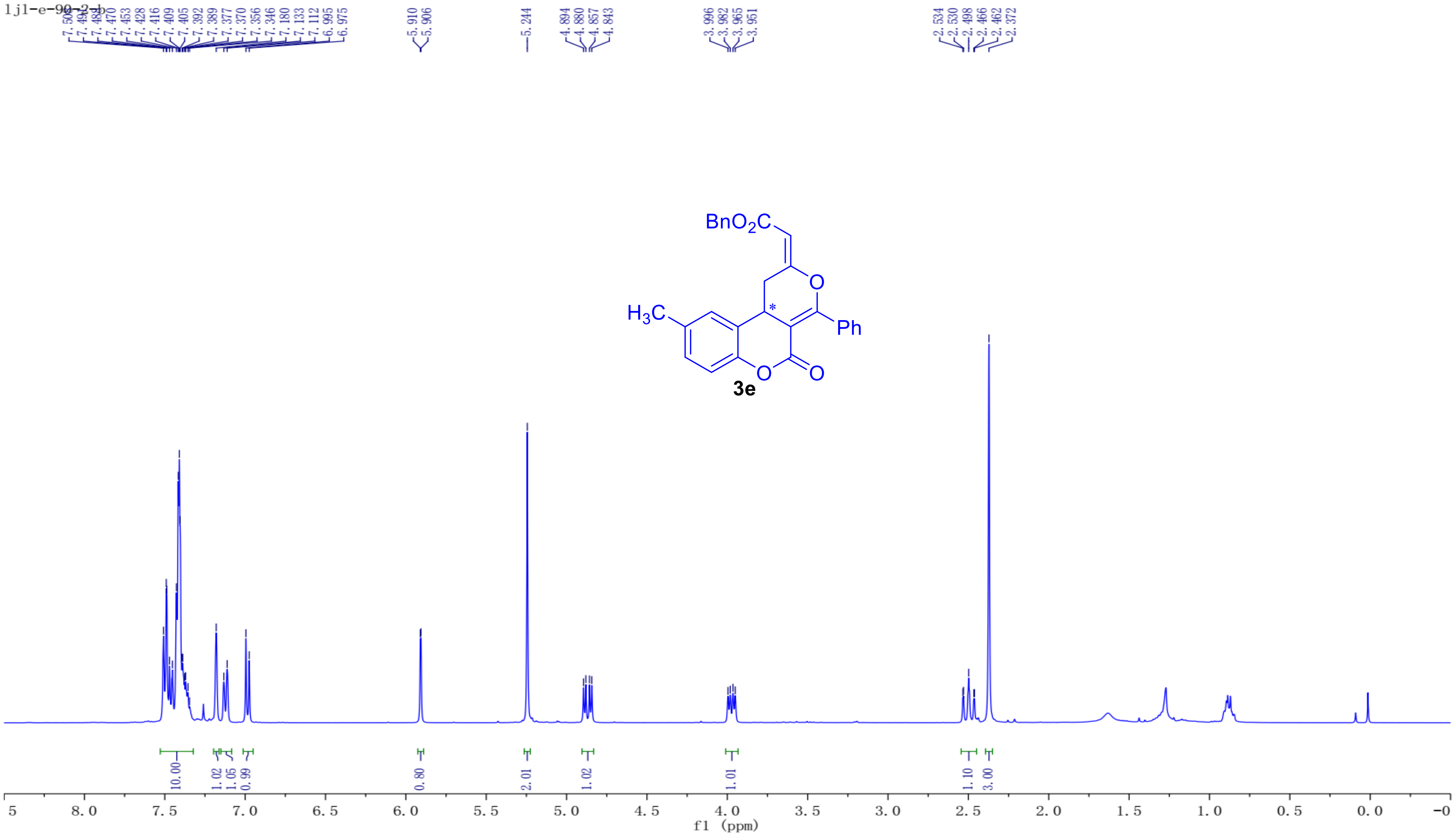

^{13}C {^{1}H} NMR Spectrum (101 MHz, Chloroform-*d*) of **3e**

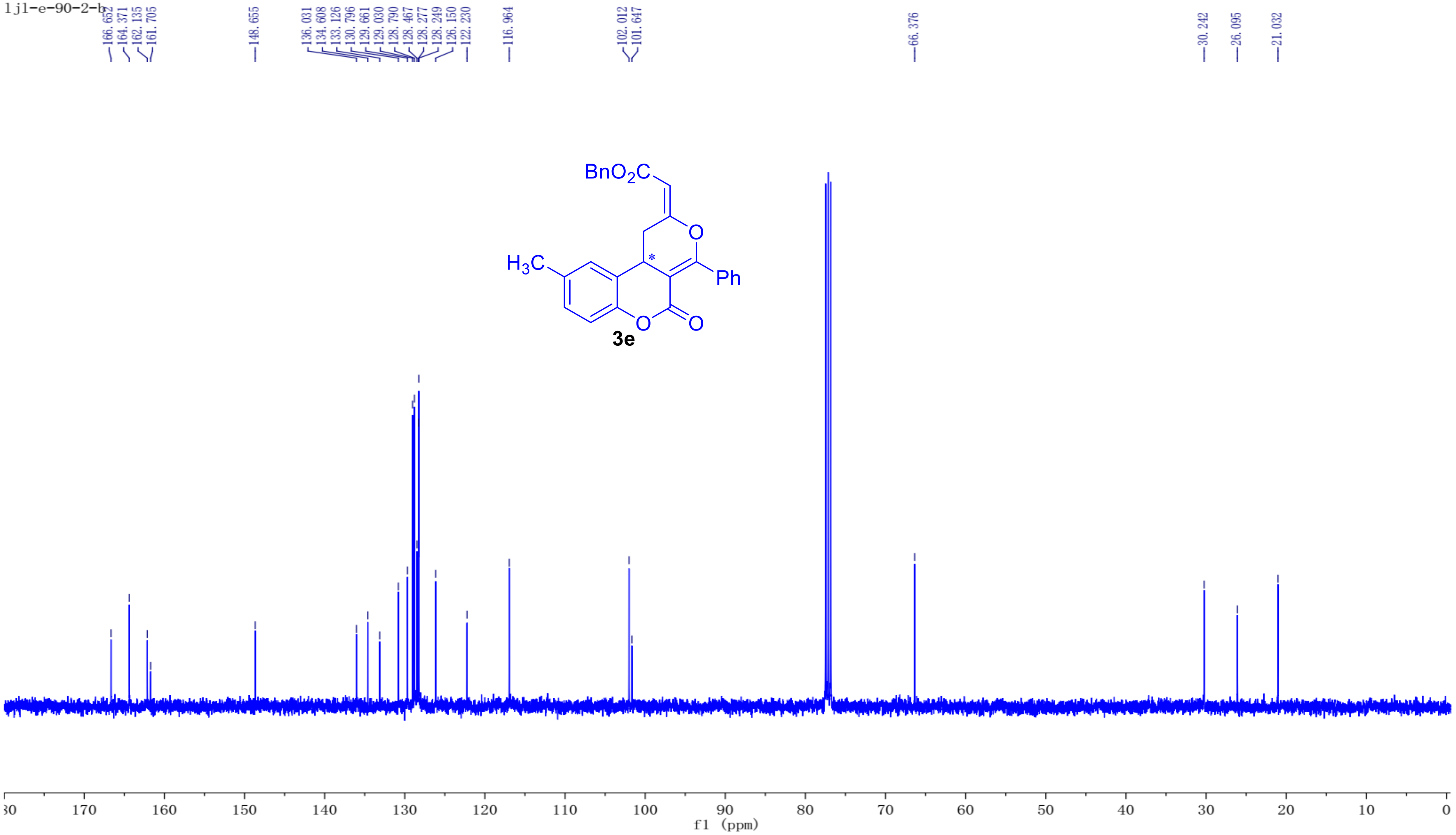

^{1}H NMR Spectrum (400 MHz, Chloroform-*d*) of **3f**

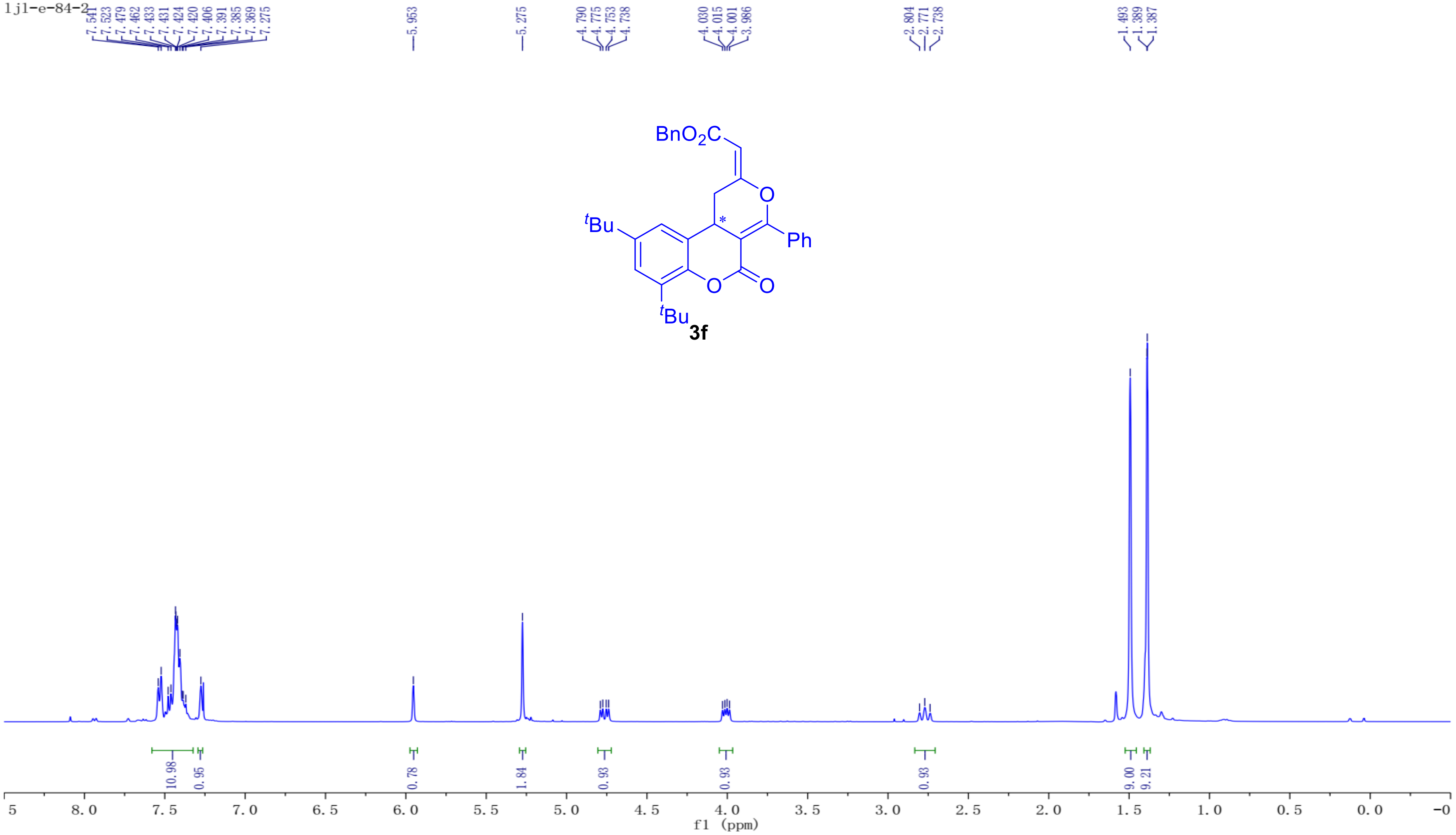

^{13}C {^{1}H} NMR Spectrum (101 MHz, Chloroform-*d*) of **3f**

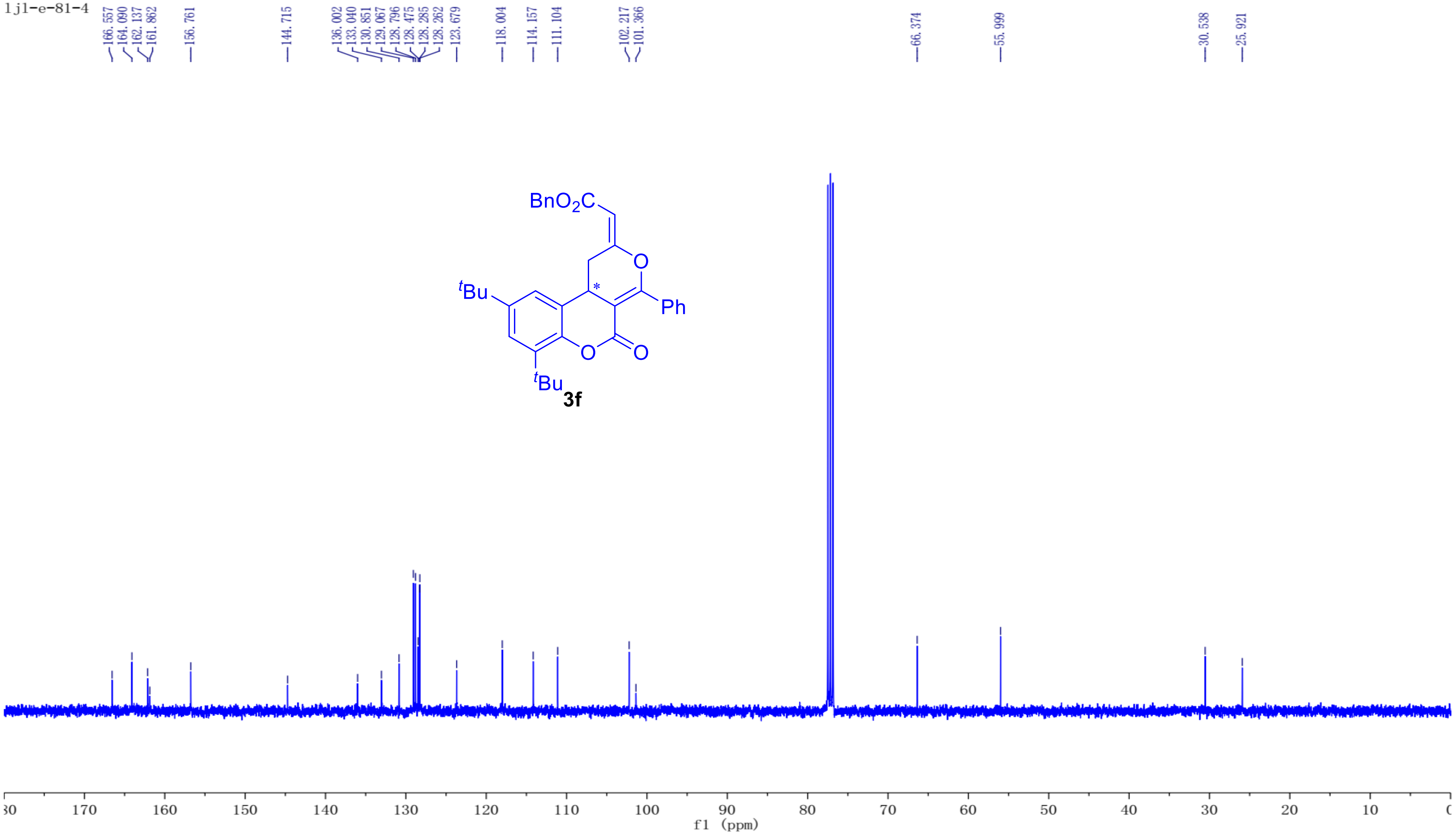

^{1}H NMR Spectrum (400 MHz, Chloroform-*d*) of **3g**

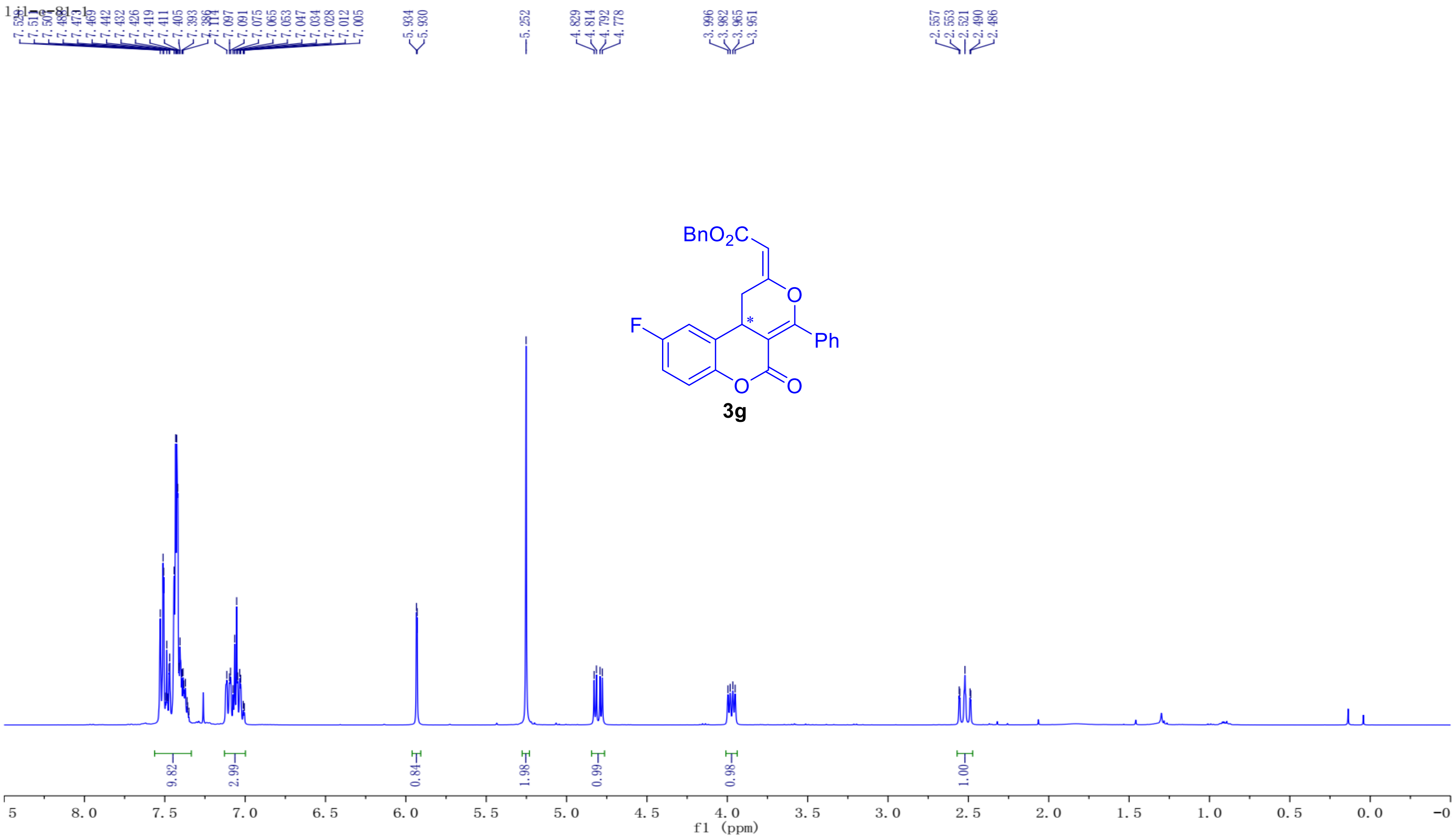

^{13}C {^{1}H} NMR Spectrum (101 MHz, Chloroform-*d*) of **3g**

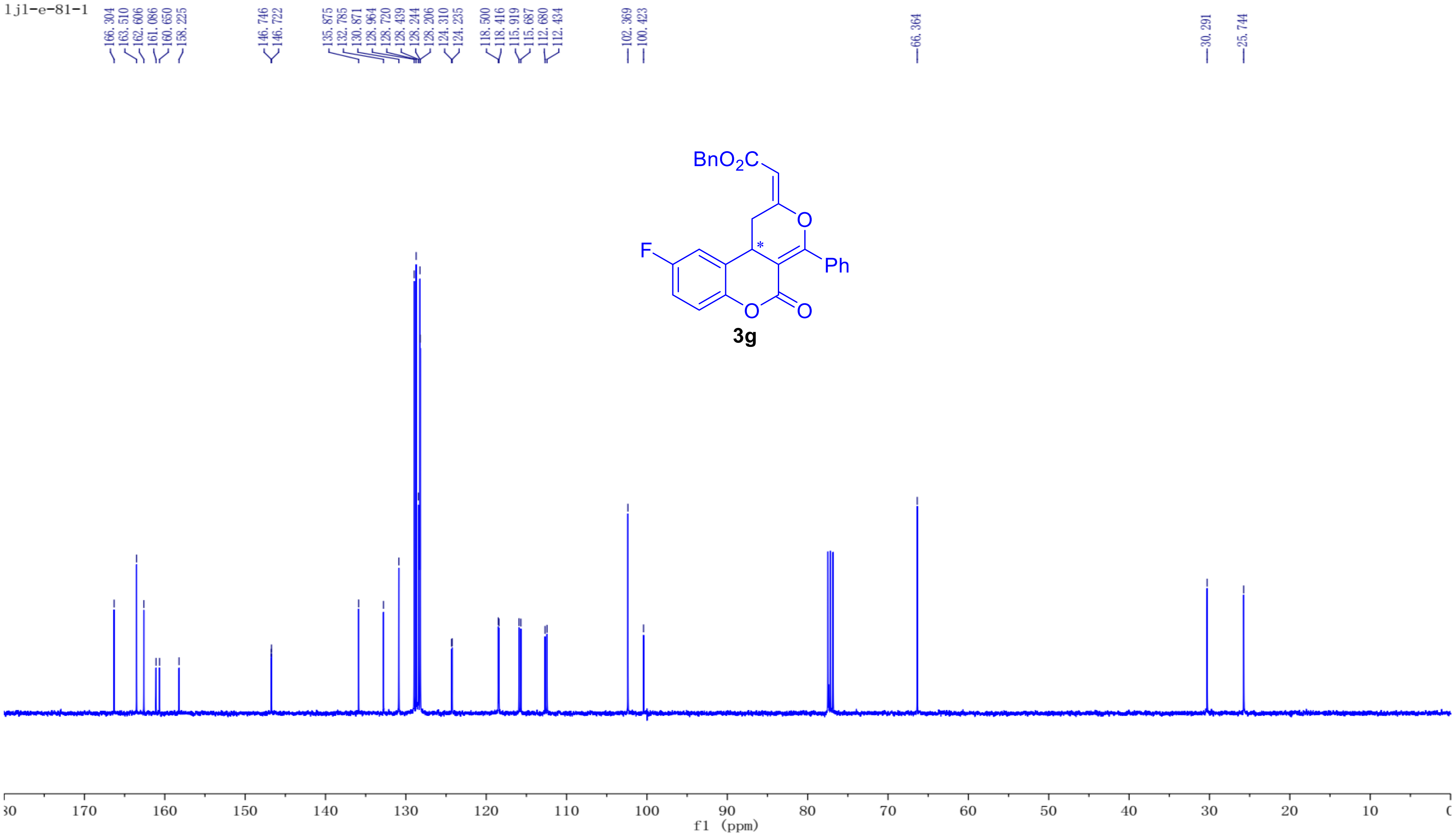

3g

^{1}H NMR Spectrum (400 MHz, Chloroform-*d*) of **3h**

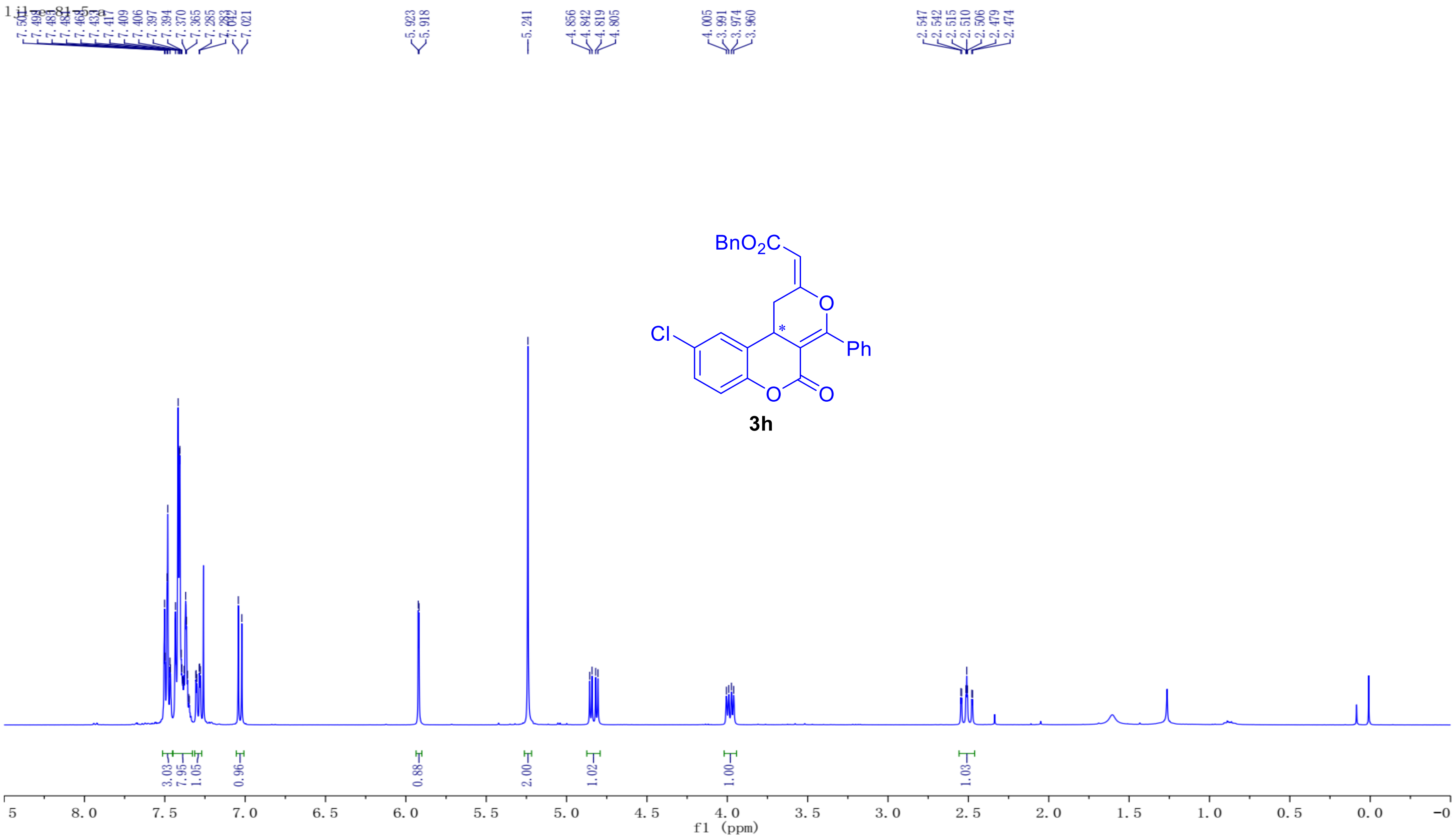

$^{13}C\ \{^{1}H\}$ NMR Spectrum (101 MHz, Chloroform-*d*) of **3h**

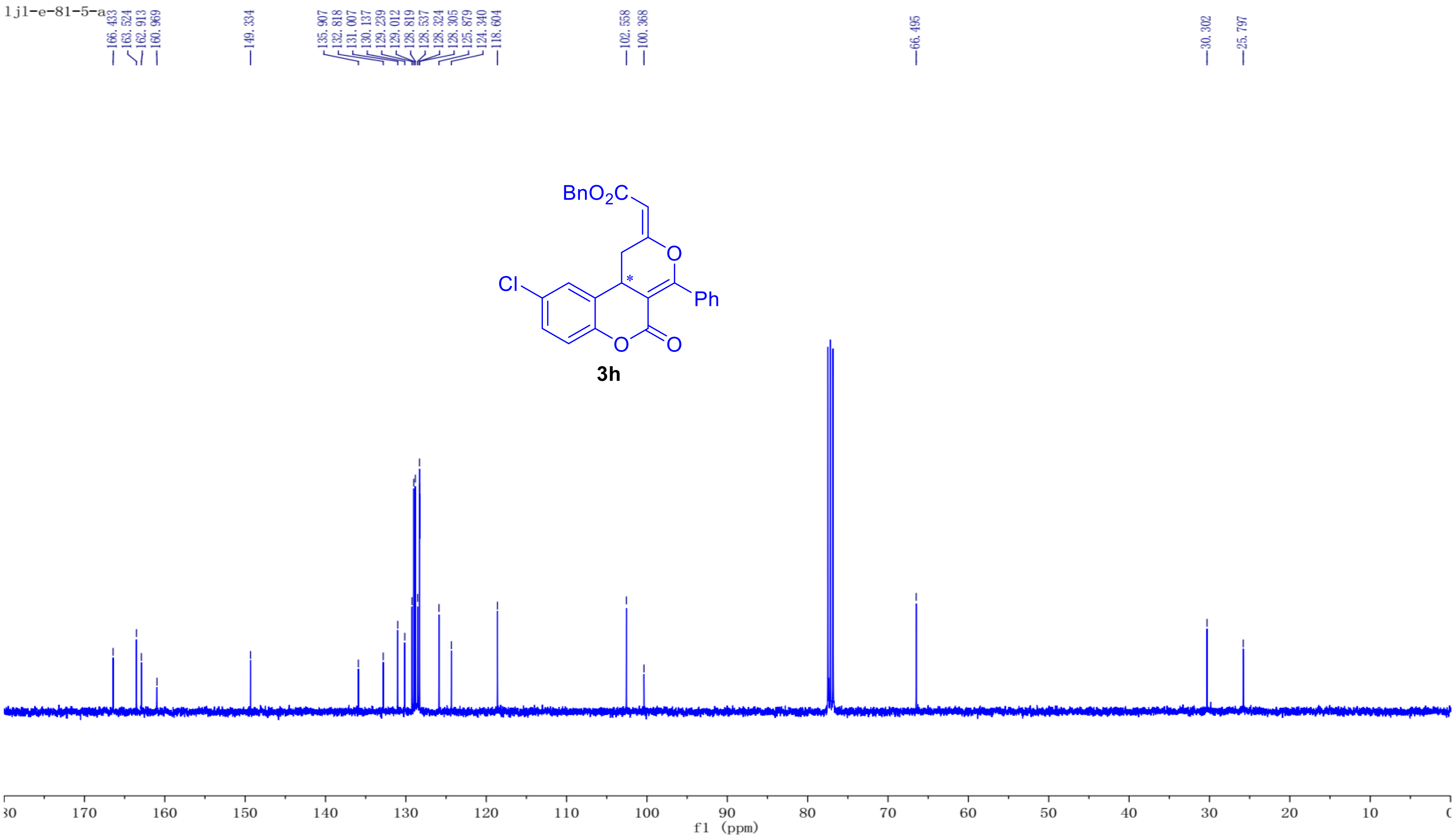

^{1}H NMR Spectrum (400 MHz, Chloroform-*d*) of **3i**

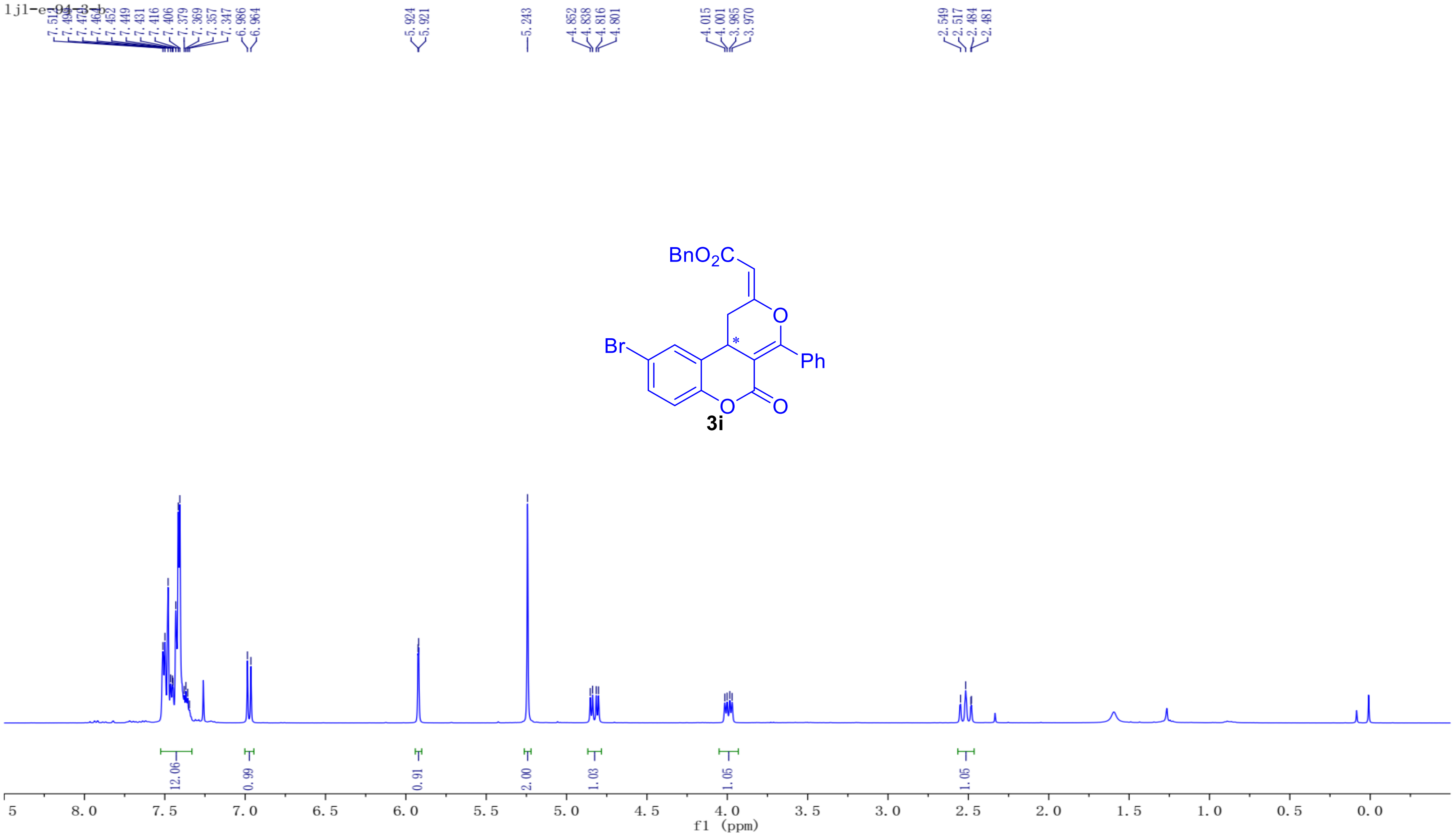

3i

^{13}C {^{1}H} NMR Spectrum (101 MHz, Chloroform-*d*) of **3i**

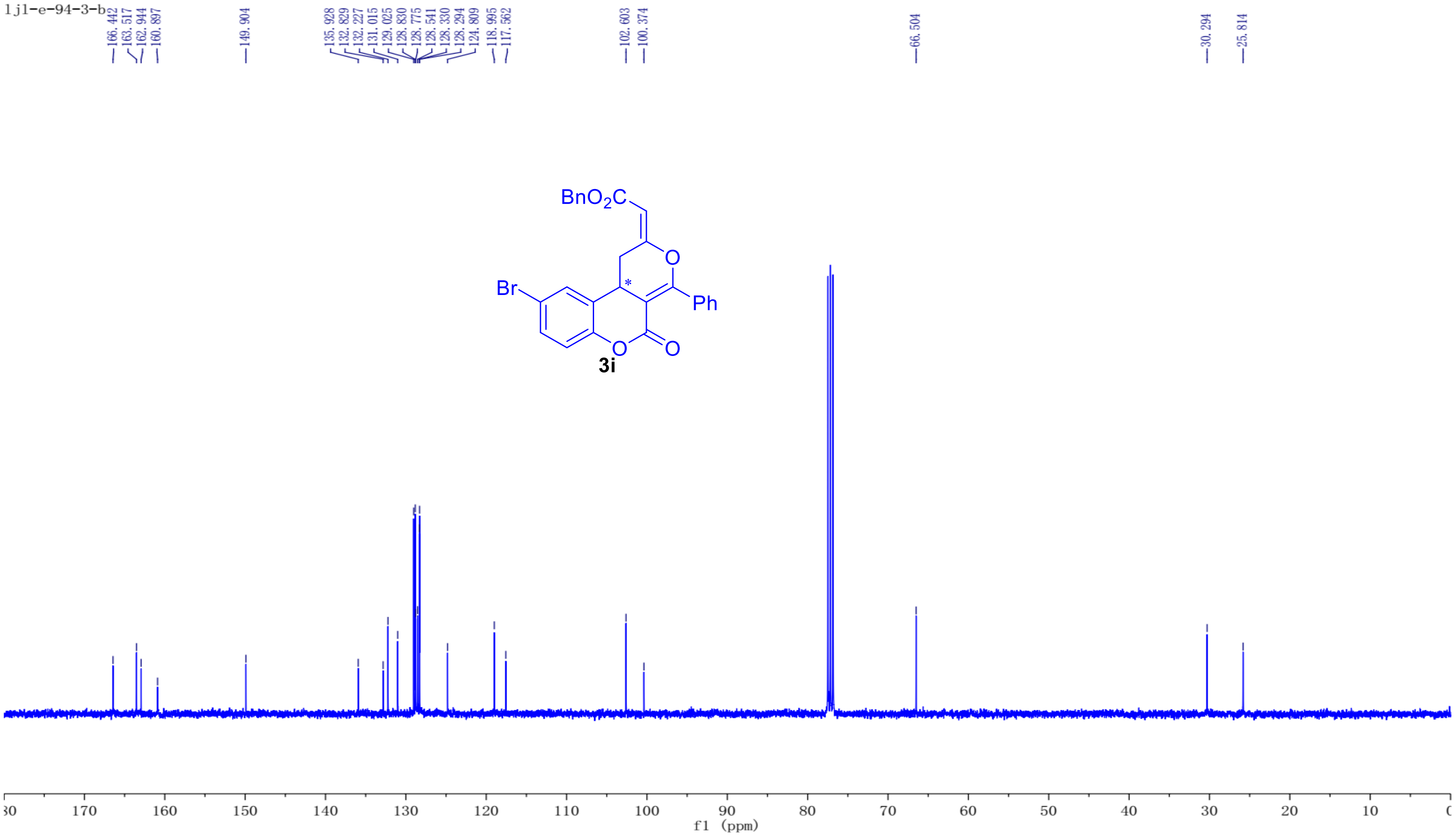
ljl-e-94-3-b
166.442
163.517
162.944
160.897
149.904
135.928
132.829
132.227
131.015
129.025
128.830
128.775
128.541
128.330
128.294
124.809
118.995
117.562
102.603
100.374
66.504
30.294
25.814
BnO2C
O
Br
Ph
O
O
3i
f1 (ppm)

^{1}H NMR Spectrum (400 MHz, Chloroform-*d*) of **3j**

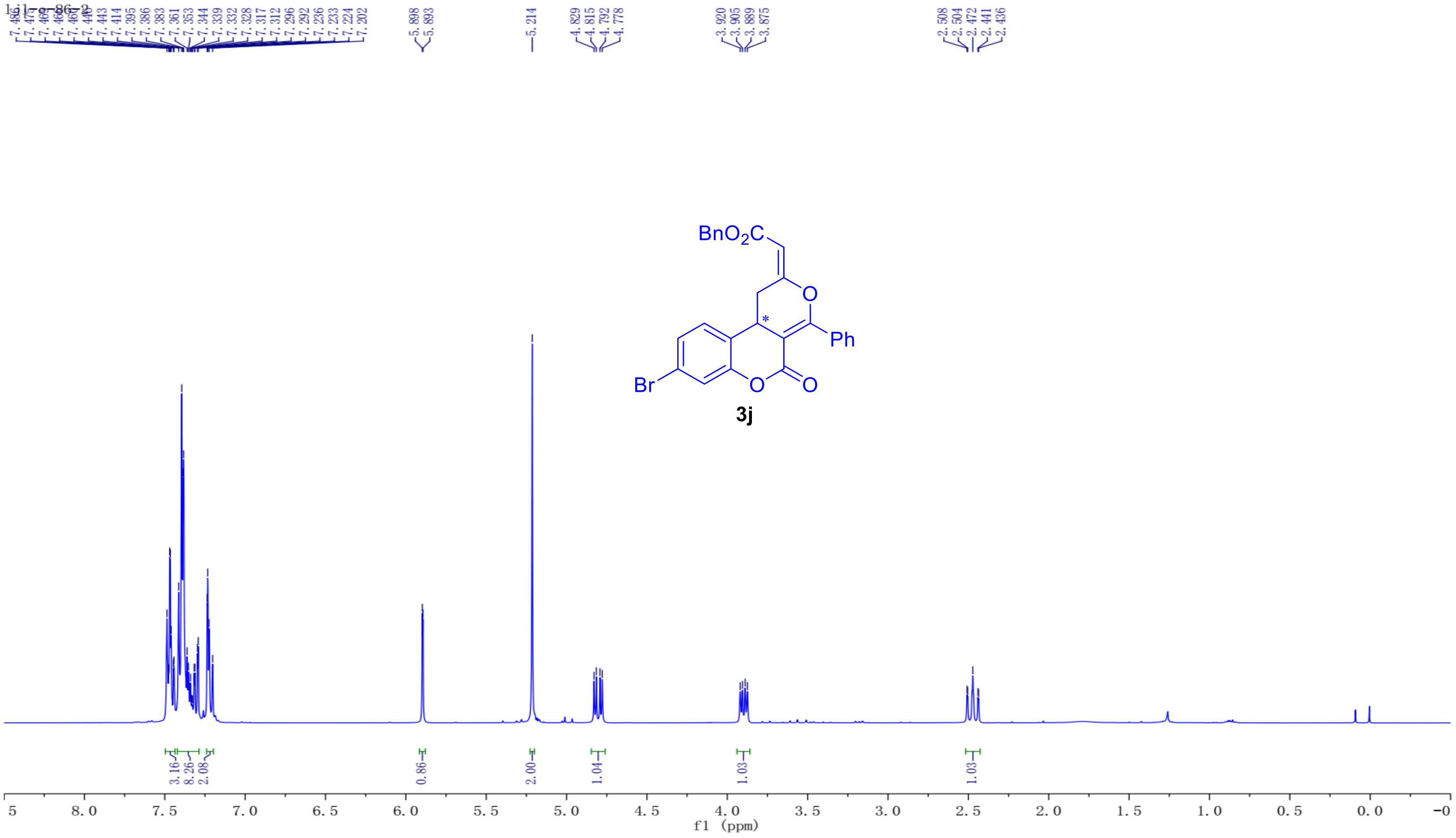

$^{13}C\ \{^1H\}$ NMR Spectrum (101 MHz, Chloroform-*d*) of **3j**

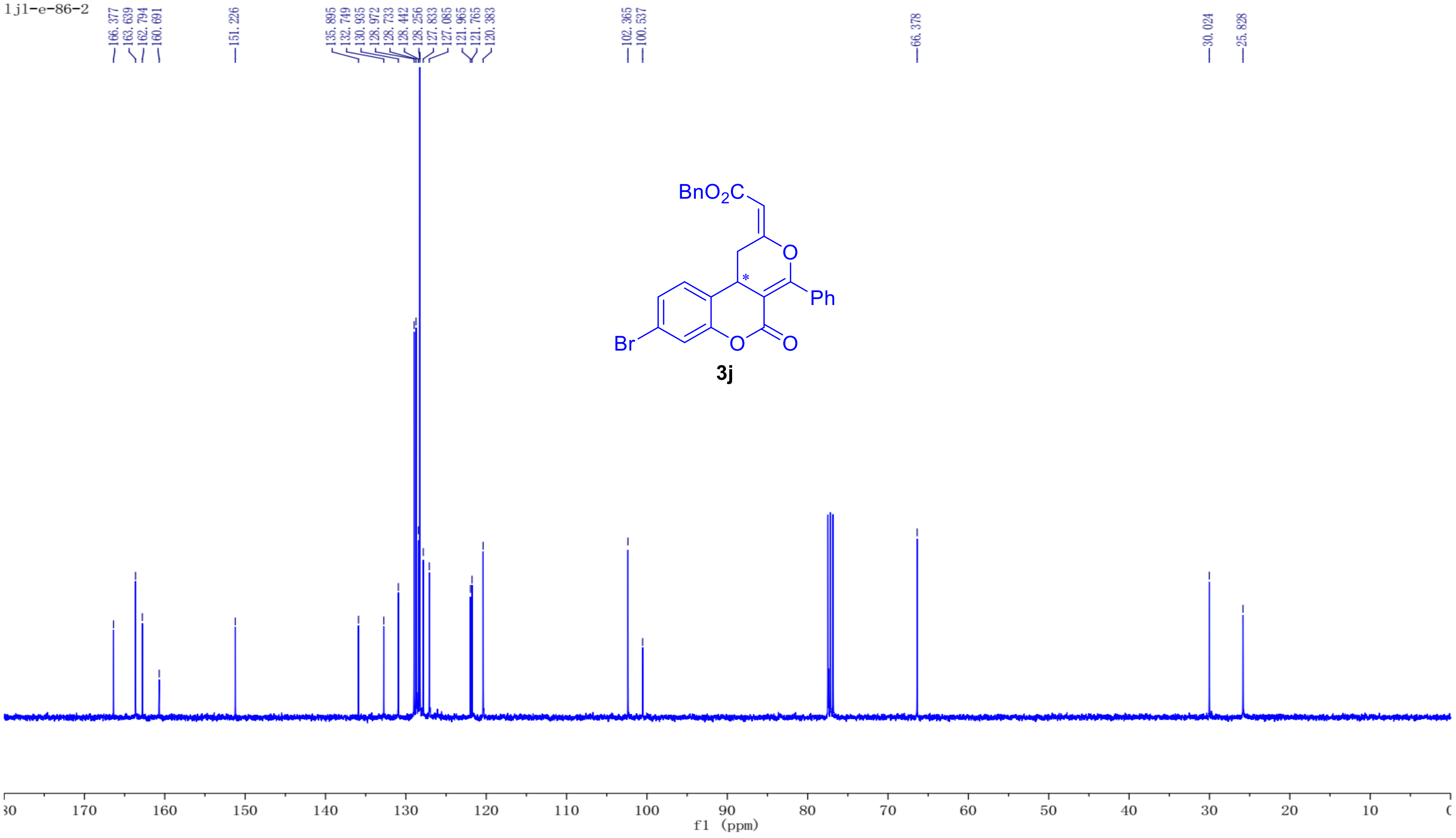

^{1}H NMR Spectrum (400 MHz, Chloroform-*d*) of **3k**

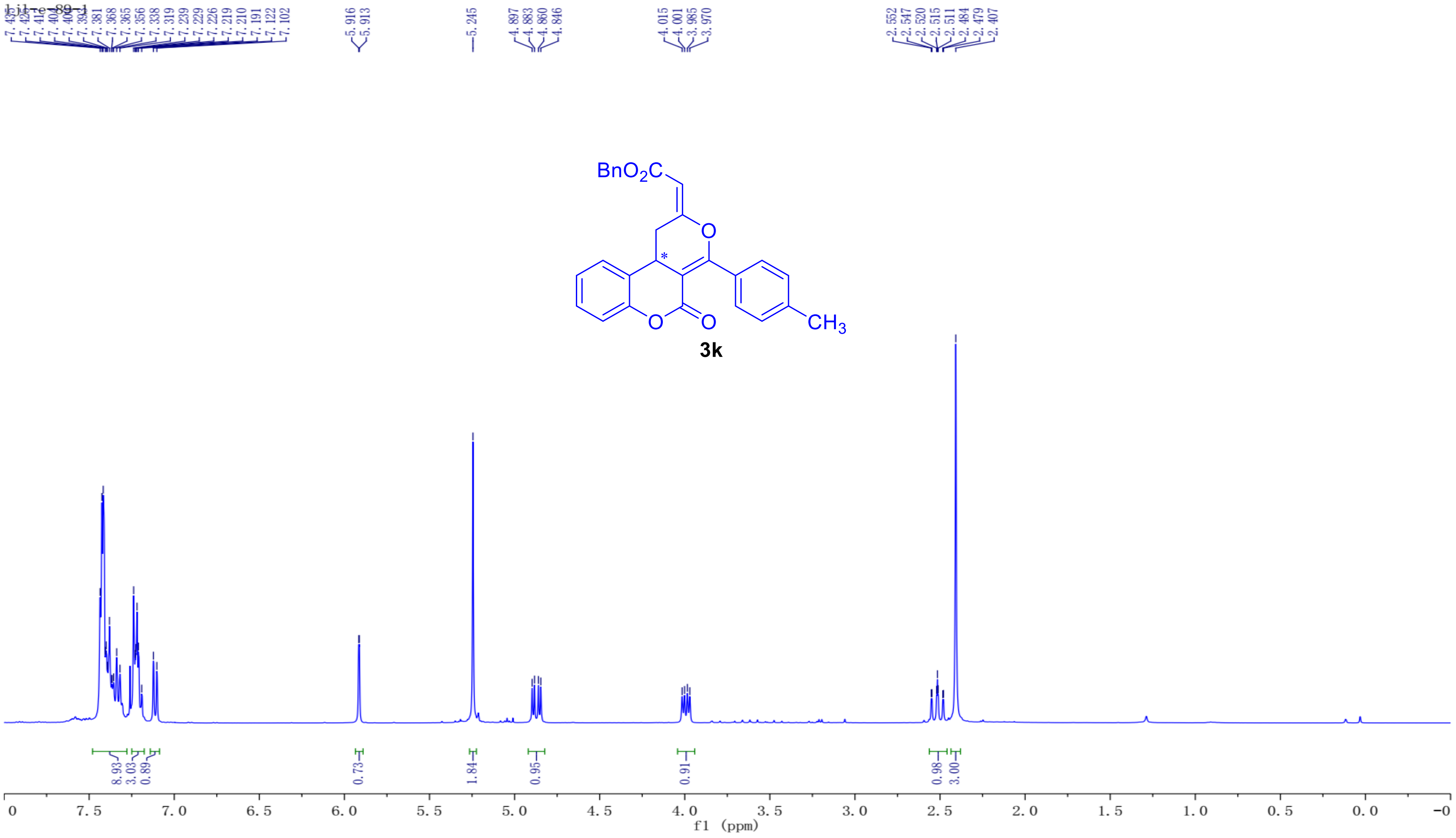

^{13}C {^{1}H} NMR Spectrum (101 MHz, Chloroform-*d*) of **3k**

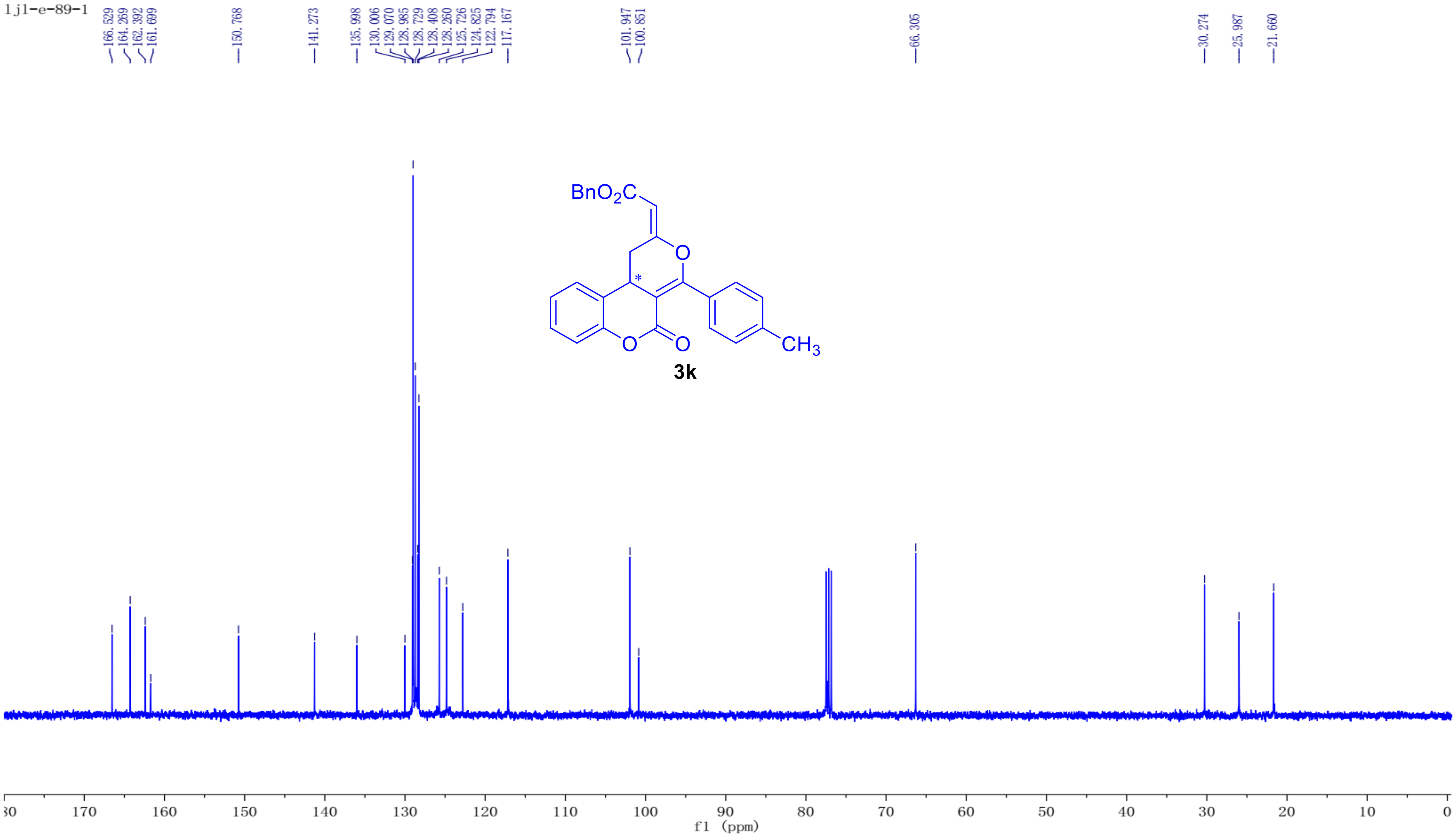
1jl-e-89-1
166.529
164.269
162.392
161.699
150.768
141.273
135.998
130.006
129.070
128.985
128.729
128.408
128.260
125.726
124.825
122.794
117.167
101.947
100.851
66.305
30.274
25.987
21.660
BnO2C
O
O
O
CH3
3k
170
160
150
140
130
120
110
100
90
80
70
60
50
40
30
20
10
0
f1 (ppm)

^{1}H NMR Spectrum (400 MHz, Chloroform-*d*) of **3i**

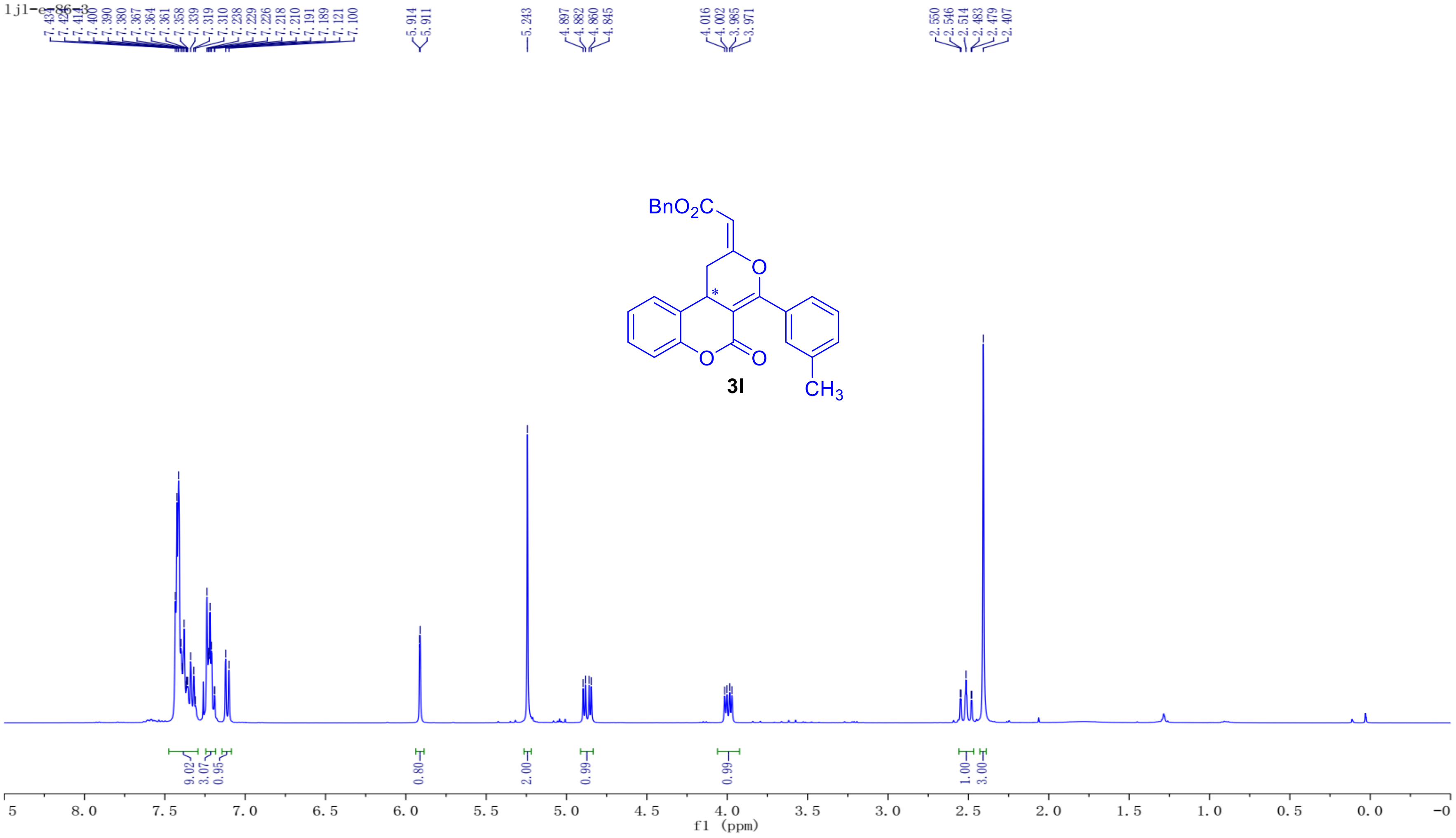

^{13}C {^{1}H} NMR Spectrum (101 MHz, Chloroform-*d*) of **3i**

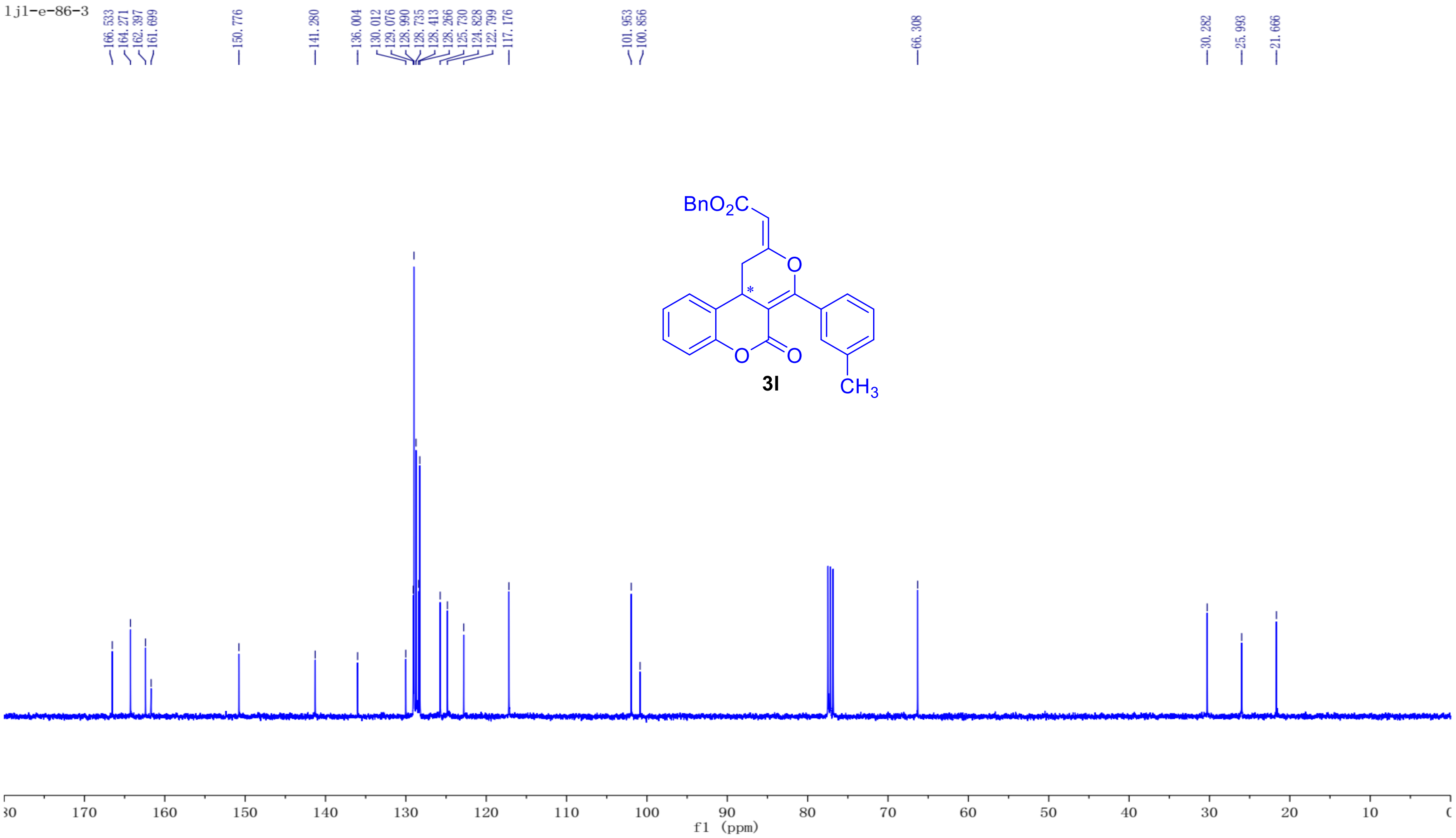

^{1}H NMR Spectrum (400 MHz, Chloroform-*d*) of **3m**

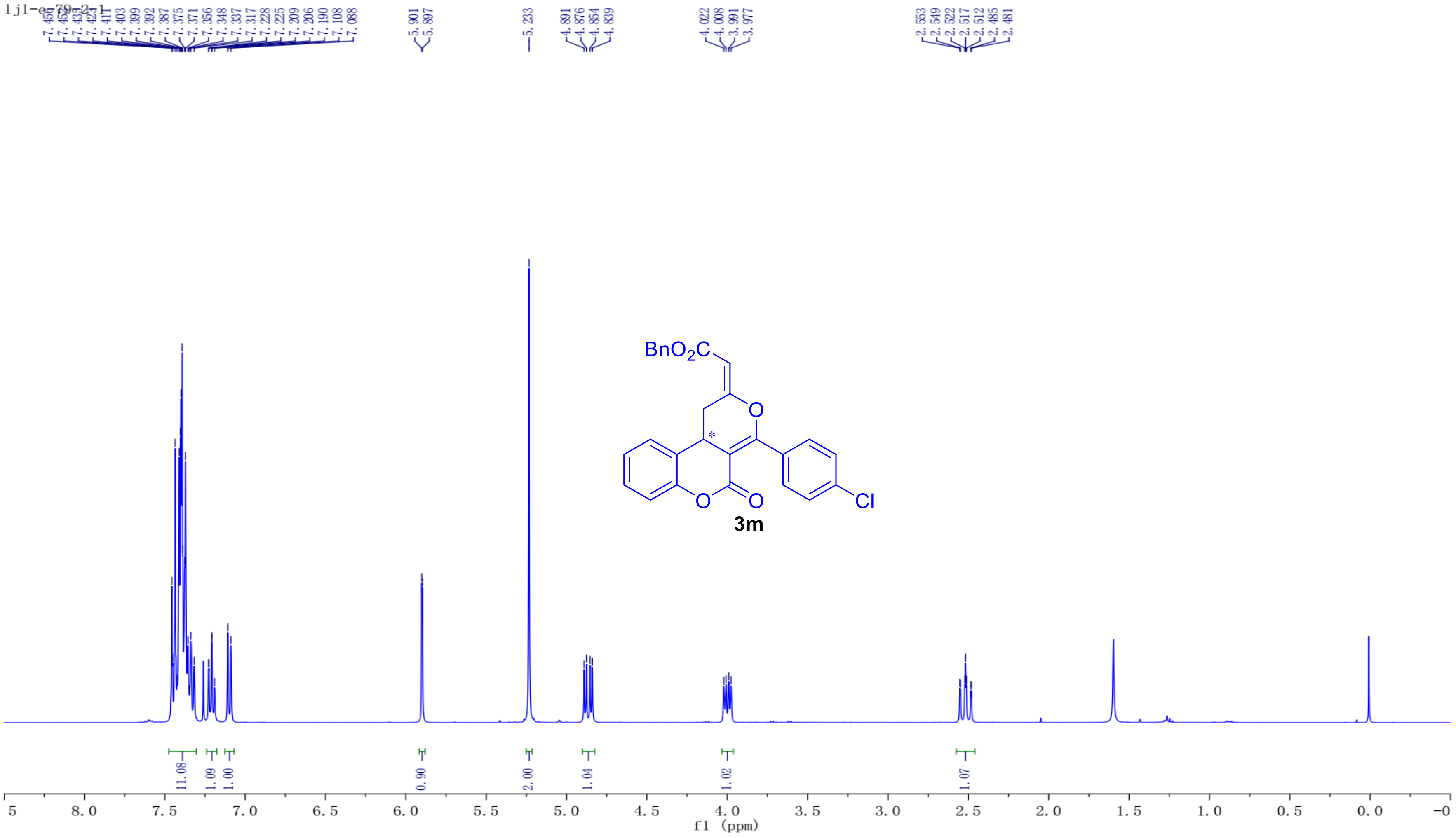

^{13}C {^{1}H} NMR Spectrum (101 MHz, Chloroform-*d*) of **3m**

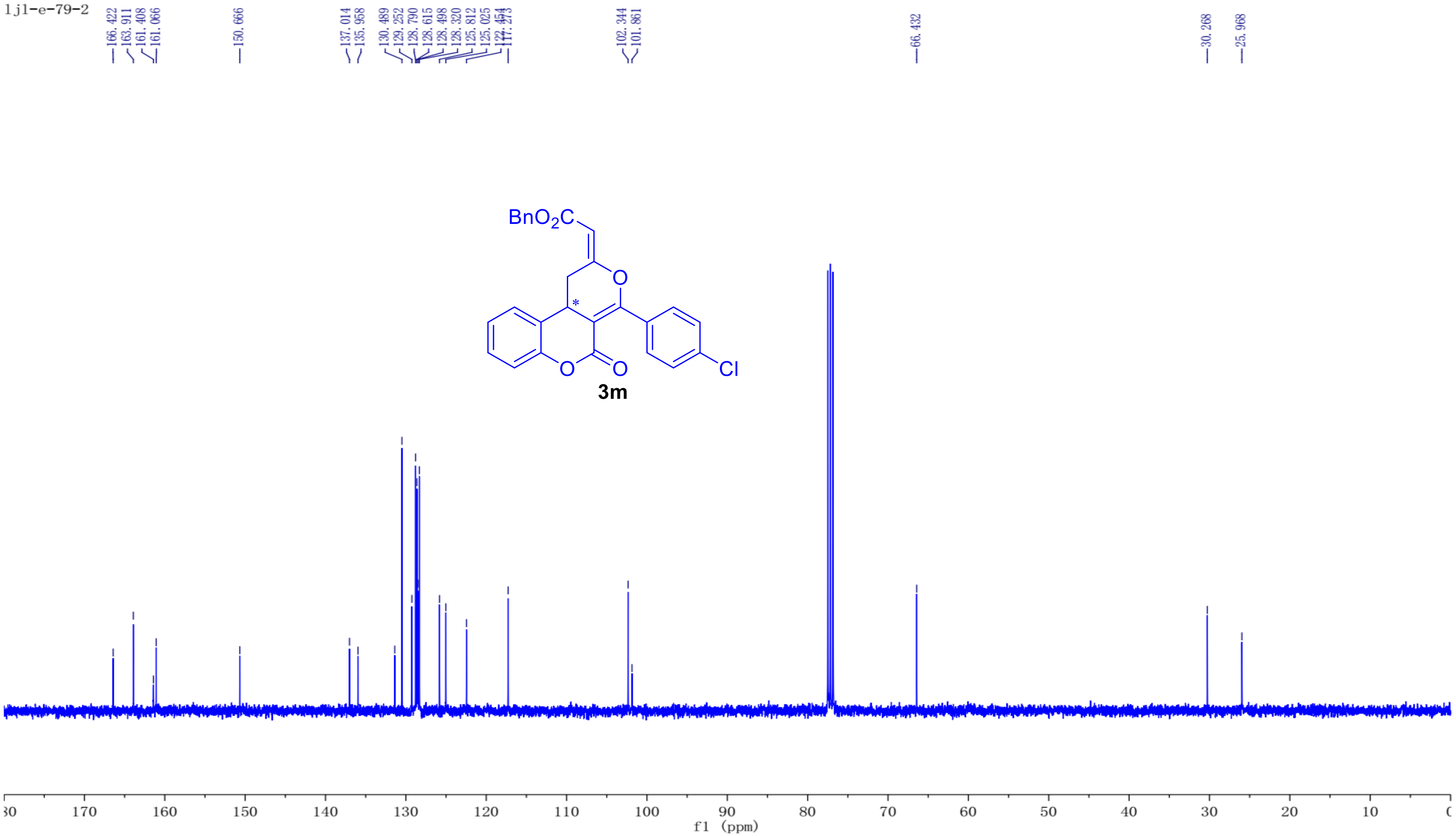

^{1}H NMR Spectrum (400 MHz, Chloroform-*d*) of **3n**

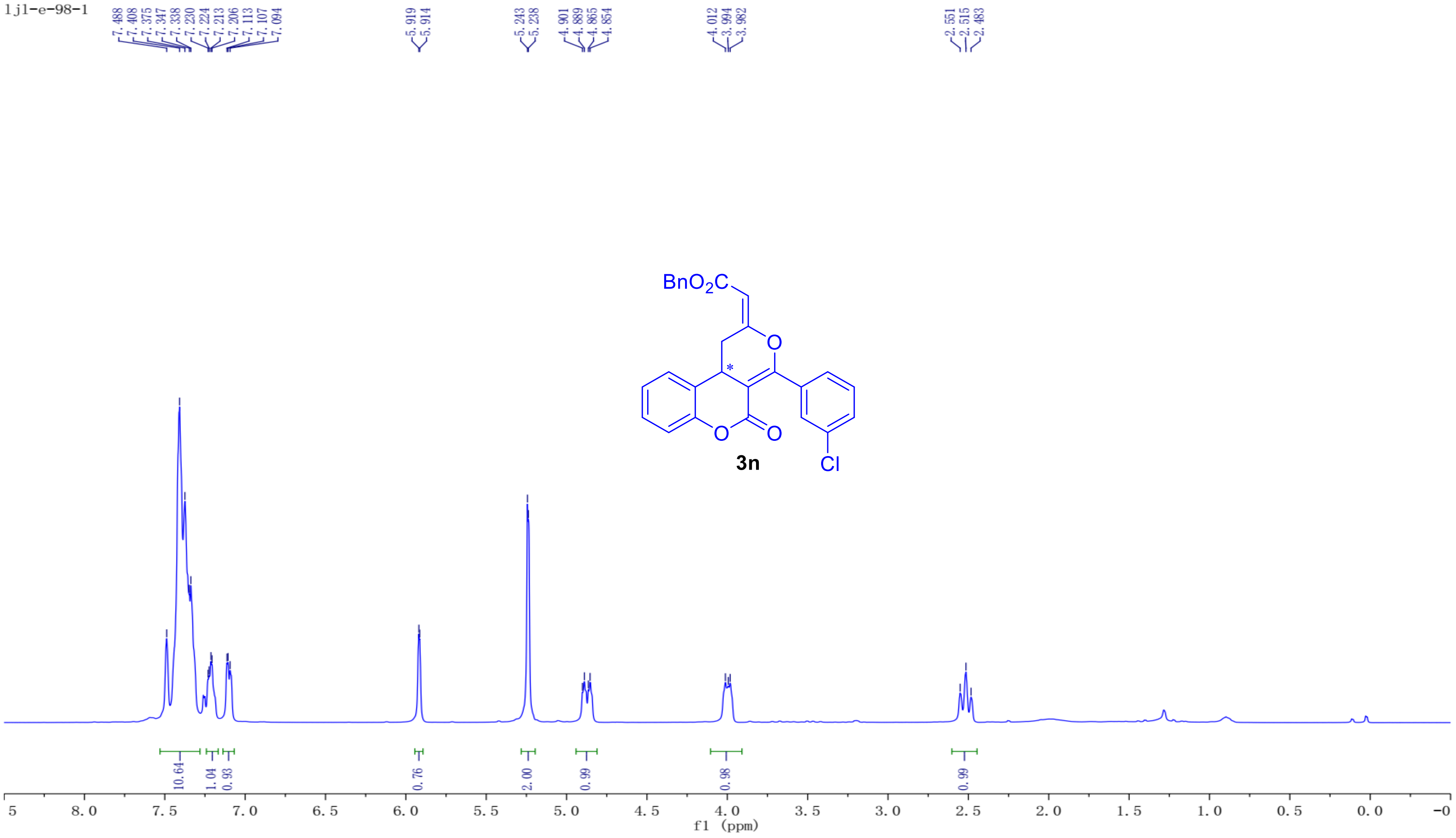

$^{13}C\ \{^{1}H\}$ NMR Spectrum (101 MHz, Chloroform-*d*) of **3n**

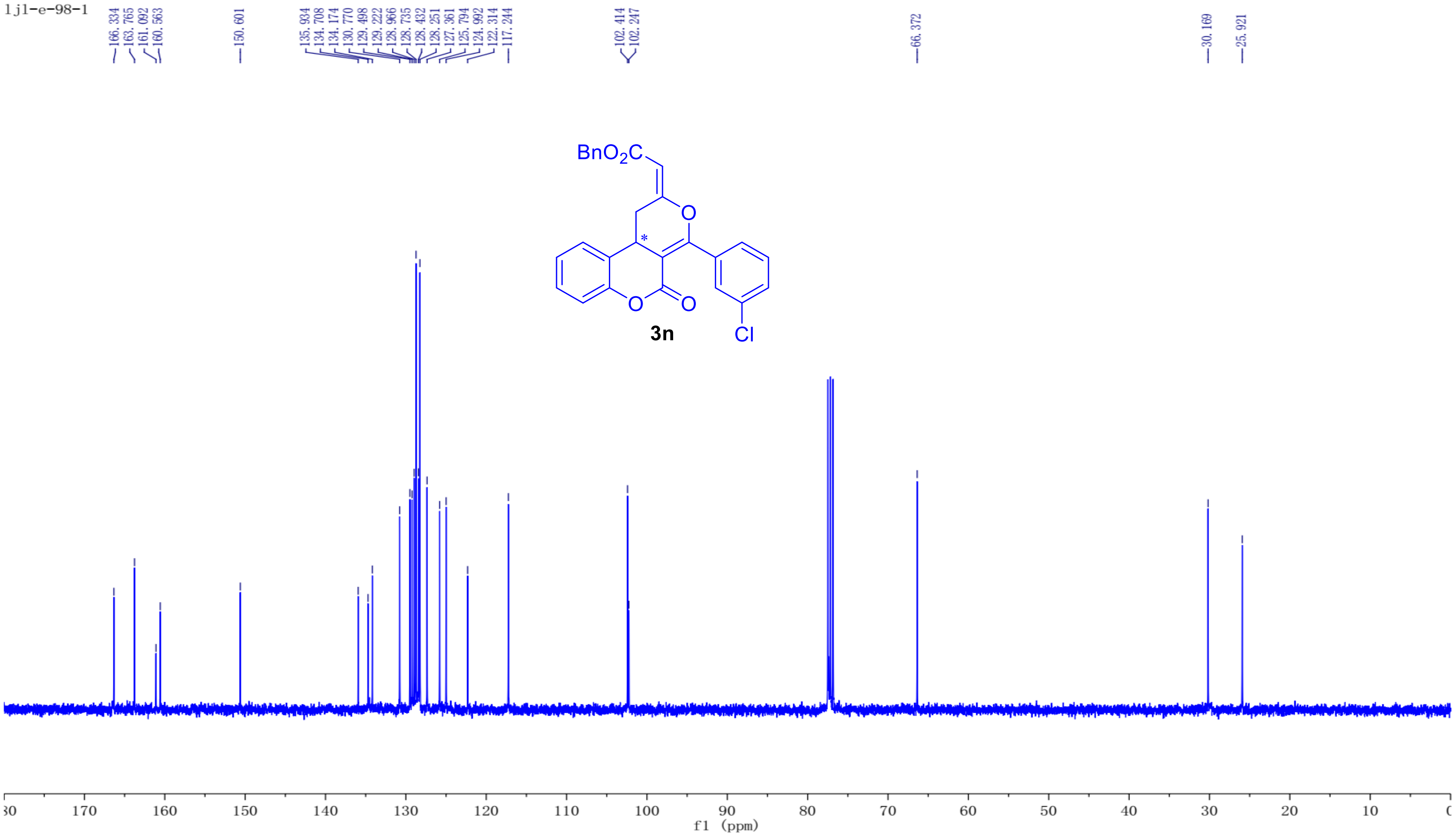

^{1}H NMR Spectrum (400 MHz, Chloroform-*d*) of **3o**

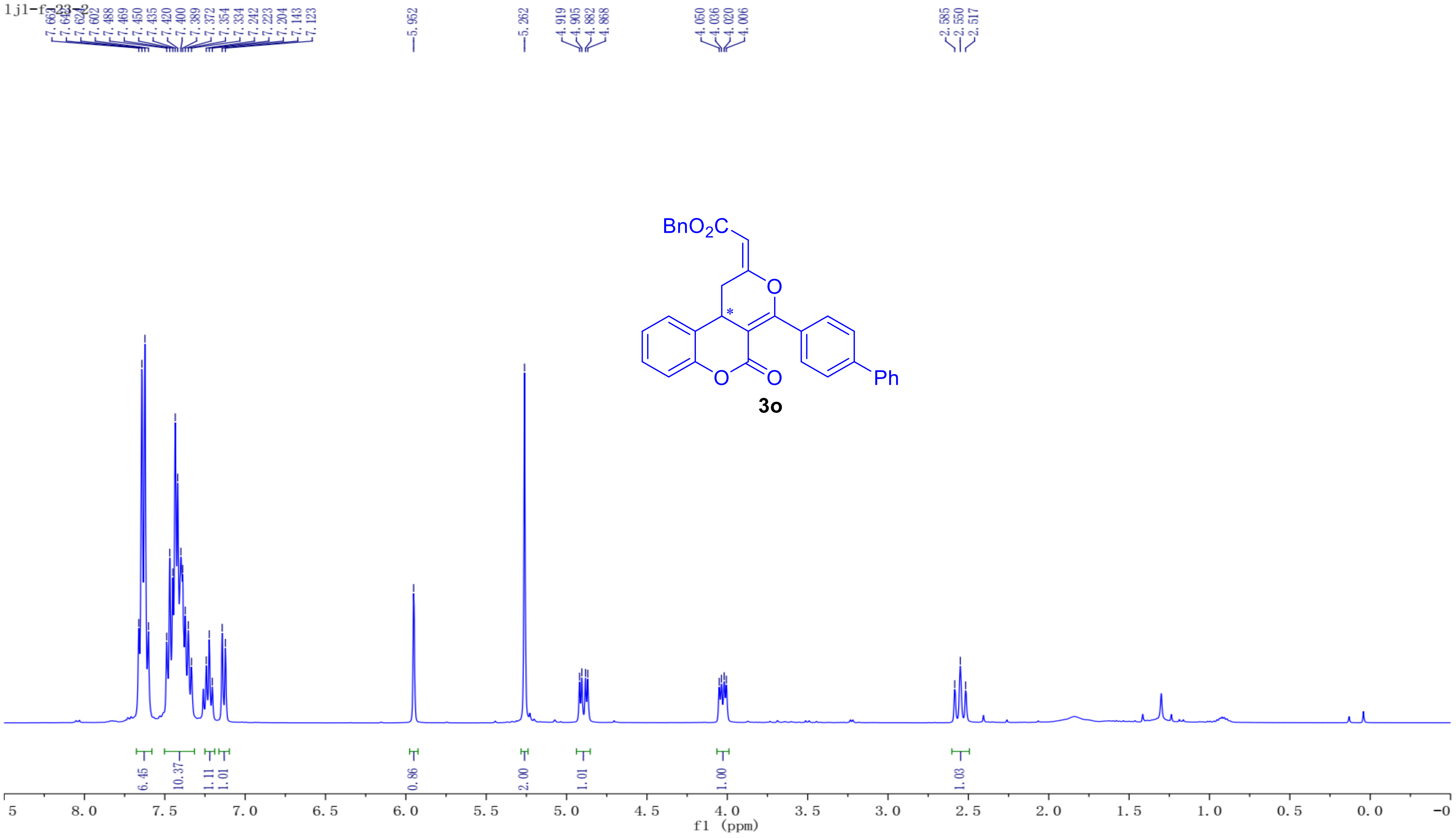

^{13}C {^{1}H} NMR Spectrum (101 MHz, Chloroform-*d*) of **3o**

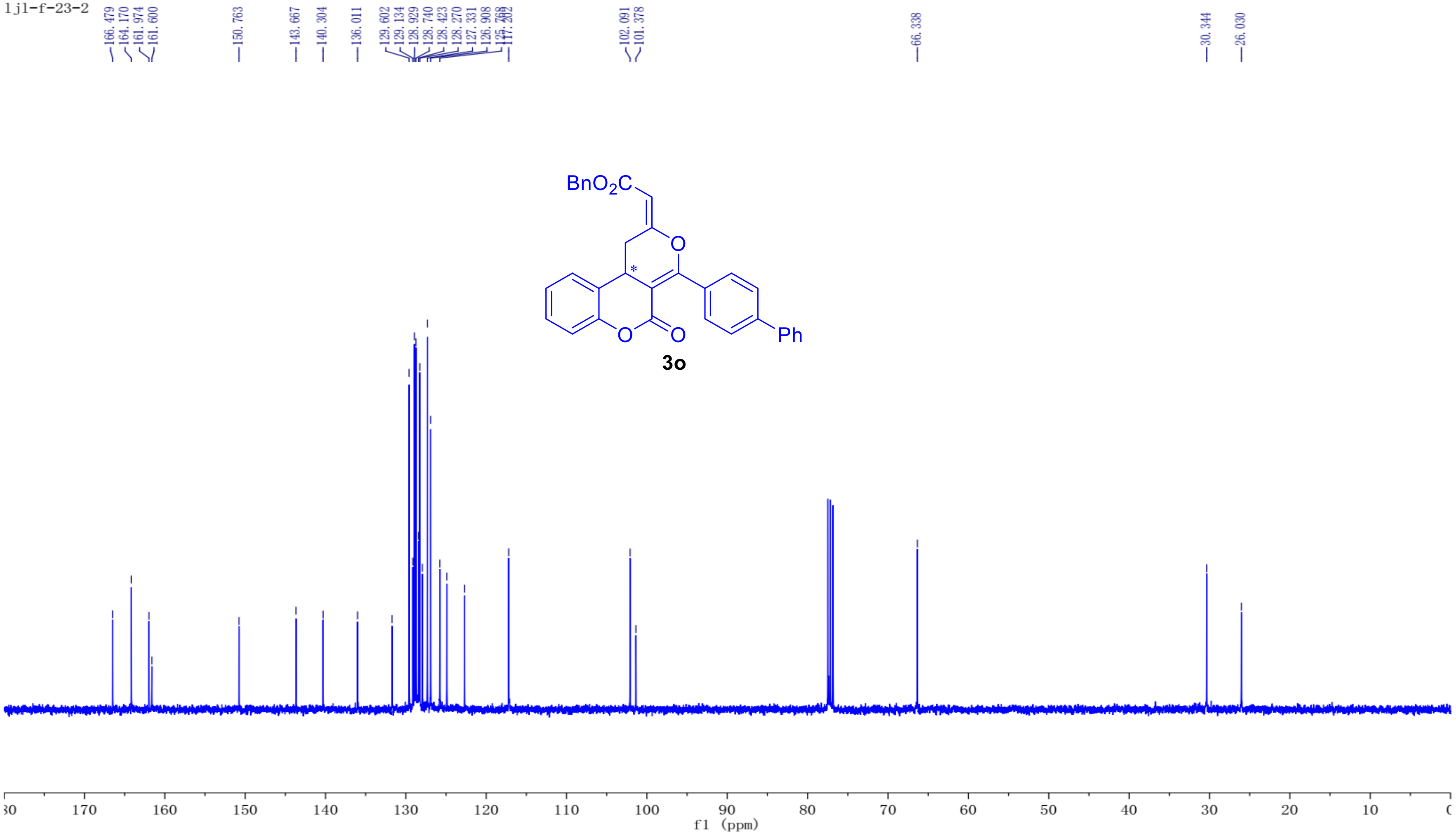

^{1}H NMR Spectrum (400 MHz, Chloroform-*d*) of **3p**

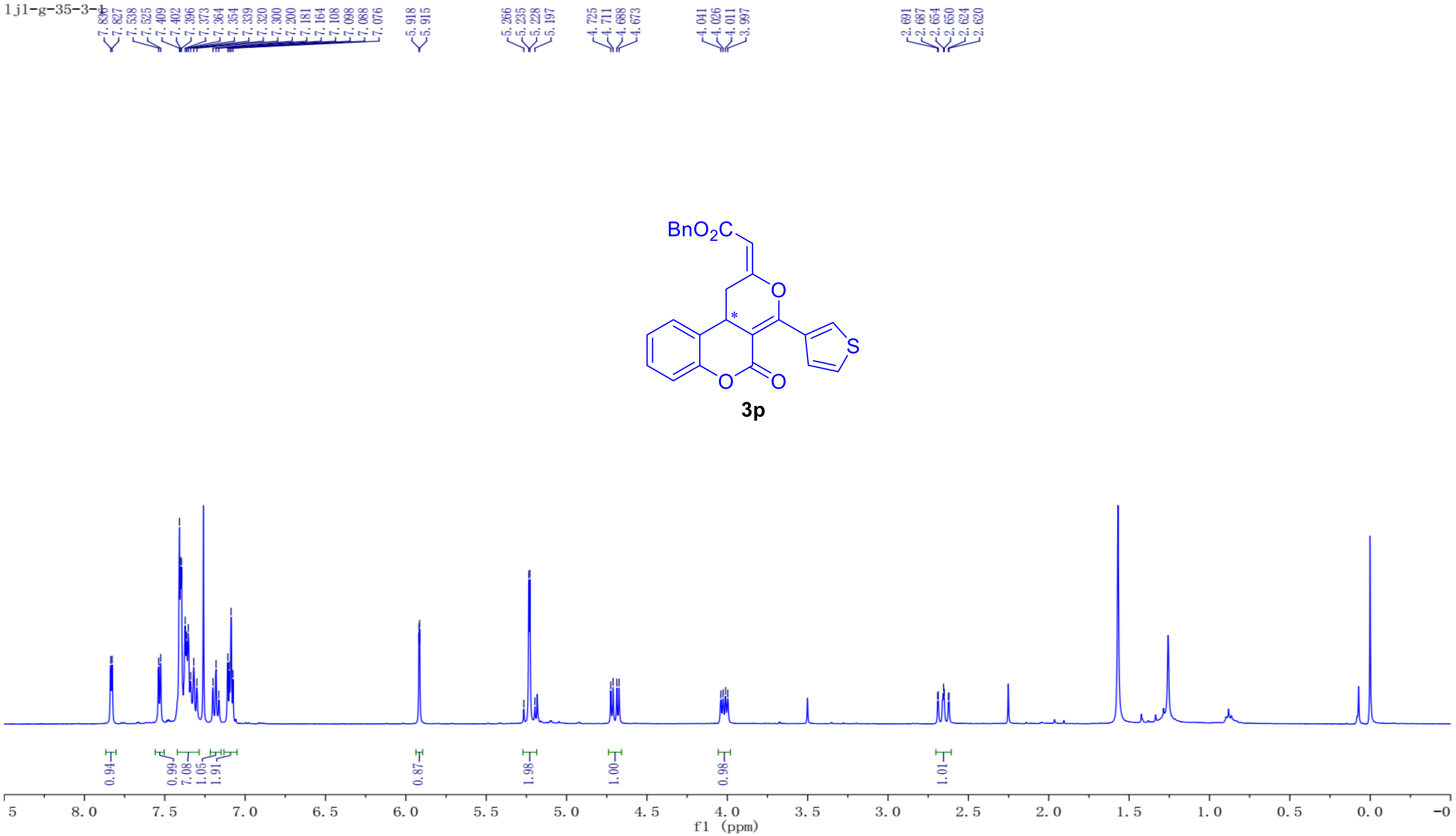

$^{13}C\{^{1}H\}$ NMR Spectrum (101 MHz, Chloroform-*d*) of **3p**

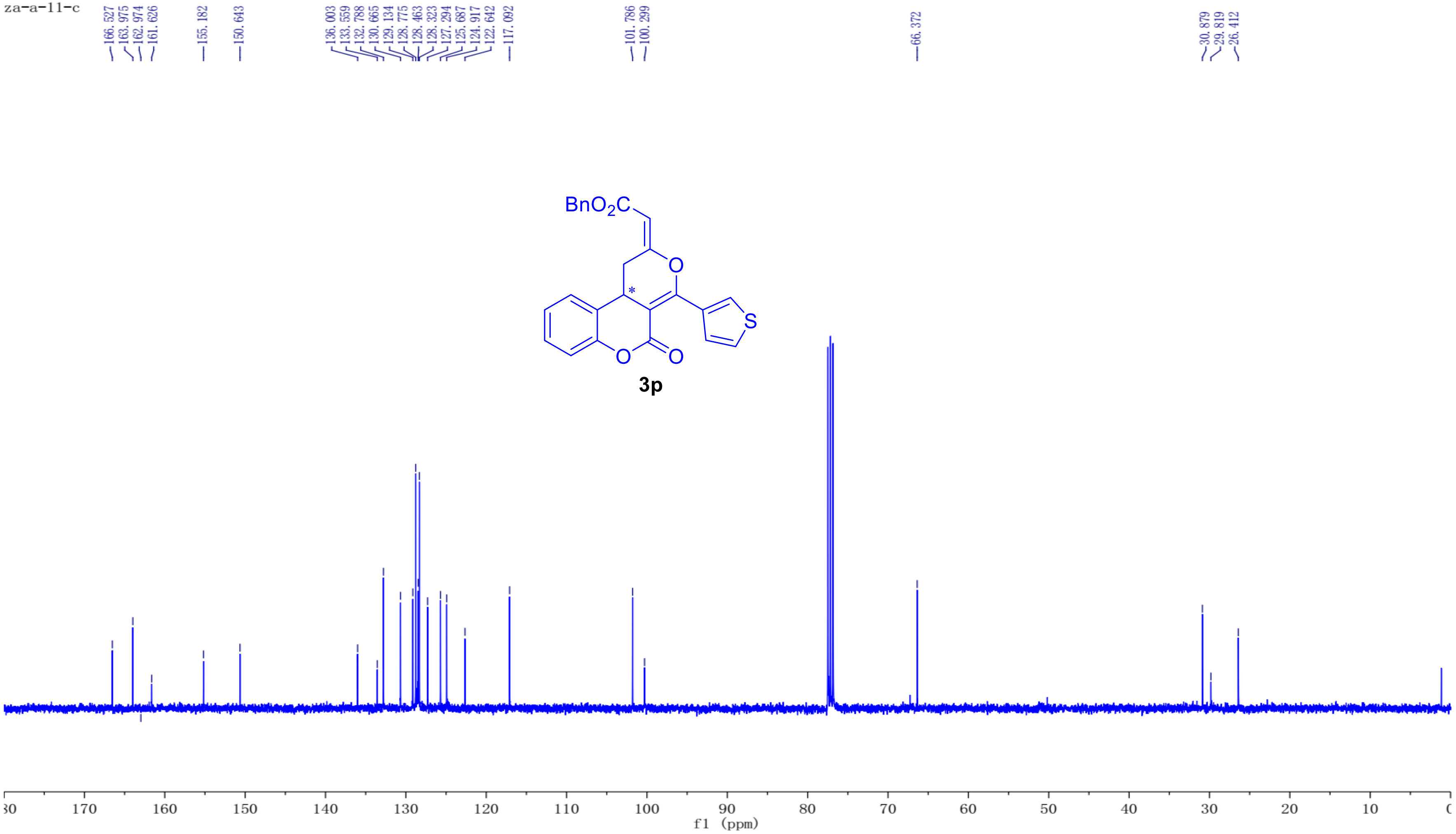

^{1}H NMR Spectrum (400 MHz, Chloroform-*d*) of **5**

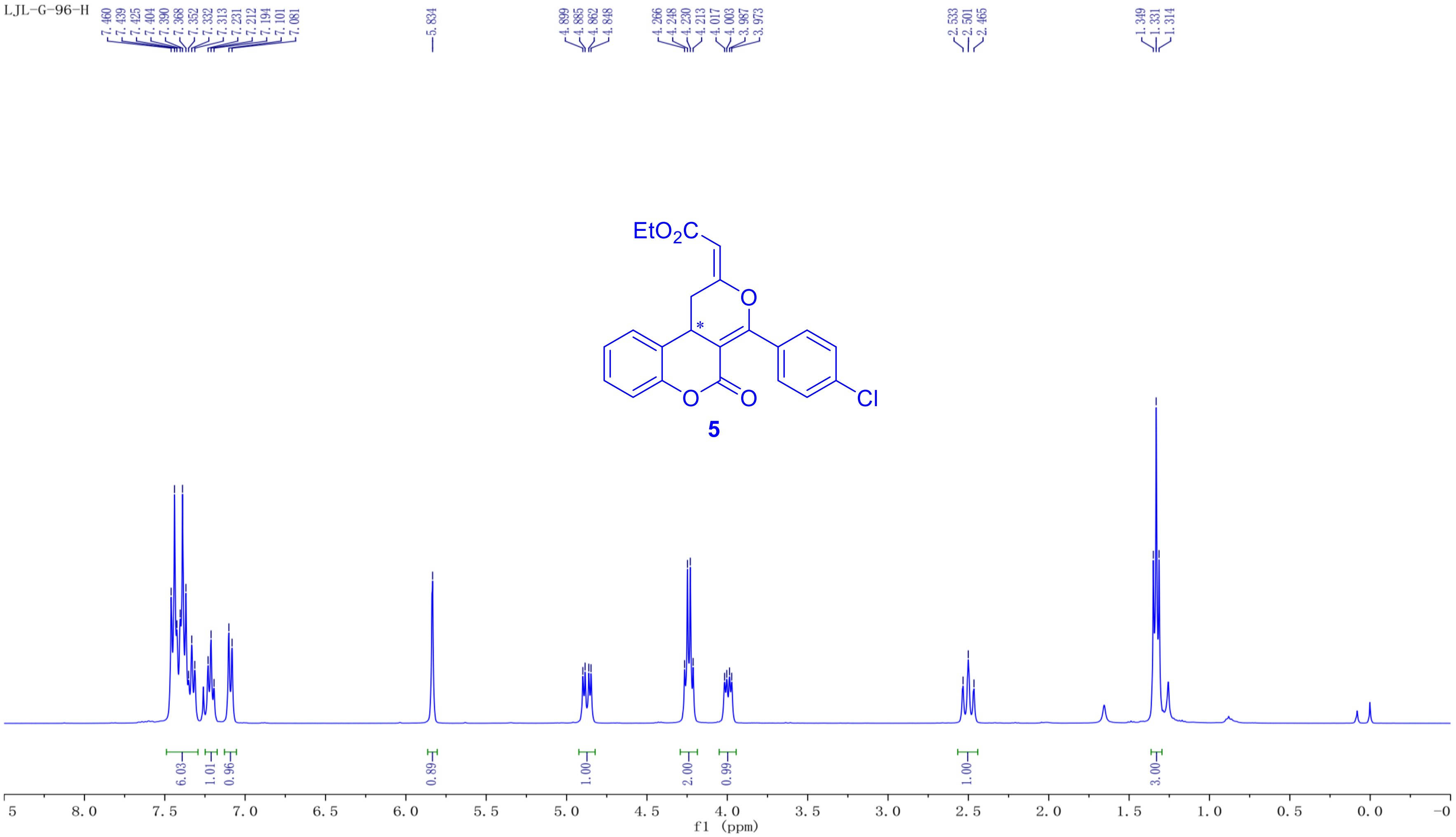

^{13}C {^{1}H} NMR Spectrum (101 MHz, Chloroform-*d*) of **5**

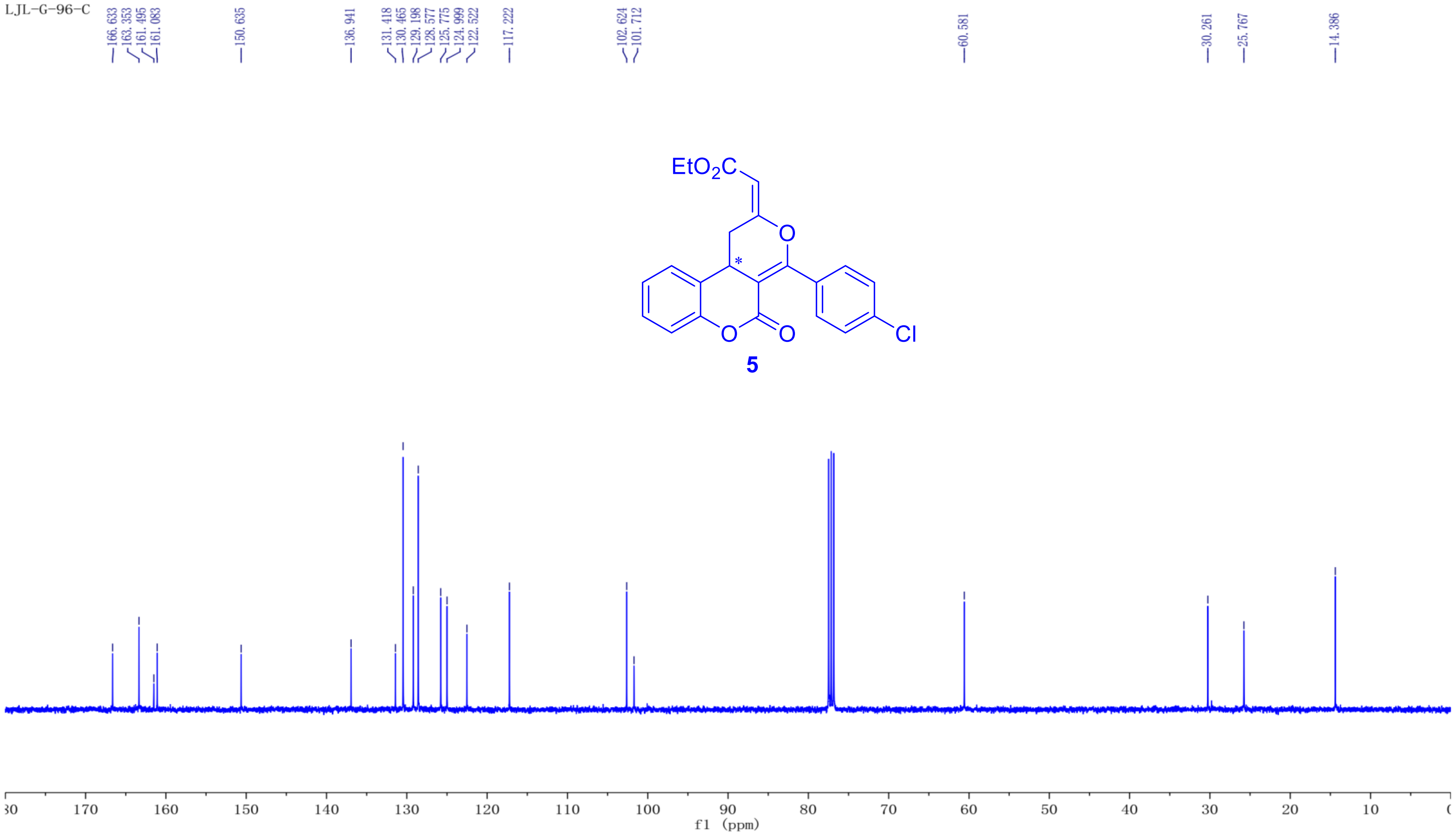

数据文件: C:\CHEM32\1\DATA\LJL\LJL-G-30-2-RAC-AD-H-40_60-0_75-250 203 2020-10-08.D
样品名称: ljl-g-30-2-rac-ad-h-40/60-0.75-250

操作者 : 系统
仪器 : 1260 位置 : 样品瓶 1
进样日期 : 2020-10-8 15:51:34
进样量 : 没有进样
采集方法 : C:\CHEM32\1\METHODS\DEF_LC.M
最后修改 : 2020-10-8 15:04:41 : 系统
(调用后修改)
分析方法 : C:\CHEM32\1\METHODS\DEF_LC.M
附加信息: 峰被手动积分

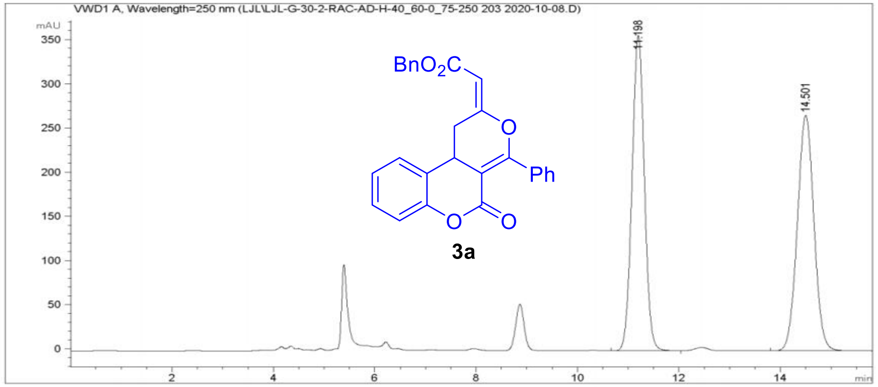

面积百分比报告

排序 : 信号
乘积因子 : 1.0000
稀释因子 : 1.0000
内标使用乘积因子和稀释因子

信号 1: VWD1 A, Wavelength=250 nm

峰 #	保留时间 [min]	类型	峰宽 [min]	峰面积 [mAU*s]	峰高 [mAU]	峰面积 %
1	11.198	BB	0.2627	6044.30029	356.64221	49.9051
2	14.501	BBA	0.3536	6067.28369	266.55814	50.0949

1260 2020-10-8 16:10:06 系统 页 1/2

数据文件: C:\CHEM32\1\DATA\LJL\LJL-D-86-6-40_60-0_75-AD-H-205-ASY 273 2019-10-23.D
样品名称: LJL-D-86-6-40/60-0.75-AD-H-205-ASY

操作者 : 系统
仪器 : 1260 位置 : 样品瓶 1
进样日期 : 2019-10-23 19:33:15
进样量 : 没有进样
采集方法 : C:\CHEM32\1\METHODS\DEF_LC.M
最后修改 : 2019-10-23 19:03:15 : 系统
(调用后修改)
分析方法 : C:\CHEM32\1\METHODS\DEF_LC.M
附加信息: 峰被手动积分

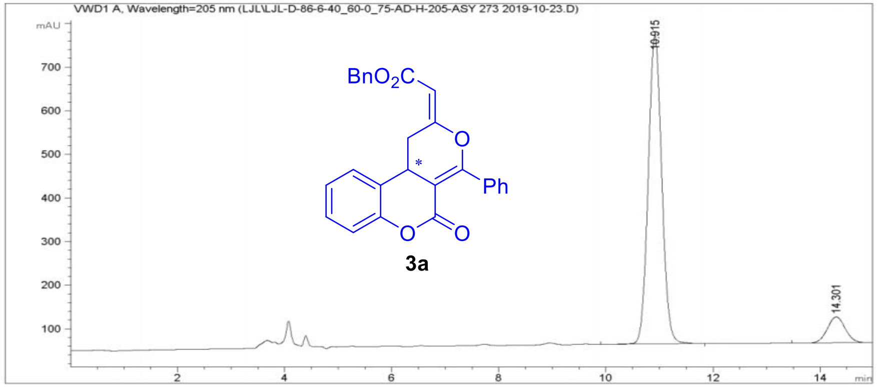

面积百分比报告

排序 : 信号
乘积因子 : 1.0000
稀释因子 : 1.0000
内标使用乘积因子和稀释因子

信号 1: VWD1 A, Wavelength=205 nm

峰 #	保留时间 [min]	类型	峰宽 [min]	峰面积 [mAU*s]	峰高 [mAU]	峰面积 %
1	10.915	BB	0.2735	1.24523e4	706.98340	90.0201
2	14.301	BBA	0.3602	1380.50476	59.60043	9.9799

1260 2020-8-17 10:03:10 系统 页 1/2

数据文件: C:\CHEM32\1\DATA\LJL\LJL-E-77-4-RAC-AD-H-40_60-0_75-250 649 2019-12-30.D
样品名称: ljl-e-77-4-RAC-AD-H-40/60-0.75-250

操作者 : 系统
仪器 : 1260 位置 : 样品瓶 1
进样日期 : 2019-12-30 10:21:57
进样量 : 没有进样
采集方法 : C:\CHEM32\1\METHODS\TEST.M
最后修改 : 2019-12-30 9:27:51 : 系统
(调用后修改)
分析方法 : C:\CHEM32\1\METHODS\TEST.M
最后修改 : 2019-12-27 10:44:45 : 系统
附加信息: 峰被手动积分

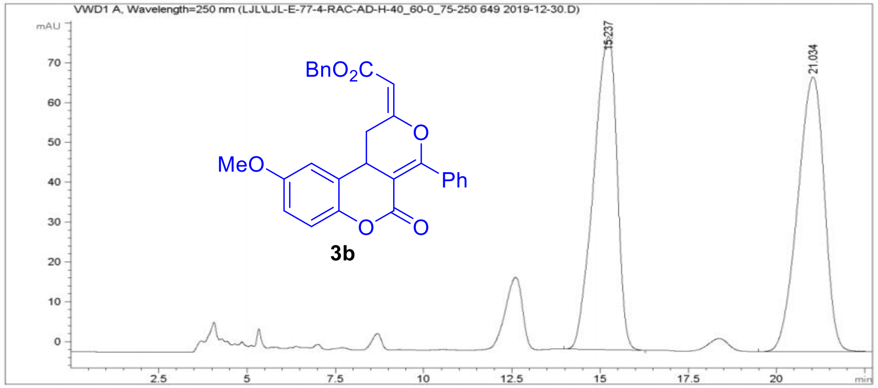

面积百分比报告

排序 : 信号
乘积因子 : 1.0000
稀释因子 : 1.0000
内标使用乘积因子和稀释因子

信号 1: VWD1 A, Wavelength=250 nm

峰 #	保留时间 [min]	类型	峰宽 [min]	峰面积 [mAU*s]	峰高 [mAU]	峰面积 %
1	15.237	BB	0.7171	3601.18848	78.66349	49.8515
2	21.034	BBA	0.8191	3622.63770	68.93123	50.1485

数据文件: C:\CHEM32\1\DATA\LJL\LJL-E-77-4-ASY-AD-H-40_60-0_75-250 650 2019-12-30.D
样品名称: ljl-e-77-4-ASY-AD-H-40/60-0.75-250

操作者 : 系统
仪器 : 1260 位置 : 样品瓶 1
进样日期 : 2019-12-30 10:45:22
进样量 : 没有进样
采集方法 : C:\CHEM32\1\METHODS\TEST.M
最后修改 : 2019-12-30 9:27:51 : 系统
(调用后修改)
分析方法 : C:\CHEM32\1\METHODS\TEST.M
最后修改 : 2019-12-27 10:44:45 : 系统
附加信息: 峰被手动积分

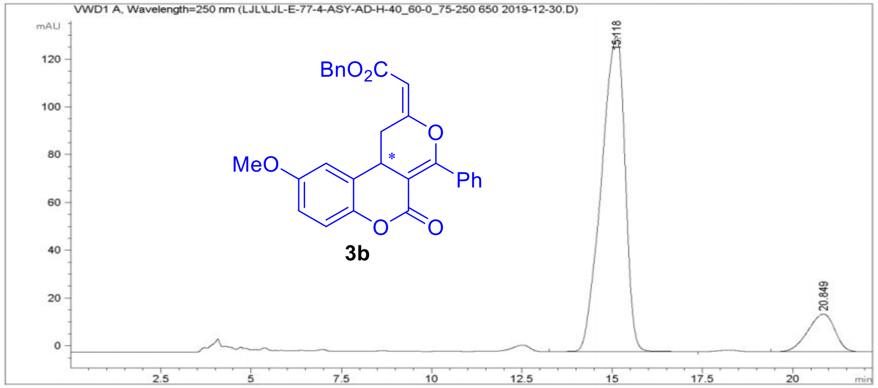

面积百分比报告

排序 : 信号
乘积因子 : 1.0000
稀释因子 : 1.0000
内标使用乘积因子和稀释因子

信号 1: VWD1 A, Wavelength=250 nm

峰 #	保留时间 [min]	类型	峰宽 [min]	峰面积 [mAU*s]	峰高 [mAU]	峰面积 %
1	15.118	BB	0.7164	6022.98975	131.98674	88.0203
2	20.849	BBA	0.8122	819.73871	15.74980	11.9797

数据文件: C:\CHEM32\1\DATA\LJL\LJL-E-79-1-RAC-0_75-40_60-250-AD-H 685 2020-01-03.D
样品名称: ljl-e-79-1-rac-0.75-40/60-250-ad-h

==

操作者 : 系统
仪器 : 1260 位置 : 样品瓶 1
进样日期 : 2020-1-3 16:17:17
进样量 : 没有进样
采集方法 : C:\CHEM32\1\METHODS\DEF_LC.M
最后修改 : 2020-1-3 15:58:14 : 系统
(调用后修改)
分析方法 : C:\CHEM32\1\METHODS\DEF_LC.M
附加信息: 峰被手动积分

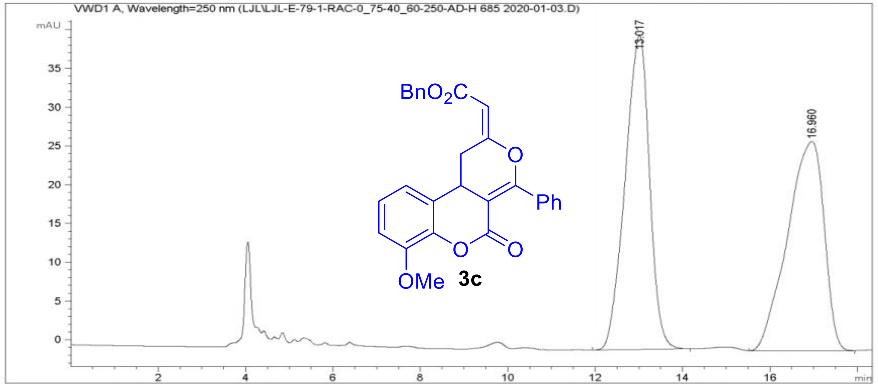

==

面积百分比报告

==

排序 : 信号
乘积因子 : 1.0000
稀释因子 : 1.0000
内标使用乘积因子和稀释因子

信号 1: VWD1 A, Wavelength=250 nm

峰 #	保留时间 [min]	类型	峰宽 [min]	峰面积 [mAU*s]	峰高 [mAU]	峰面积 %
1	13.017	BB	0.6029	1571.54138	40.48283	50.0278
2	16.960	BB	0.9128	1569.79358	26.98207	49.9722

数据文件: C:\CHEM32\1\DATA\LJL\LJL-E-79-1-ASY-0_75-40_60-250-AD-H 686 2020-01-03.D
样品名称: ljl-e-79-1-asy-0.75-40/60-250-ad-h

==

操作者 : 系统
仪器 : 1260 位置 : 样品瓶 1
进样日期 : 2020-1-3 16:37:33
进样量 : 没有进样
采集方法 : C:\CHEM32\1\METHODS\DEF_LC.M
最后修改 : 2020-1-3 15:58:14 : 系统
(调用后修改)
分析方法 : C:\CHEM32\1\METHODS\DEF_LC.M
附加信息: 峰被手动积分

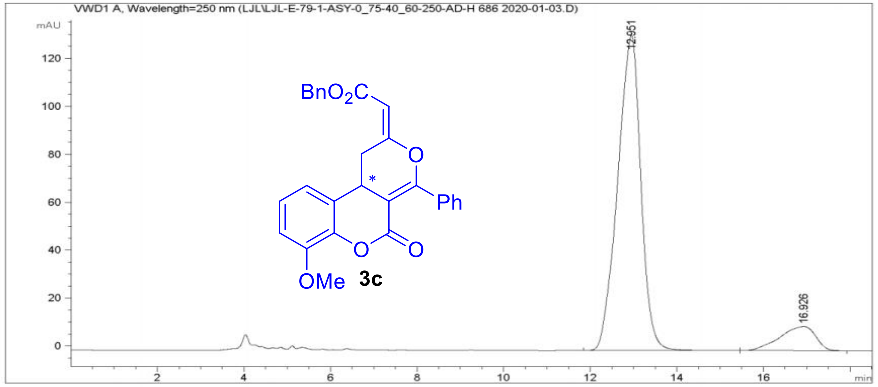

==

面积百分比报告

==

排序 : 信号
乘积因子 : 1.0000
稀释因子 : 1.0000
内标使用乘积因子和稀释因子

信号 1: VWD1 A, Wavelength=250 nm

峰 #	保留时间 [min]	类型	峰宽 [min]	峰面积 [mAU*s]	峰高 [mAU]	峰面积 %
1	12.951	BB	0.5774	4887.79150	131.28285	89.4149
2	16.926	BB	0.8951	578.62482	10.05914	10.5851

数据文件: C:\CHEM32\1\DATA\LJL\LJL-E-79-4-RAC-0_75-40_60-250-AD-H 691 2020-01-03.D
样品名称: ljl-e-79-4-rac-0.75-40/60-250-ad-h

===

操作者 : 系统
仪器 : 1260 位置 : 样品瓶 1
进样日期 : 2020-1-3 19:55:39
进样量 : 没有进样
采集方法 : C:\CHEM32\1\METHODS\DEF_LC.M
最后修改 : 2020-1-3 19:14:27 : 系统
(调用后修改)
分析方法 : C:\CHEM32\1\METHODS\DEF_LC.M
附加信息: 峰被手动积分

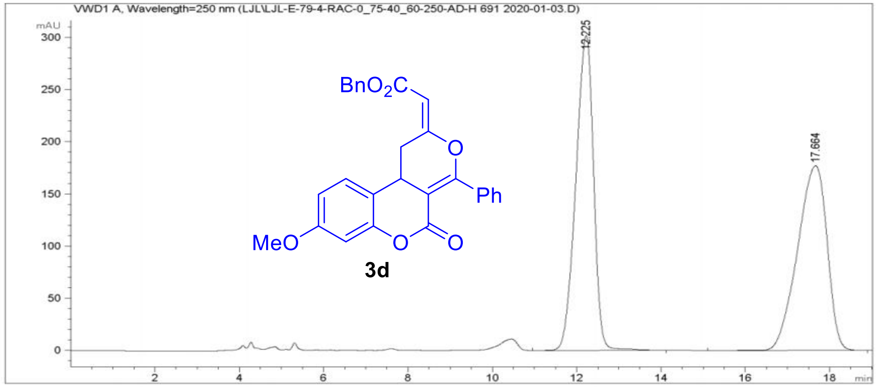

===
面积百分比报告
===

排序 : 信号
乘积因子 : 1.0000
稀释因子 : 1.0000
内标使用乘积因子和稀释因子

信号 1: VWD1 A, Wavelength=250 nm

峰 #	保留时间 [min]	类型	峰宽 [min]	峰面积 [mAU*s]	峰高 [mAU]	峰面积 %
1	12.225	BB	0.4573	8854.05859	301.75830	50.6439
2	17.664	BB	0.7612	8628.91406	177.17479	49.3561

1260 2020-1-3 20:42:45 系统 页 1/2

数据文件: C:\CHEM32\1\DATA\LJL\LJL-E-79-4-ASY-0_75-40_60-250-AD-H 692 2020-01-03.D
样品名称: ljl-e-79-4-asy-0.75-40/60-250-ad-h

===

操作者 : 系统
仪器 : 1260 位置 : 样品瓶 1
进样日期 : 2020-1-3 20:16:02
进样量 : 没有进样
采集方法 : C:\CHEM32\1\METHODS\DEF_LC.M
最后修改 : 2020-1-3 19:14:27 : 系统
(调用后修改)
分析方法 : C:\CHEM32\1\METHODS\DEF_LC.M
附加信息: 峰被手动积分

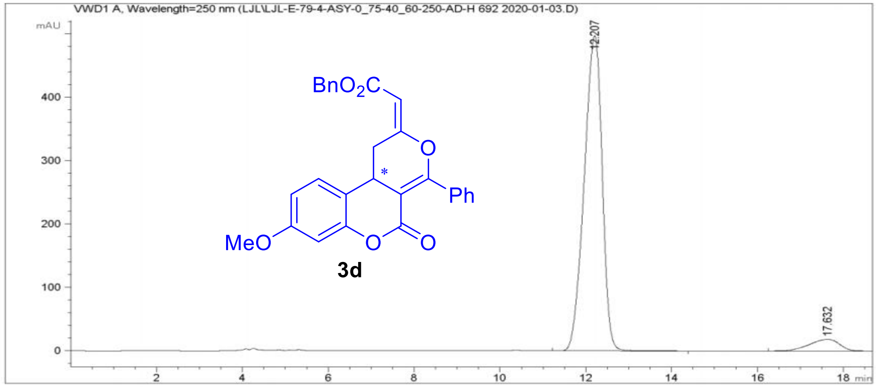

===
面积百分比报告
===

排序 : 信号
乘积因子 : 1.0000
稀释因子 : 1.0000
内标使用乘积因子和稀释因子

信号 1: VWD1 A, Wavelength=250 nm

峰 #	保留时间 [min]	类型	峰宽 [min]	峰面积 [mAU*s]	峰高 [mAU]	峰面积 %
1	12.207	BB	0.4539	1.44656e4	497.91467	94.1834
2	17.632	BBA	0.7600	893.36340	18.34962	5.8166

1260 2020-1-3 20:43:00 系统 页 1/2

数据文件：C:\CHEM32\1\DATA\LJL\LJL-E-77-3-RAC-AD-H-40_60-0_75-250 647 2019-12-30.D
样品名称：ljl-e-77-3-RAC-AD-H-40/60-0.75-250

==

操作者	：系统		
仪器	：1260	位置	：样品瓶 1
进样日期	：2019-12-30 9:39:08		
		进样量	：没有进样
采集方法	：C:\CHEM32\1\METHODS\TEST.M		
最后修改	：2019-12-30 9:27:51 ：系统 (调用后修改)		
分析方法	：C:\CHEM32\1\METHODS\TEST.M		
最后修改	：2019-12-27 10:44:45 ：系统		

附加信息：峰被手动积分

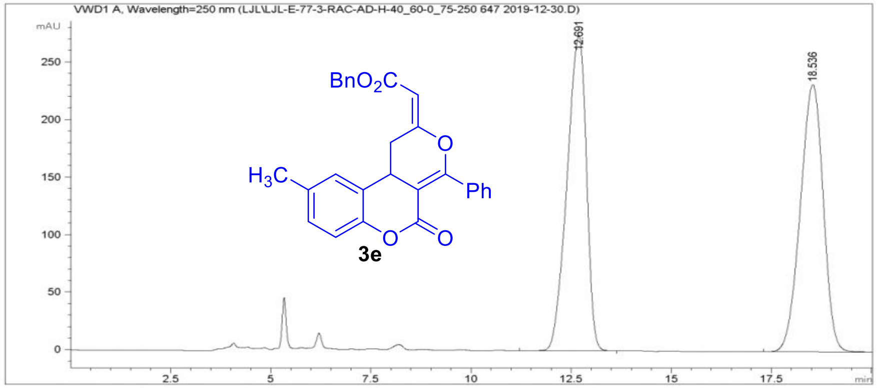

==

面积百分比报告

==

排序 ： 信号
乘积因子 ： 1.0000
稀释因子 ： 1.0000
内标使用乘积因子和稀释因子

信号 1：VWD1 A, Wavelength=250 nm

峰 #	保留时间 [min]	类型	峰宽 [min]	峰面积 [mAU*s]	峰高 [mAU]	峰面积 %
1	12.691	BB	0.5288	9254.33105	273.44775	50.0557
2	18.536	BBA	0.6248	9233.73730	232.26074	49.9443

数据文件：C:\CHEM32\1\DATA\LJL\LJL-E-77-3-ASY-AD-H-40_60-0_75-250 648 2019-12-30.D
样品名称：ljl-e-77-3-ASY-AD-H-40/60-0.75-250

==

操作者	：系统		
仪器	：1260	位置	：样品瓶 1
进样日期	：2019-12-30 10:00:43		
		进样量	：没有进样
采集方法	：C:\CHEM32\1\METHODS\TEST.M		
最后修改	：2019-12-30 9:27:51 ：系统 (调用后修改)		
分析方法	：C:\CHEM32\1\METHODS\TEST.M		
最后修改	：2019-12-27 10:44:45 ：系统		

附加信息：峰被手动积分

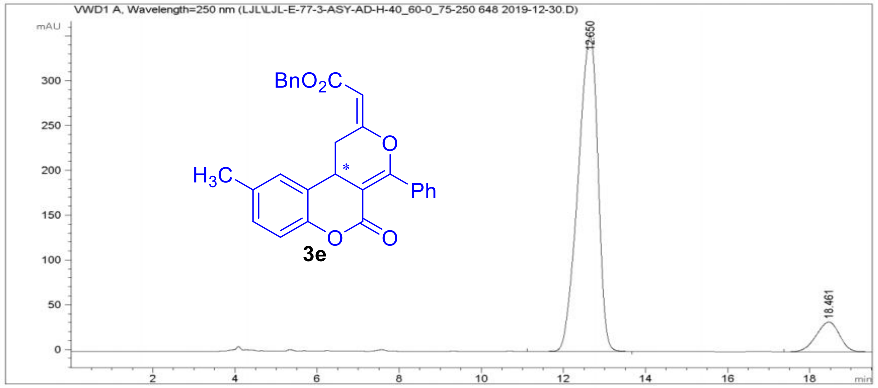

==

面积百分比报告

==

排序 ： 信号
乘积因子 ： 1.0000
稀释因子 ： 1.0000
内标使用乘积因子和稀释因子

信号 1：VWD1 A, Wavelength=250 nm

峰 #	保留时间 [min]	类型	峰宽 [min]	峰面积 [mAU*s]	峰高 [mAU]	峰面积 %
1	12.650	BB	0.5276	1.18320e4	351.61151	90.0583
2	18.461	BBA	0.6150	1306.15039	33.12396	9.9417

数据文件：C:\CHEM32\1\DATA\LJL\LJL-E-84-2-AD-H-40_60-RAC-0_75-250 813 2020-05-30.D
样品名称：LJL-E-84-2-AD-H-40/60-rac-0.75-250

===

操作者 : 系统
仪器 : 1260　　　位置 : 样品瓶 1
进样日期 : 2020-5-30 15:55:38
　　　进样量 : 没有进样
采集方法 : C:\CHEM32\1\METHODS\DEF_LC.M
最后修改 : 2020-5-30 15:05:28 : 系统
(调用后修改)
分析方法 : C:\CHEM32\1\METHODS\DEF_LC.M
附加信息：峰被手动积分

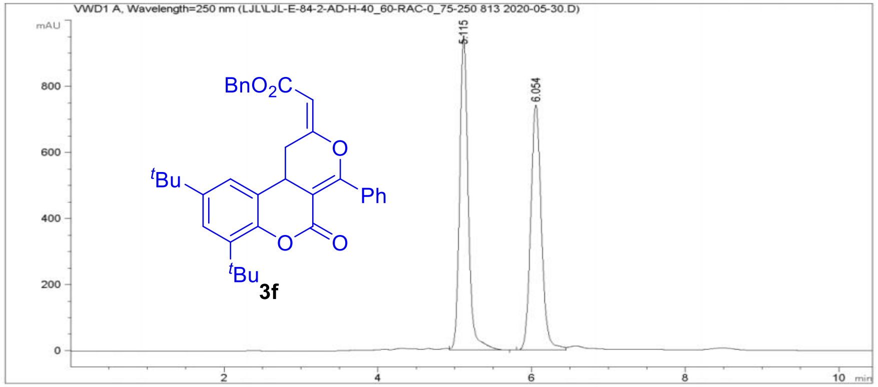

===
面积百分比报告
===

排序 : 信号
乘积因子 : 1.0000
稀释因子 : 1.0000
内标使用乘积因子和稀释因子

信号 1: VWD1 A, Wavelength=250 nm

峰 #	保留时间 [min]	类型	峰宽 [min]	峰面积 [mAU*s]	峰高 [mAU]	峰面积 %
1	5.115	VB	0.1182	7350.38867	950.79443	50.8212
2	6.054	BV	0.1479	7112.83740	741.40869	49.1788

数据文件：C:\CHEM32\1\DATA\LJL\LJL-E-84-2-AD-H-40_60-ASY-0_75-250 814 2020-05-30.D
样品名称：LJL-E-84-2-AD-H-40/60-asy-0.75-250

===

操作者 : 系统
仪器 : 1260　　　位置 : 样品瓶 1
进样日期 : 2020-5-30 16:06:43
　　　进样量 : 没有进样
采集方法 : C:\CHEM32\1\METHODS\DEF_LC.M
最后修改 : 2020-5-30 15:05:28 : 系统
(调用后修改)
分析方法 : C:\CHEM32\1\METHODS\DEF_LC.M
附加信息：峰被手动积分

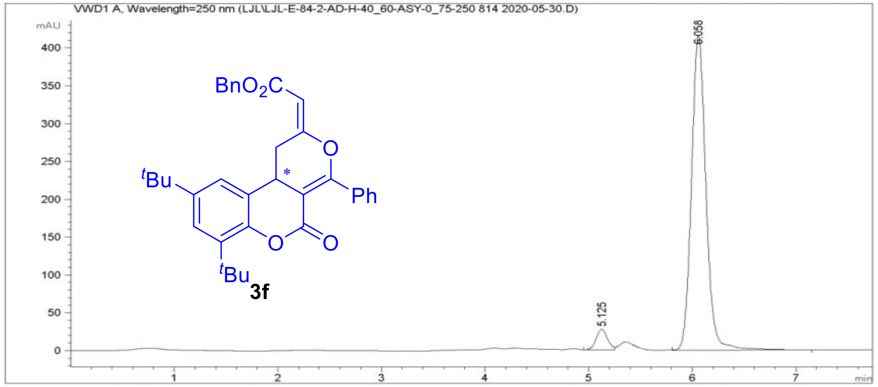

===
面积百分比报告
===

排序 : 信号
乘积因子 : 1.0000
稀释因子 : 1.0000
内标使用乘积因子和稀释因子

信号 1: VWD1 A, Wavelength=250 nm

峰 #	保留时间 [min]	类型	峰宽 [min]	峰面积 [mAU*s]	峰高 [mAU]	峰面积 %
1	5.125	BV	0.1168	204.84067	27.21048	4.8293
2	6.058	VB	0.1493	4036.81982	415.63434	95.1707

数据文件：C:\CHEM32\1\DATA\LJL\LJL-E-77-1-RAC-AD-H-40_60--0_75-250 659 2019-12-30.D
样品名称：LJL-E-77-1-RAC-AD-H-40/60--0.75-250

===

操作者 ： 系统
仪器 ： 1260 位置 ： 样品瓶 1
进样日期 ： 2019-12-30 16:01:38
进样量 ： 没有进样
采集方法 ： C:\CHEM32\1\METHODS\TEST.M
最后修改 ： 2019-12-30 15:49:06 ： 系统
（调用后修改）
分析方法 ： C:\CHEM32\1\METHODS\TEST.M
最后修改 ： 2019-12-27 10:44:45 ： 系统
附加信息：峰被手动积分

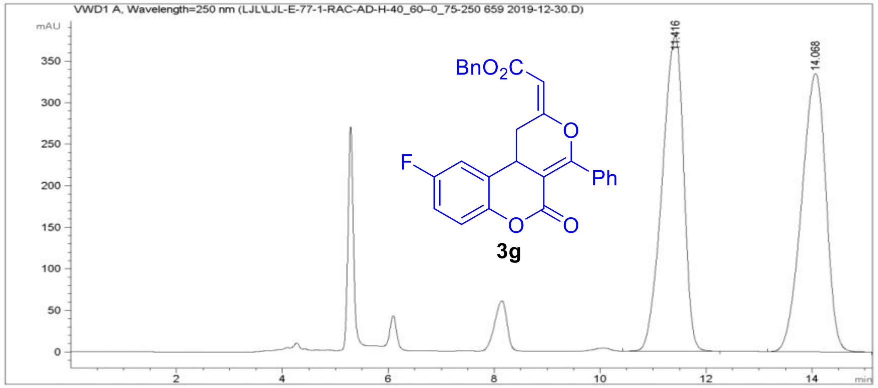

===
面积百分比报告
===

排序 ： 信号
乘积因子 ： 1.0000
稀释因子 ： 1.0000
内标使用乘积因子和稀释因子

信号 1：VWD1 A, Wavelength=250 nm

峰 #	保留时间 [min]	类型	峰宽 [min]	峰面积 [mAU*s]	峰高 [mAU]	峰面积 %
1	11.416	BB	0.4429	1.06745e4	378.70020	50.0769
2	14.068	BBA	0.5011	1.06417e4	334.55179	49.9231

1260 2019-12-30 17:08:48 系统 页 1/2

数据文件：C:\CHEM32\1\DATA\LJL\LJL-E-77-1-ASY-AD-H-40_60--0_75-250 661 2019-12-30.D
样品名称：LJL-E-77-1-ASY-AD-H-40/60--0.75-250

===

操作者 ： 系统
仪器 ： 1260 位置 ： 样品瓶 1
进样日期 ： 2019-12-30 16:38:03
进样量 ： 没有进样
采集方法 ： C:\CHEM32\1\METHODS\TEST.M
最后修改 ： 2019-12-30 15:49:06 ： 系统
（调用后修改）
分析方法 ： C:\CHEM32\1\METHODS\TEST.M
最后修改 ： 2019-12-27 10:44:45 ： 系统
附加信息：峰被手动积分

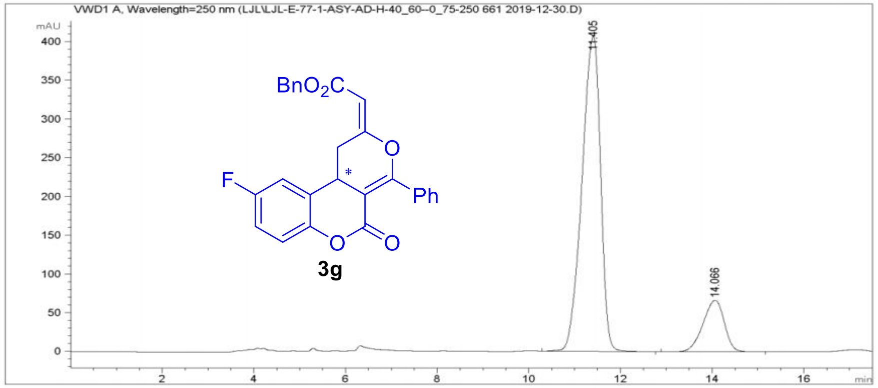

===
面积百分比报告
===

排序 ： 信号
乘积因子 ： 1.0000
稀释因子 ： 1.0000
内标使用乘积因子和稀释因子

信号 1：VWD1 A, Wavelength=250 nm

峰 #	保留时间 [min]	类型	峰宽 [min]	峰面积 [mAU*s]	峰高 [mAU]	峰面积 %
1	11.405	BB	0.4427	1.14646e4	406.88049	84.3072
2	14.066	BB	0.5057	2134.00732	66.27591	15.6928

1260 2019-12-30 17:09:18 系统 页 1/2

数据文件：C:\CHEM32\1\DATA\LJL\LJL-E-77-5-RAC-AD-H-40_60-0_75-250 651 2019-12-30.D
样品名称：ljl-e-77-5-RAC-AD-H-40/60-0.75-250

操作者 ： 系统
仪器 ： 1260 位置 ： 样品瓶 1
进样日期 ： 2019-12-30 11:08:52
进样量 ： 没有进样
采集方法 ： C:\CHEM32\1\METHODS\TEST.M
最后修改 ： 2019-12-30 9:27:51 ： 系统
（调用后修改）
分析方法 ： C:\CHEM32\1\METHODS\TEST.M
最后修改 ： 2019-12-27 10:44:45 ： 系统
附加信息：峰被手动积分

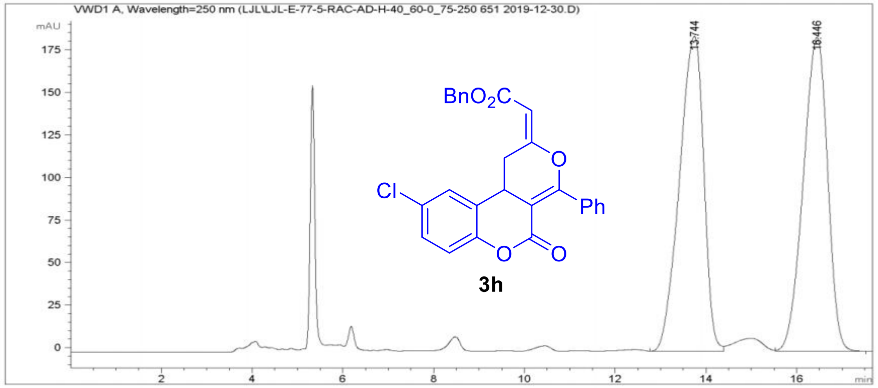

面积百分比报告

排序 ： 信号
乘积因子 ： 1.0000
稀释因子 ： 1.0000
内标使用乘积因子和稀释因子

信号 1：VWD1 A, Wavelength=250 nm

峰 #	保留时间 [min]	类型	峰宽 [min]	峰面积 [mAU*s]	峰高 [mAU]	峰面积 %
1	13.744	BV	0.5856	6888.02686	184.91417	50.0832
2	16.446	VB	0.5856	6865.13135	184.27557	49.9168

数据文件：C:\CHEM32\1\DATA\LJL\LJL-E-77-5-ASY-AD-H-40_60-0_75-250 652 2019-12-30.D
样品名称：ljl-e-77-5-ASY-AD-H-40/60-0.75-250

操作者 ： 系统
仪器 ： 1260 位置 ： 样品瓶 1
进样日期 ： 2019-12-30 11:28:12
进样量 ： 没有进样
采集方法 ： C:\CHEM32\1\METHODS\TEST.M
最后修改 ： 2019-12-30 9:27:51 ： 系统
（调用后修改）
分析方法 ： C:\CHEM32\1\METHODS\TEST.M
最后修改 ： 2019-12-27 10:44:45 ： 系统
附加信息：峰被手动积分

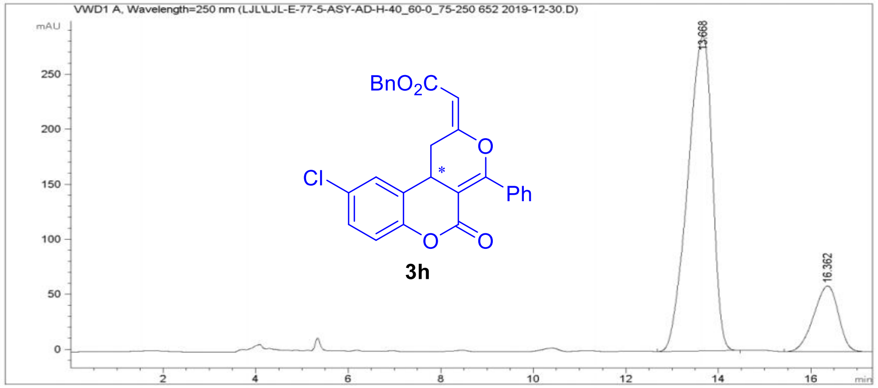

面积百分比报告

排序 ： 信号
乘积因子 ： 1.0000
稀释因子 ： 1.0000
内标使用乘积因子和稀释因子

信号 1：VWD1 A, Wavelength=250 nm

峰 #	保留时间 [min]	类型	峰宽 [min]	峰面积 [mAU*s]	峰高 [mAU]	峰面积 %
1	13.668	BB	0.5767	1.05095e4	285.98181	82.6201
2	16.362	BBA	0.5810	2210.77344	59.70735	17.3799

数据文件: C:\CHEM32\1\DATA\LJL\LJL-E-86-2-RAC-AD-H-40_60-0_75-250 760 2020-01-15.D
样品名称: ljl-e-86-2-rac-ad-h-40/60-0.75-250

===

操作者 : 系统
仪器 : 1260 位置 : 样品瓶 1
进样日期 : 2020-1-15 16:50:12
进样量 : 没有进样
采集方法 : C:\CHEM32\1\METHODS\DEF_LC.M
最后修改 : 2020-1-15 16:22:53 : 系统
(调用后修改)
分析方法 : C:\CHEM32\1\METHODS\DEF_LC.M
附加信息: 峰被手动积分

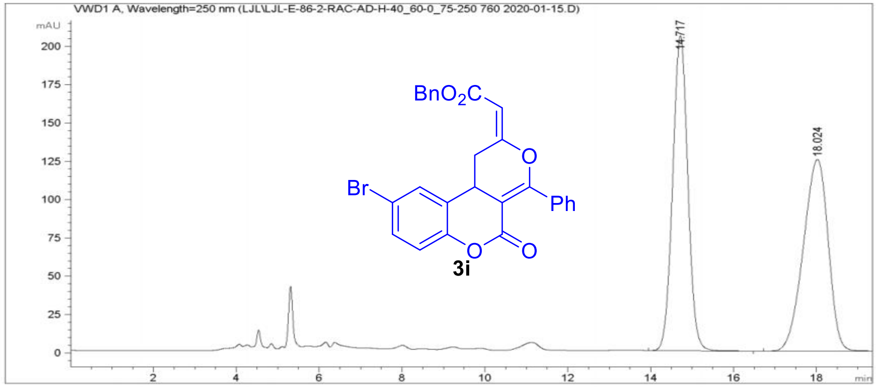

===

面积百分比报告

===

排序 : 信号
乘积因子 : 1.0000
稀释因子 : 1.0000
内标使用乘积因子和稀释因子

信号 1: VWD1 A, Wavelength=250 nm

峰 #	保留时间 [min]	类型	峰宽 [min]	峰面积 [mAU*s]	峰高 [mAU]	峰面积 %
1	14.717	BB	0.3935	5224.42041	205.56592	50.0855
2	18.024	BBA	0.6541	5206.57617	124.96338	49.9145

数据文件: C:\CHEM32\1\DATA\LJL\LJL-E-77-2-ASY-AD-H-40_60--0_75-250 662 2019-12-30.D
样品名称: LJL-E-77-2-ASY-AD-H-40/60--0.75-250

===

操作者 : 系统
仪器 : 1260 位置 : 样品瓶 1
进样日期 : 2019-12-30 16:58:07
进样量 : 没有进样
采集方法 : C:\CHEM32\1\METHODS\TEST.M
最后修改 : 2019-12-30 15:49:06 : 系统
(调用后修改)
分析方法 : C:\CHEM32\1\METHODS\TEST.M
最后修改 : 2019-12-27 10:44:45 : 系统
附加信息: 峰被手动积分

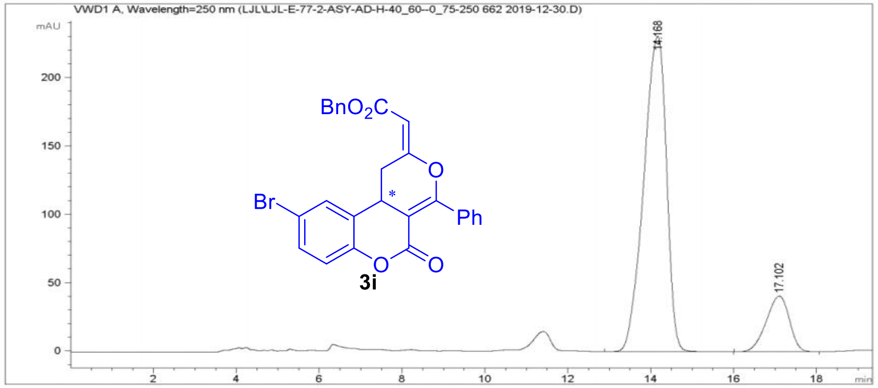

===

面积百分比报告

===

排序 : 信号
乘积因子 : 1.0000
稀释因子 : 1.0000
内标使用乘积因子和稀释因子

信号 1: VWD1 A, Wavelength=250 nm

峰 #	保留时间 [min]	类型	峰宽 [min]	峰面积 [mAU*s]	峰高 [mAU]	峰面积 %
1	14.168	BB	0.6011	8797.71680	230.58594	84.7841
2	17.102	BB	0.6113	1578.89478	40.72634	15.2159

数据文件：C:\CHEM32\1\DATA\LJL\LJL-E-86-2-RAC-AD-H-40_60-0_75-250 760 2020-01-15.D
样品名称：ljl-e-86-2-rac-ad-h-40/60-0.75-250

===

操作者 ： 系统
仪器 ： 1260 位置 ： 样品瓶 1
进样日期 ： 2020-1-15 16:50:12
进样量 ： 没有进样
采集方法 ： C:\CHEM32\1\METHODS\DEF_LC.M
最后修改 ： 2020-1-15 16:22:53 ： 系统
（调用后修改）
分析方法 ： C:\CHEM32\1\METHODS\DEF_LC.M
附加信息：峰被手动积分

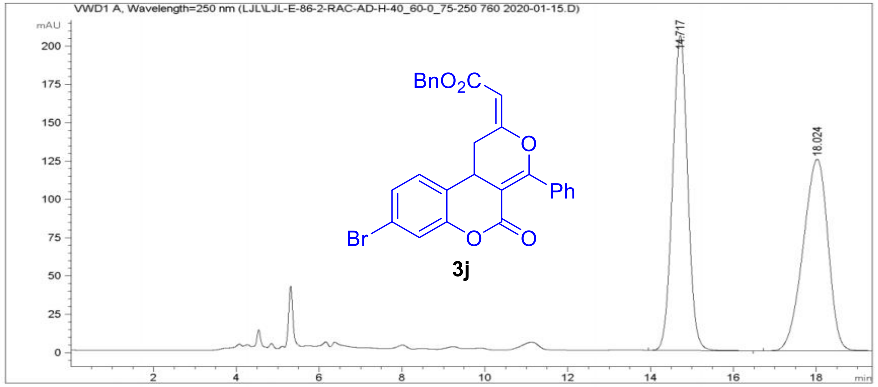

===

面积百分比报告

===

排序 ： 信号
乘积因子 ： 1.0000
稀释因子 ： 1.0000
内标使用乘积因子和稀释因子

信号 1: VWD1 A, Wavelength=250 nm

峰 #	保留时间 [min]	类型	峰宽 [min]	峰面积 [mAU*s]	峰高 [mAU]	峰面积 %
1	14.717	BB	0.3935	5224.42041	205.56592	50.0855
2	18.024	BBA	0.6541	5206.57617	124.96338	49.9145

1260 2020-5-29 21:56:31 系统 页 1/2

数据文件：C:\CHEM32\1\DATA\LJL\LJL-E-86-2-ASY-AD-H-40_60-0_75-250 761 2020-01-15.D
样品名称：ljl-e-86-2-asy-ad-h-40/60-0.75-250

===

操作者 ： 系统
仪器 ： 1260 位置 ： 样品瓶 1
进样日期 ： 2020-1-15 17:10:35
进样量 ： 没有进样
采集方法 ： C:\CHEM32\1\METHODS\DEF_LC.M
最后修改 ： 2020-1-15 16:22:53 ： 系统
（调用后修改）
分析方法 ： C:\CHEM32\1\METHODS\DEF_LC.M
附加信息：峰被手动积分

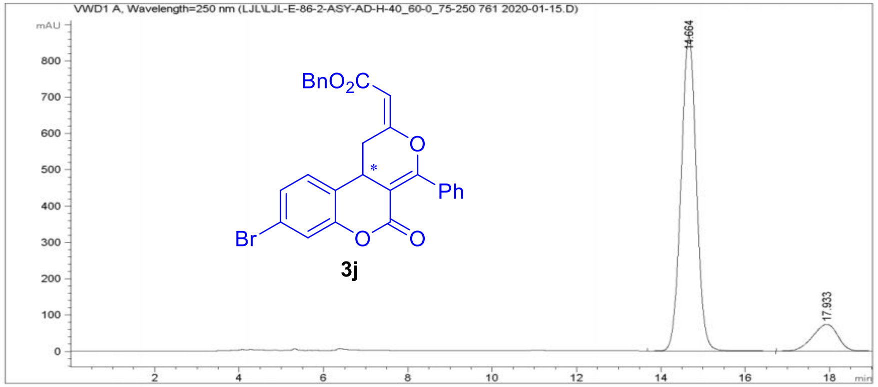

===

面积百分比报告

===

排序 ： 信号
乘积因子 ： 1.0000
稀释因子 ： 1.0000
内标使用乘积因子和稀释因子

信号 1: VWD1 A, Wavelength=250 nm

峰 #	保留时间 [min]	类型	峰宽 [min]	峰面积 [mAU*s]	峰高 [mAU]	峰面积 %
1	14.664	BB	0.3931	2.19542e4	867.99646	87.8843
2	17.933	BBA	0.6500	3026.60913	73.25381	12.1157

1260 2020-1-15 21:56:57 系统 页 1/2

数据文件：C:\CHEM32\1\DATA\LJL\LJL-E-89-1-AD-H-40_60-RAC-0_75-250 827 2020-06-01.D
样品名称：LJL-E-89-1-AD-H-40/60-rac-0.75-250

===

操作者　　　　：系统
位置　：样品瓶 1
进样日期　　　：2020-6-1 9:33:59
方法　　　　　：C:\CHEM32\1\METHODS\DEF_LC.M
附加信息：峰被手动积分

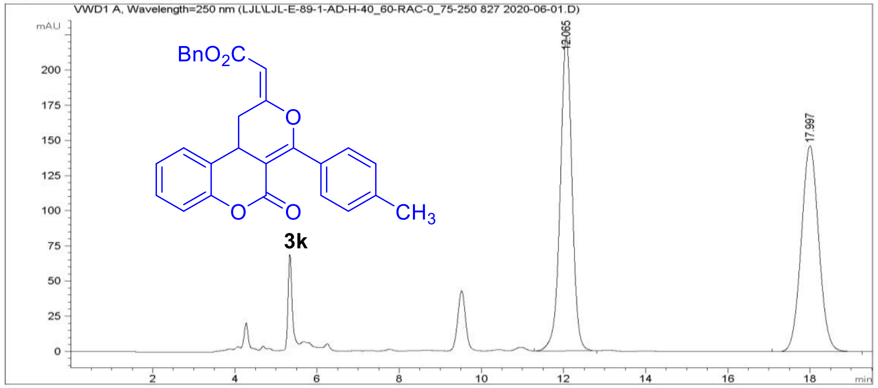

===

面积百分比报告

===

排序　　　：　信号
乘积因子　　：　1.0000
稀释因子　　：　1.0000
内标使用乘积因子和稀释因子

信号 1：VWD1 A, Wavelength=250 nm

峰 #	保留时间 [min]	类型	峰宽 [min]	峰面积 [mAU*s]	峰高 [mAU]	峰面积 %
1	12.065	BB	0.3069	4483.33887	223.66631	50.7598
2	17.997	BB	0.4594	4349.11328	146.47058	49.2402

总量：　8832.45215　370.13689

===

数据文件：C:\CHEM32\1\DATA\LJL\LJL-E-89-1-AD-H-40_60-ASY-0_75-250 828 2020-06-01.D
样品名称：LJL-E-89-1-AD-H-40/60-asy-0.75-250

===

操作者　　　　：系统
仪器　　　　　：1260
位置　：样品瓶 1
进样日期　　　：2020-6-1 9:54:20
进样量　：没有进样
采集方法　　　：C:\CHEM32\1\METHODS\DEF_LC.M
最后修改　　　：2020-6-1 9:14:49 ：　系统
（调用后修改）
分析方法　　　：C:\CHEM32\1\METHODS\DEF_LC.M
附加信息：峰被手动积分

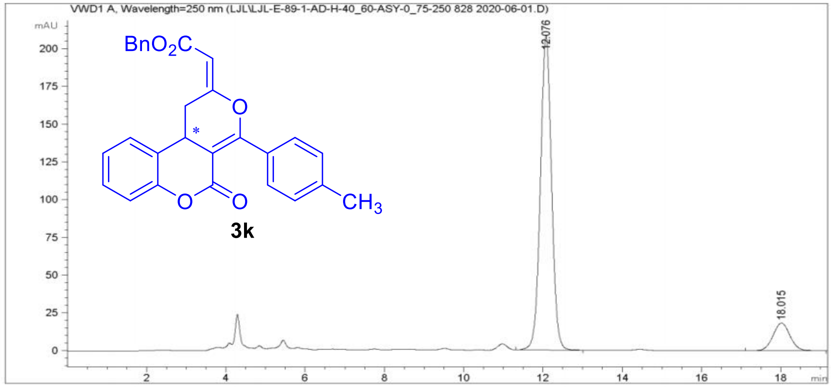

===

面积百分比报告

===

排序　　　：　信号
乘积因子　　：　1.0000
稀释因子　　：　1.0000
内标使用乘积因子和稀释因子

信号 1：VWD1 A, Wavelength=250 nm

峰 #	保留时间 [min]	类型	峰宽 [min]	峰面积 [mAU*s]	峰高 [mAU]	峰面积 %
1	12.076	BB	0.3021	4070.86206	208.21954	88.2262
2	18.015	BB	0.4596	543.25537	18.28586	11.7738

数据文件：C:\CHEM32\1\DATA\LJL\LJL-E-79-3-RAC-0_75-40_60-250-AD-H 693 2020-01-03.D
样品名称：ljl-e-79-3-rac-0.75-40/60-250-ad-h

==

操作者　　：系统
仪器　　：1260　　位置 ： 样品瓶 1
进样日期　　：2020-1-3 20:36:03
　　进样量 ： 没有进样
采集方法　　：C:\CHEM32\1\METHODS\DEF_LC.M
最后修改　　：2020-1-3 19:14:27 ： 系统
（调用后修改）
分析方法　　：C:\CHEM32\1\METHODS\DEF_LC.M
附加信息：峰被手动积分

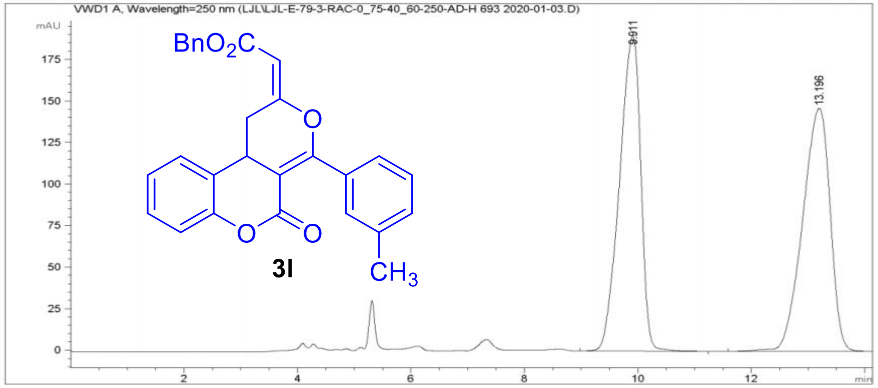

==
面积百分比报告
==

排序　　：　信号
乘积因子　　：　1.0000
稀释因子　　：　1.0000
内标使用乘积因子和稀释因子

信号 1: VWD1 A, Wavelength=250 nm

峰 #	保留时间 [min]	类型	峰宽 [min]	峰面积 [mAU*s]	峰高 [mAU]	峰面积 %
1	9.911	BB	0.4083	4945.97461	189.60741	50.0824
2	13.196	BBA	0.5293	4929.70898	146.24007	49.9176

数据文件：C:\CHEM32\1\DATA\LJL\LJL-E-79-3-ASY-0_75-40_60-250-AD-H 690 2020-01-03.D
样品名称：ljl-e-79-3-asy-0.75-40/60-250-ad-h

==

操作者　　：系统
仪器　　：1260　　位置 ： 样品瓶 1
进样日期　　：2020-1-3 19:40:33
　　进样量 ： 没有进样
采集方法　　：C:\CHEM32\1\METHODS\DEF_LC.M
最后修改　　：2020-1-3 19:14:27 ： 系统
（调用后修改）
分析方法　　：C:\CHEM32\1\METHODS\DEF_LC.M
附加信息：峰被手动积分

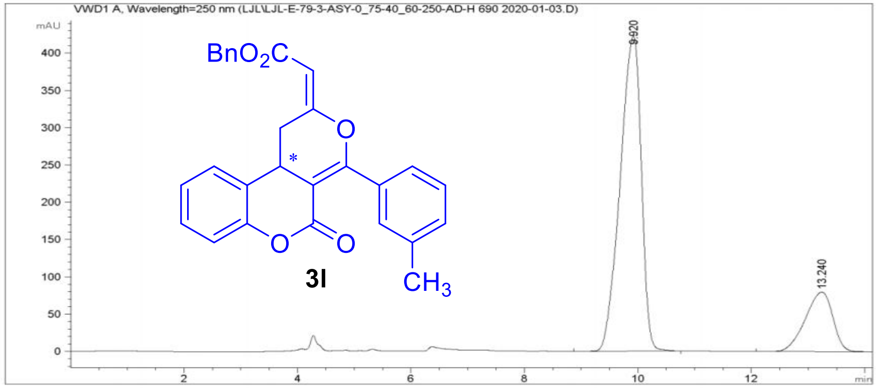

==
面积百分比报告
==

排序　　：　信号
乘积因子　　：　1.0000
稀释因子　　：　1.0000
内标使用乘积因子和稀释因子

信号 1: VWD1 A, Wavelength=250 nm

峰 #	保留时间 [min]	类型	峰宽 [min]	峰面积 [mAU*s]	峰高 [mAU]	峰面积 %
1	9.920	BB	0.4069	1.09385e4	422.69073	80.2480
2	13.240	BBA	0.5288	2692.37695	79.77031	19.7520

数据文件：C:\CHEM32\1\DATA\LJL\LJL-E-79-2-RAC-0_75-40_60-250-AD-H 687 2020-01-03.D
样品名称：ljl-e-79-2-rac-0.75-40/60-250-ad-h

操作者	：系统		
仪器	：1260	位置	：样品瓶 1
进样日期	：2020-1-3 16:57:38		
		进样量	：没有进样
采集方法	：C:\CHEM32\1\METHODS\DEF_LC.M		
最后修改	：2020-1-3 15:58:14 ： 系统 （调用后修改）		
分析方法	：C:\CHEM32\1\METHODS\DEF_LC.M		

附加信息：峰被手动积分

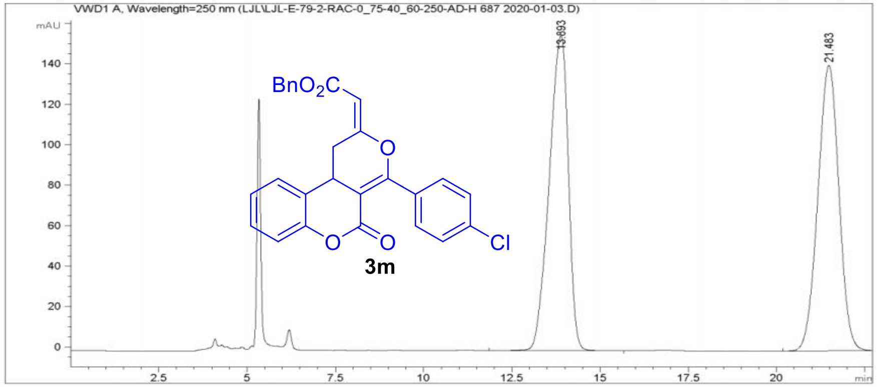

面积百分比报告

排序 ： 信号
乘积因子 ： 1.0000
稀释因子 ： 1.0000
内标使用乘积因子和稀释因子

信号 1：VWD1 A, Wavelength=250 nm

峰 #	保留时间 [min]	类型	峰宽 [min]	峰面积 [mAU*s]	峰高 [mAU]	峰面积 %
1	13.893	BB	0.5920	5888.79834	155.79689	50.1767
2	21.483	BBA	0.6456	5847.31250	141.06619	49.8233

1260 2020-1-3 20:41:48 系统　　页 1/2

数据文件：C:\CHEM32\1\DATA\LJL\LJL-E-79-2-ASY-0_75-40_60-250-AD-H 688 2020-01-03.D
样品名称：ljl-e-79-2-asy-0.75-40/60-250-ad-h

操作者	：系统		
仪器	：1260	位置	：样品瓶 1
进样日期	：2020-1-3 17:21:50		
		进样量	：没有进样
采集方法	：C:\CHEM32\1\METHODS\DEF_LC.M		
最后修改	：2020-1-3 15:58:14 ： 系统 （调用后修改）		
分析方法	：C:\CHEM32\1\METHODS\DEF_LC.M		

附加信息：峰被手动积分

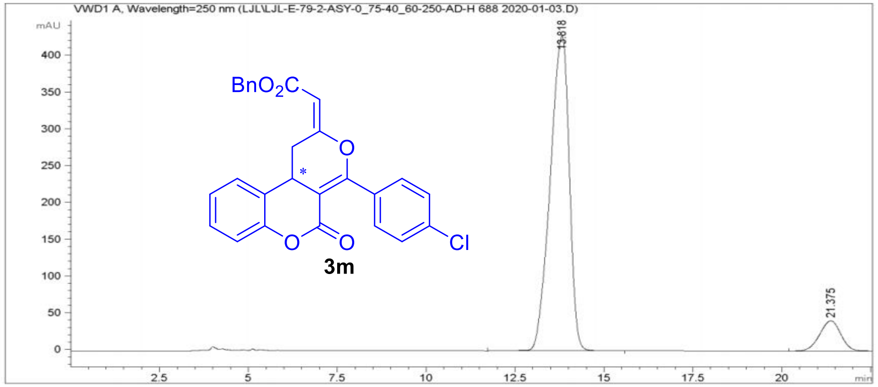

面积百分比报告

排序 ： 信号
乘积因子 ： 1.0000
稀释因子 ： 1.0000
内标使用乘积因子和稀释因子

信号 1：VWD1 A, Wavelength=250 nm

峰 #	保留时间 [min]	类型	峰宽 [min]	峰面积 [mAU*s]	峰高 [mAU]	峰面积 %
1	13.818	BB	0.5853	1.59864e4	428.42117	90.4598
2	21.375	BBA	0.6428	1685.96887	40.99793	9.5402

1260 2020-1-3 20:42:12 系统　　页 1/2

数据文件：C:\CHEM32\1\DATA\LJL\LJL-E-89-3-AD-H-40_60-RAC-0_75-250 833 2020-06-01.D
样品名称：LJL-E-89-3-AD-H-40/60-RAC-0.75-250

===

操作者 ： 系统
仪器 ： 1260 位置 ： 样品瓶 1
进样日期 ： 2020-6-1 21:38:40
进样量 ： 没有进样
采集方法 ： C:\CHEM32\1\METHODS\DEF_LC.M
最后修改 ： 2020-6-1 21:09:55 ： 系统
(调用后修改)
分析方法 ： C:\CHEM32\1\METHODS\DEF_LC.M
附加信息：峰被手动积分

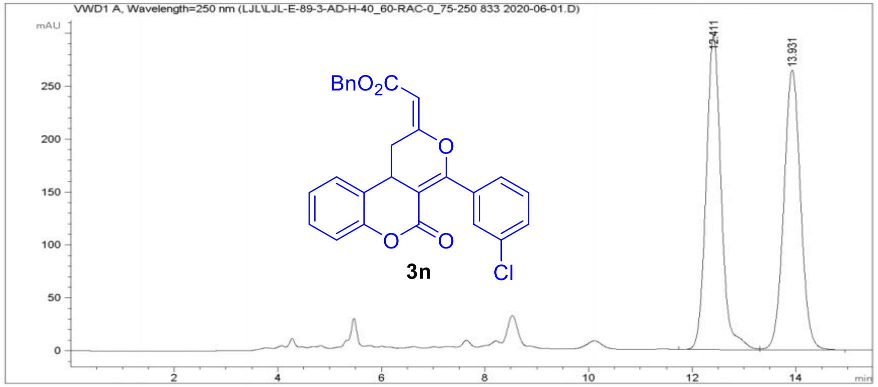

===
面积百分比报告
===

排序 ： 信号
乘积因子 ： 1.0000
稀释因子 ： 1.0000
内标使用乘积因子和稀释因子

信号 1：VWD1 A, Wavelength=250 nm

峰 #	保留时间 [min]	类型	峰宽 [min]	峰面积 [mAU*s]	峰高 [mAU]	峰面积 %
1	12.411	BV	0.3094	5986.52441	296.64508	50.2369
2	13.931	VB	0.3466	5930.05566	264.42966	49.7631

数据文件：C:\CHEM32\1\DATA\LJL\LJL-E-89-3-AD-H-40_60-ASY-0_75-250 812 2020-05-30.D
样品名称：LJL-E-89-3-AD-H-40/60-asy-0.75-250

===

操作者 ： 系统
仪器 ： 1260 位置 ： 样品瓶 1
进样日期 ： 2020-5-30 15:36:10
进样量 ： 没有进样
采集方法 ： C:\CHEM32\1\METHODS\DEF_LC.M
最后修改 ： 2020-5-30 15:05:28 ： 系统
(调用后修改)
分析方法 ： C:\CHEM32\1\METHODS\DEF_LC.M
附加信息：峰被手动积分

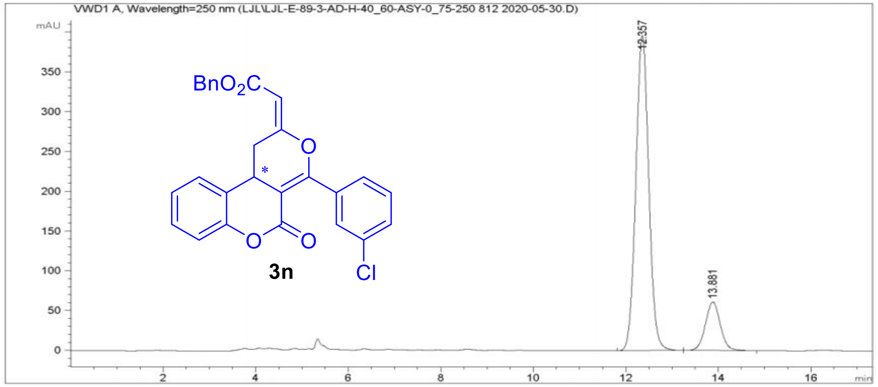

===
面积百分比报告
===

排序 ： 信号
乘积因子 ： 1.0000
稀释因子 ： 1.0000
内标使用乘积因子和稀释因子

信号 1：VWD1 A, Wavelength=250 nm

峰 #	保留时间 [min]	类型	峰宽 [min]	峰面积 [mAU*s]	峰高 [mAU]	峰面积 %
1	12.357	BB	0.3012	7706.71143	395.76303	84.8887
2	13.881	BB	0.3470	1371.89966	61.09722	15.1113

数据文件: C:\CHEM32\1\DATA\LJL\WKG-E-23-2-AD-40_60-RAC-0_75-250 879 2020-06-12.D
样品名称: wkg-e-23-2-ad-40/60-rac-0.75-250

操作者 : 系统
仪器 : 1260 位置 : 样品瓶 1
进样日期 : 2020-6-12 10:26:09
进样量 : 没有进样
采集方法 : C:\CHEM32\1\METHODS\DEF_LC.M
最后修改 : 2020-6-12 10:17:05 : 系统
(调用后修改)
分析方法 : C:\CHEM32\1\METHODS\DEF_LC.M
附加信息: 峰被手动积分

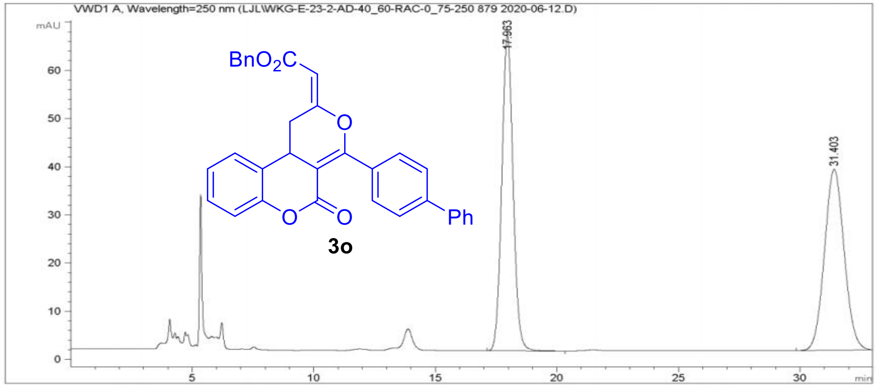

面积百分比报告

排序 : 信号
乘积因子 : 1.0000
稀释因子 : 1.0000
内标使用乘积因子和稀释因子

信号 1: VWD1 A, Wavelength=250 nm

峰 #	保留时间 [min]	类型	峰宽 [min]	峰面积 [mAU*s]	峰高 [mAU]	峰面积 %
1	17.963	BB	0.5013	2120.28345	65.39682	50.1111
2	31.403	BBA	0.8707	2110.87891	37.55721	49.8889

数据文件: C:\CHEM32\1\DATA\LJL\LJL-E-23-2-AD-40_60-ASY-0_75-250 880 2020-06-12.D
样品名称: ljl-e-23-2-ad-40/60-asy-0.75-250

操作者 : 系统
仪器 : 1260 位置 : 样品瓶 1
进样日期 : 2020-6-12 11:00:08
进样量 : 没有进样
采集方法 : C:\CHEM32\1\METHODS\DEF_LC.M
最后修改 : 2020-6-12 10:17:05 : 系统
(调用后修改)
分析方法 : C:\CHEM32\1\METHODS\DEF_LC.M
附加信息: 峰被手动积分

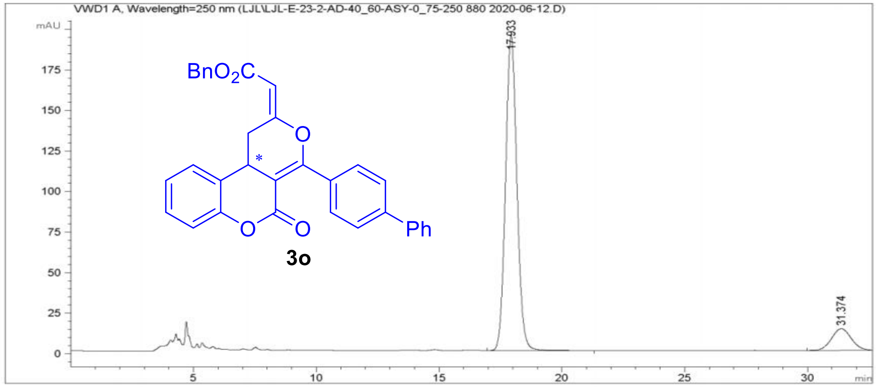

面积百分比报告

排序 : 信号
乘积因子 : 1.0000
稀释因子 : 1.0000
内标使用乘积因子和稀释因子

信号 1: VWD1 A, Wavelength=250 nm

峰 #	保留时间 [min]	类型	峰宽 [min]	峰面积 [mAU*s]	峰高 [mAU]	峰面积 %
1	17.933	BB	0.4975	6262.58643	194.06117	89.4091
2	31.374	BBA	0.8491	741.83203	13.35412	10.5909

数据文件: C:\CHEM32\1\DATA\LJL\LJL-G-35-1-ASY-AD-H-40_60-0_75-205 201 2020-10-08.D
样品名称: ljl-g-35-1-ASY-ad-h-40/60-0.75-205

===

操作者	: 系统		
仪器	: 1260	位置	: 样品瓶 1
进样日期	: 2020-10-8 15:14:13		
		进样量	: 没有进样
采集方法	: C:\CHEM32\1\METHODS\DEF_LC.M		
最后修改	: 2020-10-8 15:04:41 : 系统 (调用后修改)		
分析方法	: C:\CHEM32\1\METHODS\DEF_LC.M		

附加信息: 峰被手动积分

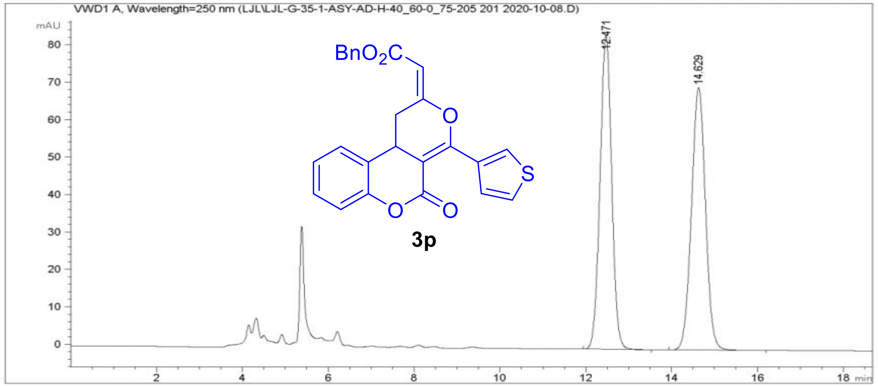

===
面积百分比报告
===

排序	:	信号
乘积因子	:	1.0000
稀释因子	:	1.0000

内标使用乘积因子和稀释因子

信号 1: VWD1 A, Wavelength=250 nm

峰 #	保留时间 [min]	类型	峰宽 [min]	峰面积 [mAU*s]	峰高 [mAU]	峰面积 %
1	12.471	BB	0.2941	1586.92017	83.73536	49.9485
2	14.629	BB	0.3529	1590.19189	70.03180	50.0515

1260 2020-10-8 16:10:33 系统 页 1/2

数据文件: C:\CHEM32\1\DATA\LJL\LJL-G-35-1-ASY-AD-H-40_60-0_75-250 202 2020-10-08.D
样品名称: ljl-g-35-1-ASY-ad-h-40/60-0.75-250

===

操作者	: 系统		
仪器	: 1260	位置	: 样品瓶 1
进样日期	: 2020-10-8 15:34:46		
		进样量	: 没有进样
采集方法	: C:\CHEM32\1\METHODS\DEF_LC.M		
最后修改	: 2020-10-8 15:04:41 : 系统 (调用后修改)		
分析方法	: C:\CHEM32\1\METHODS\DEF_LC.M		

附加信息: 峰被手动积分

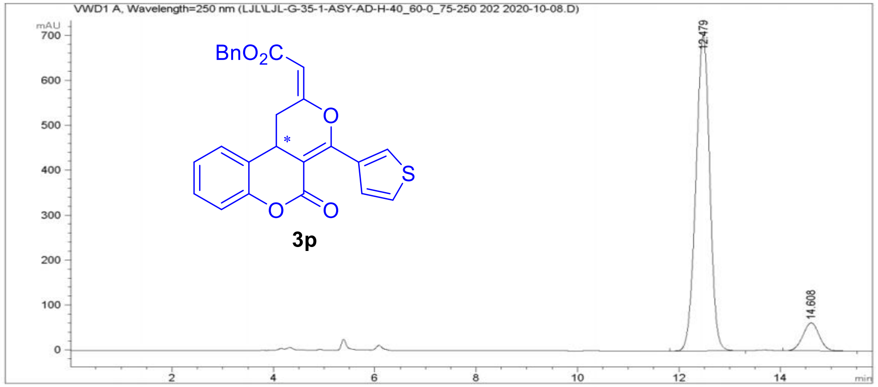

===
面积百分比报告
===

排序	:	信号
乘积因子	:	1.0000
稀释因子	:	1.0000

内标使用乘积因子和稀释因子

信号 1: VWD1 A, Wavelength=250 nm

峰 #	保留时间 [min]	类型	峰宽 [min]	峰面积 [mAU*s]	峰高 [mAU]	峰面积 %
1	12.479	BB	0.2972	1.33548e4	701.34009	90.4910
2	14.608	VB	0.3489	1403.35583	62.50670	9.5090

1260 2020-10-8 16:10:19 系统 页 1/2

数据文件：C:\CHEM32\1\DATA\LJL\LJL-G-92-2-RAC-OD-3-20_80-0_75-250 599 2021-01-06.D
样品名称：ljl-g-92-2-rac-od-3-20/80-0.75-250

===

操作者 ：系统
仪器 ：1260　　位置 ：样品瓶 1
进样日期 ：2021-1-6 16:02:56
　　进样量 ：没有进样
采集方法 ：C:\CHEM32\1\METHODS\DEF_LC.M
最后修改 ：2021-1-6 15:19:57 ：系统
（调用后修改）
分析方法 ：C:\CHEM32\1\METHODS\DEF_LC.M
附加信息：峰被手动积分

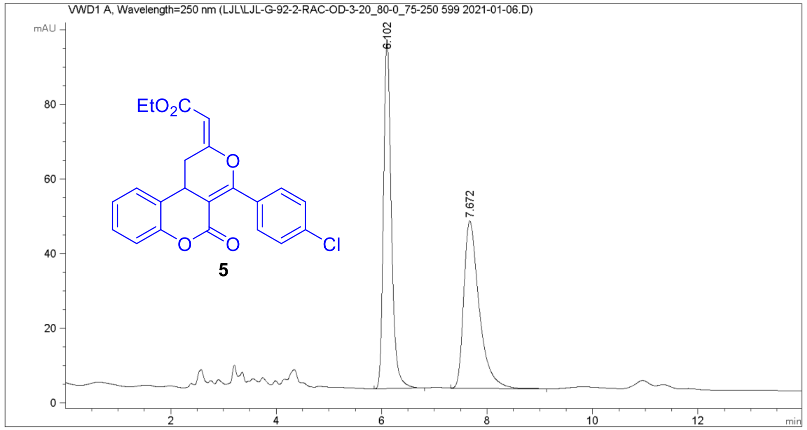

===
面积百分比报告
===

排序 ：信号
乘积因子 ：1.0000
稀释因子 ：1.0000
内标使用乘积因子和稀释因子

信号 1：VWD1 A, Wavelength=250 nm

峰 #	保留时间 [min]	类型	峰宽 [min]	峰面积 [mAU*s]	峰高 [mAU]	峰面积 %
1	6.102	BB	0.1578	967.98108	93.48794	51.5435
2	7.672	VB	0.3047	910.00598	44.86181	48.4565

数据文件：C:\CHEM32\1\DATA\LJL\LJL-G-99-1-ASY-OD-3-20_80-0_75-250 598 2021-01-06.D
样品名称：ljl-g-99-1-asy-od-3-20/80-0.75-250

===

操作者 ：系统
仪器 ：1260　　位置 ：样品瓶 1
进样日期 ：2021-1-6 15:53:39
　　进样量 ：没有进样
采集方法 ：C:\CHEM32\1\METHODS\DEF_LC.M
最后修改 ：2021-1-6 15:19:57 ：系统
（调用后修改）
分析方法 ：C:\CHEM32\1\METHODS\DEF_LC.M
附加信息：峰被手动积分

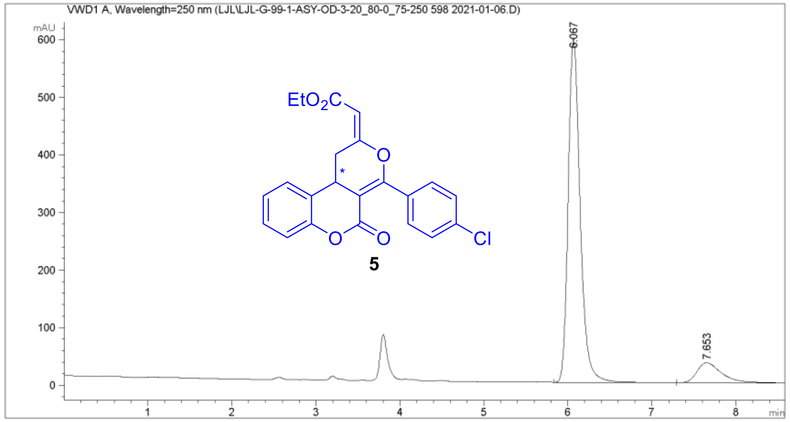

===
面积百分比报告
===

排序 ：信号
乘积因子 ：1.0000
稀释因子 ：1.0000
内标使用乘积因子和稀释因子

信号 1：VWD1 A, Wavelength=250 nm

峰 #	保留时间 [min]	类型	峰宽 [min]	峰面积 [mAU*s]	峰高 [mAU]	峰面积 %
1	6.067	VB	0.1505	5903.75146	596.50482	89.4155
2	7.653	BBA	0.3031	698.85535	34.67631	10.5845
